# Strategies to Augment the Cardiovascular System and Acutely Enhance Exercise Performance in Individuals with Spinal Cord Injury: A Systematic Scoping Review

**DOI:** 10.1186/s40798-025-00909-7

**Published:** 2025-11-06

**Authors:** Daniel D. Hodgkiss, Shane J. T. Balthazaar, Cameron M. Gee, Shin-Yi Chiou, Samuel J. E. Lucas, Tom E. Nightingale

**Affiliations:** 1 School of Sport, Exercise and Rehabilitation Sciences, College of Life and Environmental Sciences, University of Birmingham, Edgbaston, Birmingham, UK; 2 International Collaboration on Repair Discoveries, Faculty of Medicine, University of British Columbia (UBC), Vancouver, BC Canada; 3 Department of Cardiology, University Hospitals Birmingham National Health Service (NHS) Foundation Trust, Birmingham, UK; 4 Department of Orthopaedics, Faculty of Medicine, University of British Columbia, Vancouver, BC Canada; 5 Centre for Human Brain Health, University of Birmingham, Birmingham, UK; 6 MRC Versus Arthritis Centre for Musculoskeletal Ageing Research, University of Birmingham, Birmingham, UK

## Abstract

**Background:**

Spinal cord injury (SCI) affects motor and autonomic functions that reduce exercise capacity. Specifically, the loss of sympathetic drive following SCI at or above the sixth thoracic segment (≥ T6) can impair cardiovascular responses to exercise. This systematic scoping review aimed to identify ergogenic strategies that may augment the cardiovascular system and acutely enhance exercise performance in individuals with SCI.

**Methods:**

A systematic literature search was conducted using electronic databases (Medline, Embase, Web of Science) from inception to 1st April 2025. Studies were included if they met the following eligibility criteria: (1) human participants (aged ≥ 16 years); (2) any acquired SCI (traumatic, infection, cancer); (3) any sample size but must be > 80% SCI; (4) acute, single, volitional exercise sessions with cross-over design (i.e., ergogenic strategy and control sessions); (5) report a measurable exercise performance outcome, and (6) the strategy used to enhance performance must have a theoretical effect on the cardiovascular system. Data were extracted from eligible studies and charted. Hedges’* g* summary effect sizes were calculated to quantify the magnitude of effects across strategies.

**Results:**

A total of 7266 possible articles were identified. Following a full-text review, 32 articles were included. Findings were reported by strategy, defined as either mechanical (e.g., abdominal binders, lower-body compression, passive leg exercise and supine posture) or neuromodulatory [e.g., autonomic dysreflexia (AD), functional electrical stimulation (FES), pharmaceuticals/supplements/stimulants, and spinal cord stimulation (SCS)]. The neuromodulatory strategies appeared more robust at augmenting cardiovascular and performance outcomes, particularly AD, FES, and SCS.

**Conclusions:**

We examined methods to improve acute exercise performance by augmenting the cardiovascular system in individuals with SCI. The large heterogeneity across methodologies and outcome measures made it challenging to draw conclusions regarding the underlying physiological mechanisms. Consequently, providing definitive recommendations on the best strategies to enhance performance was not possible based on current literature. Future research should be conducted across all ergogenic strategies, with a careful focus on females, trained and untrained participants, and individuals who are more likely to benefit from improvements in cardiovascular output (i.e., SCI ≥ T6).

*Registration* This review was pre-registered on the Open Science Framework (https://osf.io/w7apu/).

**Supplementary Information:**

The online version contains supplementary material available at 10.1186/s40798-025-00909-7.

## Key Points

- Strategies that exploit neuromodulatory mechanisms (e.g., spinal cord stimulation, boosting via autonomic dysreflexia, and functional electrical stimulation) alter autonomic cardiovascular function and improve exercise capacity.
- A broad scope of testing using functional electrical stimulation has been reported, but study methodology and protocols are inconsistent across the literature making it difficult to draw conclusions for this ergogenic strategy specifically.
- The majority of studies recruited middle-aged males with a motor-complete, chronic spinal cord injury. Data focusing on enhancing exercise performance in females with spinal cord injury are sparse (only 6% of participants included in this review were female), as are the effects of ergogenic strategies in the acute and sub-acute spinal cord injury setting, and should therefore be a focus of future research. Further, long-term studies are needed to assess the efficacy, safety and practicality of these interventions in real-world settings.

**Supplementary Information:**

The online version contains supplementary material available at 10.1186/s40798-025-00909-7.

## Background

Spinal cord injury (SCI) is a complex and devastating neurological condition characterised by a loss of sensory, motor and autonomic function below the level of injury that profoundly impacts health-related quality of life. Among the myriad of autonomic impairments resulting from SCI [1, 2], the restoration of cardiovascular function is considered an important target of recovery for this population [3].

The autonomic nervous system (ANS) plays a role in regulating cardiovascular responses to exercise [4], but this regulation is typically disrupted following SCI [5–7]. Sympathetic pre-ganglionic neurons (SPNs) located between the first and fifth thoracic spinal segments (T1–T5) innervate the heart and upper-body vasculature, whereas the SPNs exiting the spinal cord between the first thoracic and second lumbar spinal segments (T1–L2) innervate the major vascular beds of the gut and lower extremities. SCI at or above these levels may disrupt descending sympathetic pathways between the cardiovascular centres in the brainstem and the SPNs, thereby inhibiting supraspinal drive to the cardiovascular system [8, 9]. Indeed, neurological level of injury is related to the degree of cardiovascular dysfunction [10], whereby individuals with cervical or upper-thoracic SCI exhibit more impaired cardiovascular responses to exercise in comparison to individuals with lower-level injuries [11, 12]. In these individuals, parasympathetic withdrawal is often the primary means of increasing heart rate (HR) during exercise given that parasympathetic innervation remains intact via the vagus nerve. Notably, athletes with tetraplegia who exhibit partial to full sparing of descending sympathetic pathways are at a performance advantage, despite a similar degree of motor impairment [7]; which is an important observation given that cardiovascular instability cannot always be predicted by motor-sensory level and completeness of an SCI [13]. Moreover, the activation of these otherwise dormant spinal sympathetic pathways leads to improvements in cardiovascular function [14–20]. Strategies to overcome cardiovascular limitations and acutely enhance aerobic exercise capacity may provide athletes with an advantage in sport settings or be beneficial in exercise rehabilitation settings.

Cardiac function following SCI is likely to be impaired via a combination of reduced venous return due to the loss of centrally mediated sympathetic vasoconstriction below the level of the injury [21], loss of the skeletal muscle pump [22], an impaired respiratory muscle pump [23], impaired cardiac contractility [20], and reduced blood volume [24]. Collectively, this results in decreased left-ventricular end-diastolic volume and preload [25] that in turn impairs filling and compromises left-ventricular stroke volume through Frank–Starling mechanisms. While these alterations in cardiac function are likely insignificant at rest, they may contribute to limiting oxygen transport and perfusion to working skeletal muscles during exercise and thus limit the aerobic capacity of individuals with SCI. Furthermore, during exercise these individuals typically exhibit a limitation in peak HR (HR_peak_; ~ 110–130 bpm) [26], a lower oxygen pulse (which is a surrogate for stroke volume) [7, 27, 28], lower circulating catecholamines [29, 30], and a limited capacity to redirect oxygen-rich blood to exercising muscles via sympathetically mediated splanchnic vasoconstriction that results in blood pooling in the abdomen and reduced venous return [31]. Subsequently, left-ventricular stroke volume does not increase with exercise to the same magnitude as would be expected in non-SCI individuals with intact supraspinal sympathetic drive to the heart and vasculature [31, 32]. This seemingly has implications for aerobic exercise performance given that cardiac output is the primary determinant of peak oxygen uptake (${{\dot{\text{V}}}{\text{O}}}_{{2{\text{peak}}}}$) in comparison to other key oxygen transport variables [33]. Therefore, strategies that can acutely enhance venous return and/or augment left-ventricular stroke volume may be particularly effective in enhancing exercise capacity in individuals with SCI.

Importantly, cardiovascular dysfunction during inpatient rehabilitation may negatively impact engagement in physical activity [34] and, following discharge, individuals with SCI are also less likely to participate in physical activity [35]. Only 1 in 4 individuals report that they have the necessary functional capacity to live independently [36] and such challenges result in a harmful domino effect whereby a reduction in regular physiological stimuli leads to progressive physical deconditioning that is associated with a low level of cardiorespiratory fitness [37, 38]. This low level of fitness can cause problems engaging with activities of daily living [39–42] and exacerbate chronic fatigue [43], thereby further increasing physical inactivity and sedentary behaviour [44, 45]. This ultimately heightens the risk of chronic disease and mortality [46–49]. While it is known that regular exercise may break this downward spiral and augment cardiorespiratory fitness [50], there is a clear need for approaches that optimise cardiovascular responses to exercise acutely. Strategies that enhance venous return, left-ventricular end-diastolic volume, and stroke volume may not only attenuate the SCI-induced changes in cardiac function but may also offset the cardiac output-mediated limits to aerobic exercise capacity in individuals with SCI [51]. Such acute improvements in exercise capacity may therefore improve engagement with exercise training. Thus understanding and optimising the acute exercise response in this population could have longer-term benefits.

Several reviews have evaluated the effects of ergogenic strategies in the general, non-SCI population [52–55]. However, to the best of our knowledge no study has focused on their use in the SCI population specifically, and, in particular, aids that are theoretically designed to augment the cardiovascular system. Therefore, this systematic scoping review aimed to comprehensively search the available literature for strategies that are designed to augment the cardiovascular system and acutely enhance exercise performance in individuals with SCI. Additionally, we aimed to quantify the magnitude of effect for each strategy in an attempt to provide recommendations on the most optimal approach to supplement exercise training, performance and/or rehabilitation in individuals with SCI.

## Methods

This systematic scoping review followed the five-stage scoping review methodological framework of Arksey and O’Malley [56], alongside the Preferred Reporting Items for Systematic Reviews and Meta-Analysis extension for Scoping Reviews (PRISMA-ScR) [57]. The protocol for this review was prospectively registered on the Open Science Framework on 19th April 2023 and can be found at https://osf.io/w7apu/.

### Identifying the Research Question

The authors formulated the primary research question: “*What is known from the existing literature on strategies that target the cardiovascular system to acutely enhance exercise performance in individuals with a spinal cord injury?*”.

### Identifying Relevant Studies

The electronic databases of Medline (via Ovid), Excerpta Medica Database (EMBASE; via Ovid), and Web of Science were searched systematically from their respective inception through to 1st April 2025. A coherent search strategy was developed (DDH, SJTB, CMG, TEN) and was refined in accordance with the PICO tool [58], which combined terms relating to the following: (1) population (e.g., spinal cord injury, tetraplegia, paraplegia, quadriplegia, paralysis); (2) exercise/performance (e.g., exercise, physical activity, sport, paralympic, cardiorespiratory fitness, power output, time trial); (3) the cardiovascular system [e.g., HR, blood pressure (BP), stroke volume, venous return, cardiac output, haemodynamic, oxygen extraction], and (4) ergogenic strategies [e.g., functional electrical stimulation (FES), boosting, autonomic dysreflexia, abdominal binder, pharmaceutical, stimulant]. No search filters were applied to any of the searches. Details of the complete search strategy are provided in the online supplementary material (Supplementary Tables S1 and S2).

### Study Selection

All database records were uploaded to the Covidence web-based review software (Covidence, Melbourne, Australia). Following the removal of duplicates, two authors (DDH, SJTB, and/or CMG) independently screened records by title and abstract, and then full-text. Studies that could not be removed during the title or abstract screening stage were retained and evaluated via a full-text review. All conflicts regarding study inclusion/exclusion criteria were resolved through discussion with the corresponding author (TEN). Every effort was made to obtain inaccessible full-text articles, including web searching, Interlibrary Loan services and contacting corresponding authors via email.

Studies were required to meet the following inclusion criteria: (1) human participants (aged ≥ 16 years); (2) any acquired SCI (traumatic, infection, cancer); (3) any sample size but must be > 80% SCI (i.e., samples may have included other neurological conditions or diseases); (4) acute, single, volitional exercise sessions with cross-over designs (i.e., ergogenic strategy and control sessions); (5) report a measurable exercise performance outcome, and (6) the strategy used to enhance performance must have a theoretical effect on the cardiovascular system.

Studies were excluded if they met the following criteria: (1) non-human; (2) non-original work (i.e., review articles, conference abstracts/posters, unpublished research, study protocols, guideline documents, editorials, letter-to-editors, viewpoints); (3) children or adolescents (aged < 16 years); (4) individuals without SCI (i.e., non-SCI participants or other neurological conditions); (5) does not report a performance outcome; (6) effects on performance are due to a longer-term therapeutic intervention, training or a supplementation period; (7) no suitable comparison [i.e., must have performance outcome data with and without an ergogenic aid, or exercise was not volitional (i.e., both ergogenic and control strategies utilised passive lower-limb or FES exercise only without an upper-body volitional component)]; (8) ergogenic aid used was not related to the theoretical augmentation of the cardiovascular system; (9) no full text available, and (10) not written in English. Where multiple publications had sampled the same participants, data were extracted from the most recent publication or the one with the most relevant outcomes.

### Charting of the Data

Data were extracted from all eligible studies using a template developed by the authors. Data were charted independently and agreed upon by two of three authors (DDH, SJTB and/or CMG). Any disagreements were resolved by discussion among the authors or by the corresponding author (TEN) where necessary.

The following data were charted:

- *Study characteristics*—first author, year of publication, country of origin, purpose/rationale for the ergogenic strategy, notable limitations, key findings and conclusions
- *Participant characteristics*—sample size, sex, age, time since injury, level of injury, injury severity, physical activity level
- *Ergogenic and control strategies*—description and methodological details
- *Exercise protocol*—modality, intensity, duration
- *Outcome measures*—any relevant performance or psychophysiological measures

### Collating, Summarising, and Reporting the Results

A two-part approach was taken to synthesise the extracted data. First, data were subjected to a numerical synthesis, whereby information was collated on article characteristics, participant characteristics/demographics, ergogenic strategy type and outcome measures (cardiovascular and performance). Where possible, details on adverse events were also reported. Second, reviewers identified key themes across studies to summarise the extracted data with respect to the research question. Risk of bias assessments and a critical appraisal of the literature were not conducted, as per guidance by Arksey and O’Malley [56].

Where required, means ± standard deviation (SD) were estimated from median and interquartile range [59] or median and range [60]. Data were extrapolated from figures using Photoshop (Adobe, San Jose, CA, USA). Oxygen pulse was calculated from ${{\dot{\text{V}}}{\text{O}}}_{2}$ and HR data, where possible. Participant demographics [i.e., age and time since injury (TSI)] were summarised using weighted means (range of lowest to highest mean values reported from studies). Weighted means were calculated to account for differences in sample size between studies using the following formula: Σn * ${\overline{\text{x}}}$/Σn, where Σ = the sum of, n = number of participants in each study and, ${\overline{\text{x}}}$ = mean variable (e.g., age or TSI) of each study.

To quantify the effects of an ergogenic strategy relative to its control, standardised effect sizes (Hedges’ *g*) were calculated for individual cardiovascular and performance outcomes within studies. These effect sizes were subsequently pooled and calculated as Hedges’ *g* with 95% confidence intervals (CI) using the *meta* package [61] in R (Version 3.5.1, R Foundation for Statistical Computing, Vienna, Austria) to compare ergogenic strategies. Magnitude thresholds of ≥ 0.2, ≥ 0.5 and ≥ 0.8 represent small, medium and large effects, respectively [62]. Only data as close to peak as possible were extracted for the calculation of effect sizes for cardiovascular outcomes (i.e., where studies performed at intensities corresponding to 40, 60 and 80% ${{\dot{\text{V}}}{\text{O}}}_{{2{\text{peak}}}}$, only the outcome data for 80% ${{\dot{\text{V}}}{\text{O}}}_{{2{\text{peak}}}}$ were extracted). For the calculation of performance outcome (e.g., ${{\dot{\text{V}}}{\text{O}}}_{2}$ and power output) pooled effect sizes, only peak data were extracted from individual studies. In instances where an effect size could not be calculated (i.e., case-reports, or SD was not reported/could not be determined), effects were calculated as percentage change.

## Results

### Study Selection

**Fig. 1:**
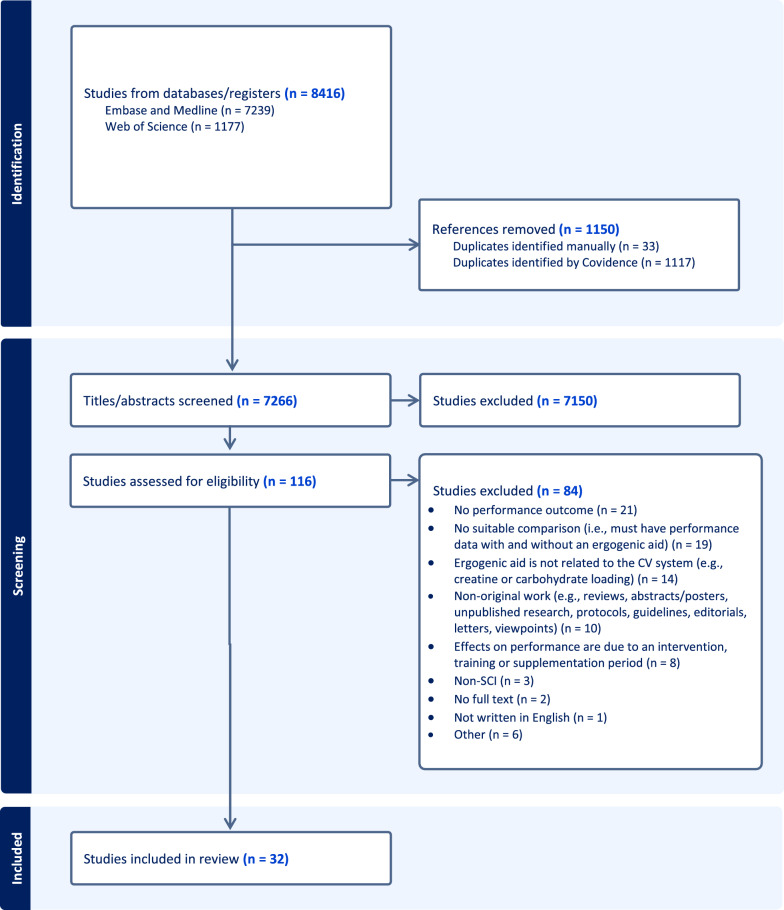
PRISMA flow diagram

The database search yielded a total of 7266 unique citations following the removal of 1150 duplicates. Following the screening of titles and abstracts for inclusion/exclusion criteria, 116 full texts were retrieved and assessed for eligibility. Of these, 32 unique citations were included in the data extraction process (Fig. 1).

### Article Characteristics

**Fig. 2:**
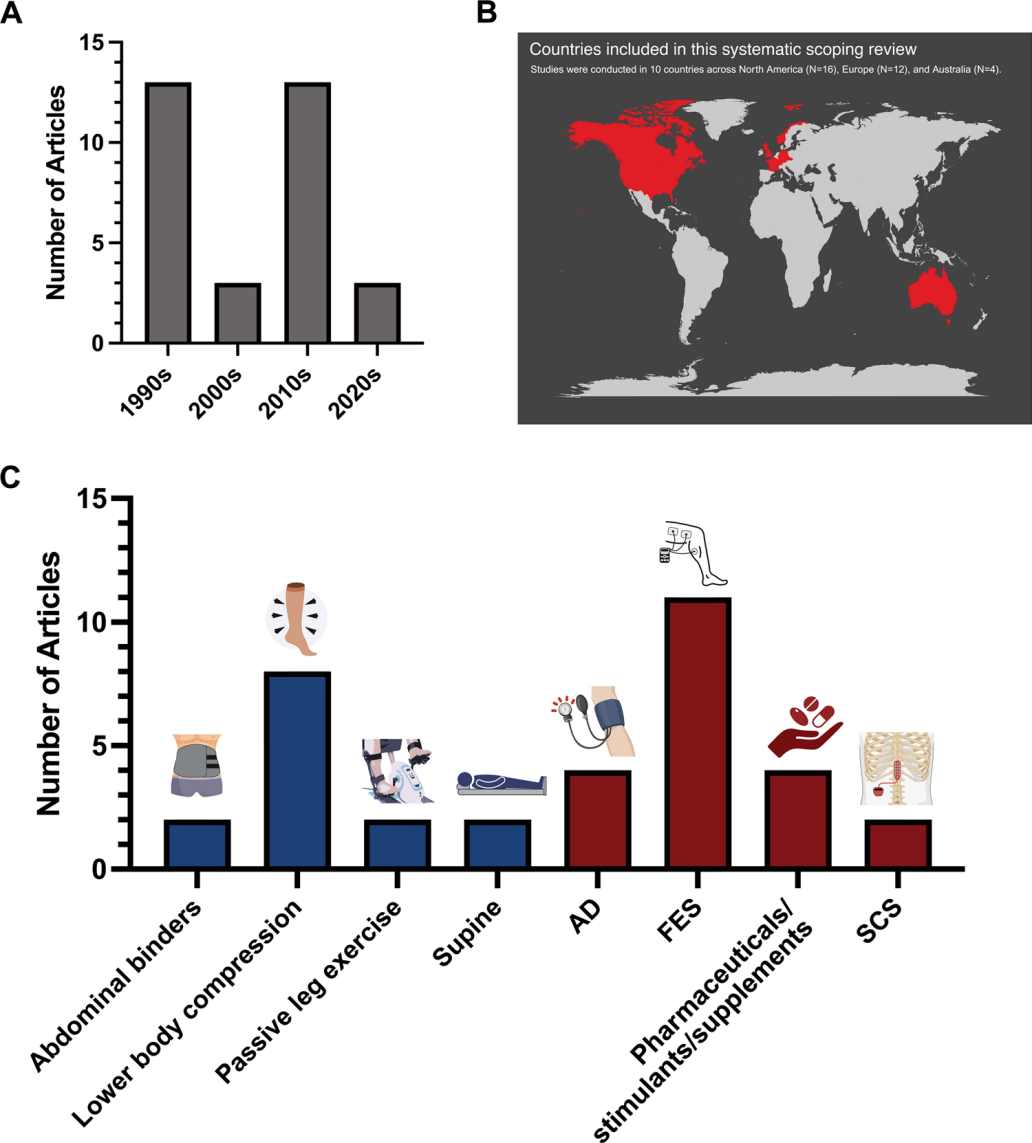
Publication trends by decade (**A**) and country (**B**). Primary affiliations and information within the articles were utilised to identify the countries in which each study was conducted. (**C**) The number of studies reporting on each mechanical (blue) and neuromodulatory (red) ergogenic strategy included within the review. Some studies investigated multiple ergogenic strategies, hence the greater total number of strategies in comparison to number of studies. *AD* autonomic dysreflexia, *FES* functional electrical stimulation, *SCS* spinal cord stimulation. Figure was created using Graphpad Prism (Version 9, GraphPad Software, Boston, MA, USA) and Biorender (Toronto, ON, Canada)

The full data extraction tables for all 32 included studies are provided as online supplementary material (Supplementary Tables S3 and S4). Publication trends by year and country, and the number of identified strategies, are presented in Fig. 2.

### Participant Characteristics/Demographics

Across the 32 studies, there were a total of 289 participants (6% female). Mean age of participants was 33 (range 22–44) years, while mean TSI was 11 (range 4–24) years. Age and TSI were either not reported or could not be determined in one [63] and six [63–68] studies, respectively. In total, there were 97 individuals with tetraplegia (ranging from C3 to C8) and 141 individuals with paraplegia (ranging from T1 to L4). Neurological level of injury could not be determined in three studies [69–71]. Whilst the majority of participants were motor-complete (86%), injury severity could not be determined in eight studies [63–66, 68, 69, 72, 73]. With regards to physical activity levels, three studies included inactive or sedentary individuals [74–76], six included recreationally or moderately active individuals [69, 70, 77–80], eleven included well-trained, national or elite-level athletes [30, 63, 65, 68, 71, 72, 81–85], and four included individuals of mixed physical activity levels [86–89]. However, physical activity levels were either not reported or could not be determined in eight studies [64, 66, 67, 73, 90–93].

### Ergogenic Strategies

**Fig. 3:**
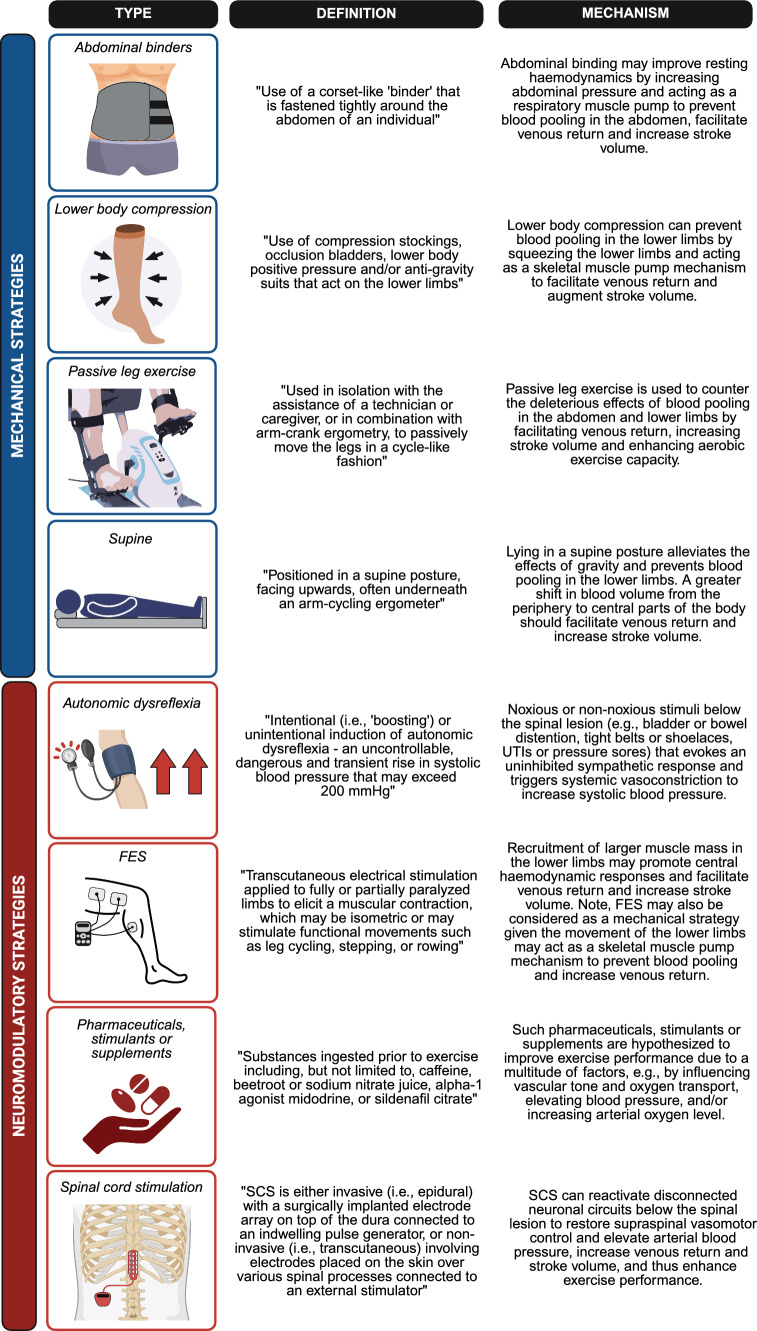
Summary of the mechanical (blue) and neuromodulatory (red) ergogenic strategies with definitions and proposed mechanisms of action to augment the cardiovascular system and acutely enhance exercise performance. *FES* functional electrical stimulation. Figure was created using Biorender (Toronto, ON, Canada)

Please refer to Fig. 3 for an overview of the ergogenic strategies included in this review. Strategies were collated into two types: (1) mechanical (i.e., strategies that aim to prevent blood pooling in the lower limbs and abdomen by the mobilisation of the lower limbs or application of an external aid), and (2) neuromodulatory (i.e., strategies that aim to act on the nervous system, to elicit a normalised sympathetic response to exercise). There were a total of fourteen mechanical strategies identified, of which two utilised passive leg exercise [70, 80], two used abdominal binders [81, 82], and eight utilised lower-body compression (specifically, two with occlusion cuffs/bladders [79, 86], four with anti-gravity suits [65, 72, 78, 90] and two with socks or stockings [83, 88]). Two studies also included exercising whilst supine [74, 78].

Twenty-one neuromodulatory strategies were identified, of which eleven utilised FES. Of these FES studies, two combined upper-body exercise with static lower-body FES [i.e., neuromuscular electrical stimulation (NMES)] [91, 92] and nine utilised upper-body exercise combined with FES-induced leg cycling or hybrid ergometry [64, 66, 67, 69, 70, 73, 77, 92, 93]. Four studies reported on the effects of intentional (i.e., boosting; triggered via bladder distention) [30, 63] or unintentional induction of autonomic dysreflexia (AD) [76, 85], four studies utilised pharmaceuticals, supplements or stimulants (i.e., midodrine [87], caffeine [84], sildenafil citrate [71], sodium nitrate [68] and beetroot juice [68]), two studies utilised epidural spinal cord stimulation (SCS) [75, 89], and one transcutaneous SCS [89].

Note, some studies included multiple ergogenic strategies hence the greater total number of strategies versus studies. The exercise modalities, protocols and further details for each of the ergogenic and control strategies within each study can be found in the supplementary material (Supplementary Tables S3 and S4).

### Outcome Measures

A summary of the studies reporting on specific performance and cardiovascular outcomes for the mechanical and neuromodulatory ergogenic strategies can be found in Tables 1 and 2, respectively.

**Table 1:**
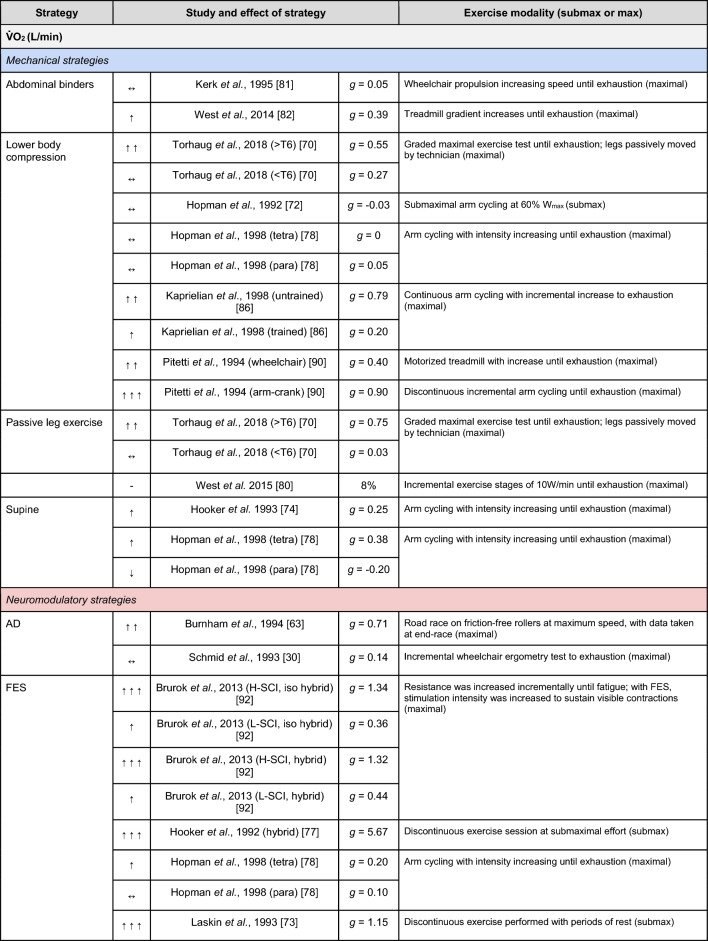

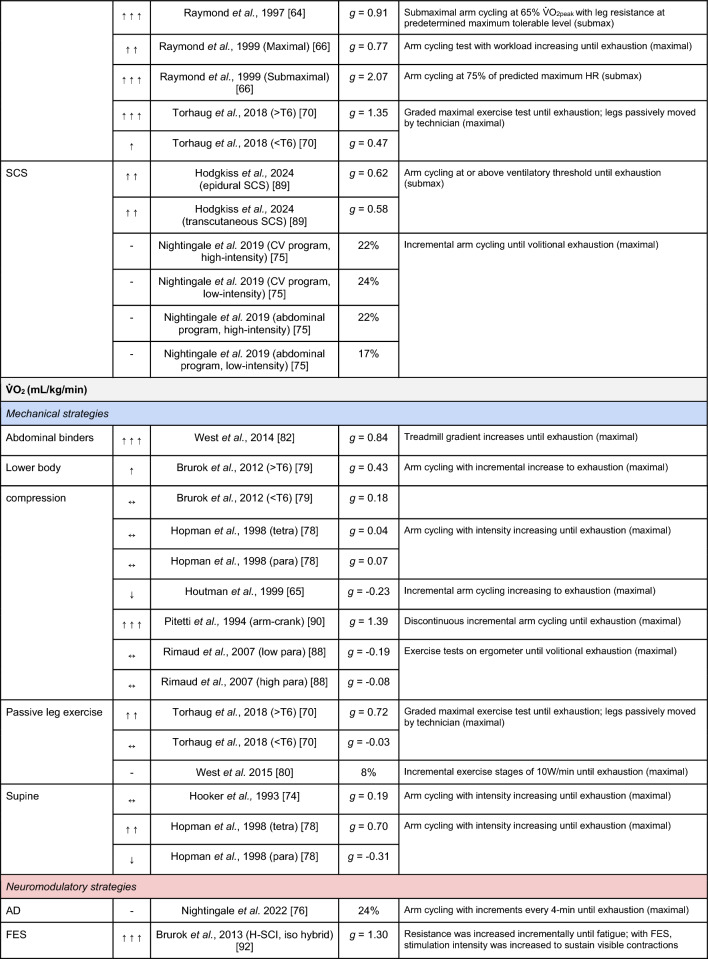

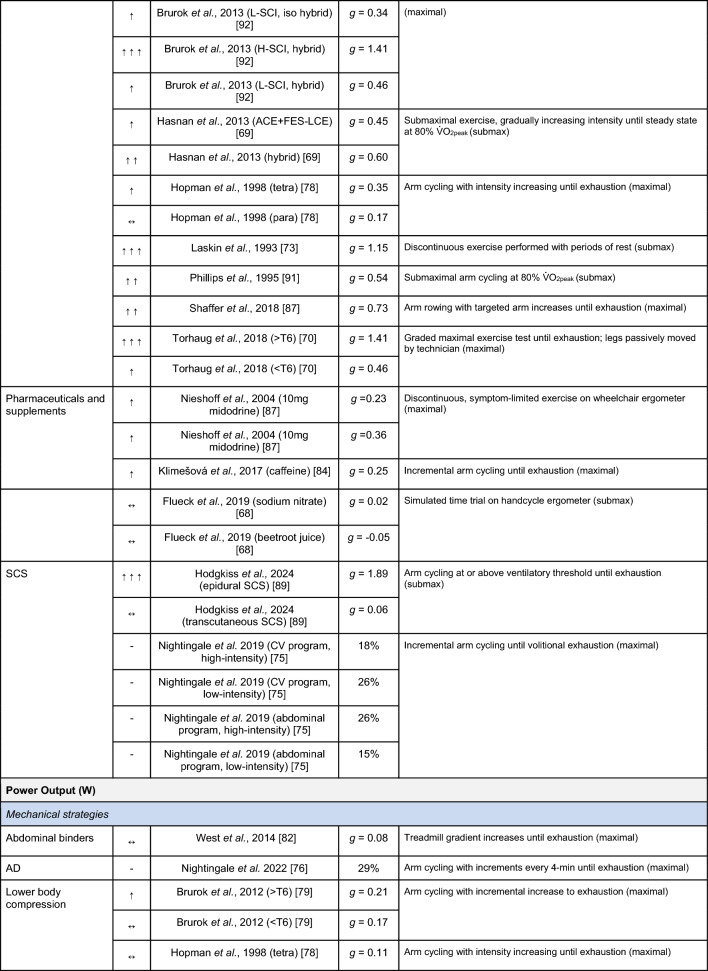

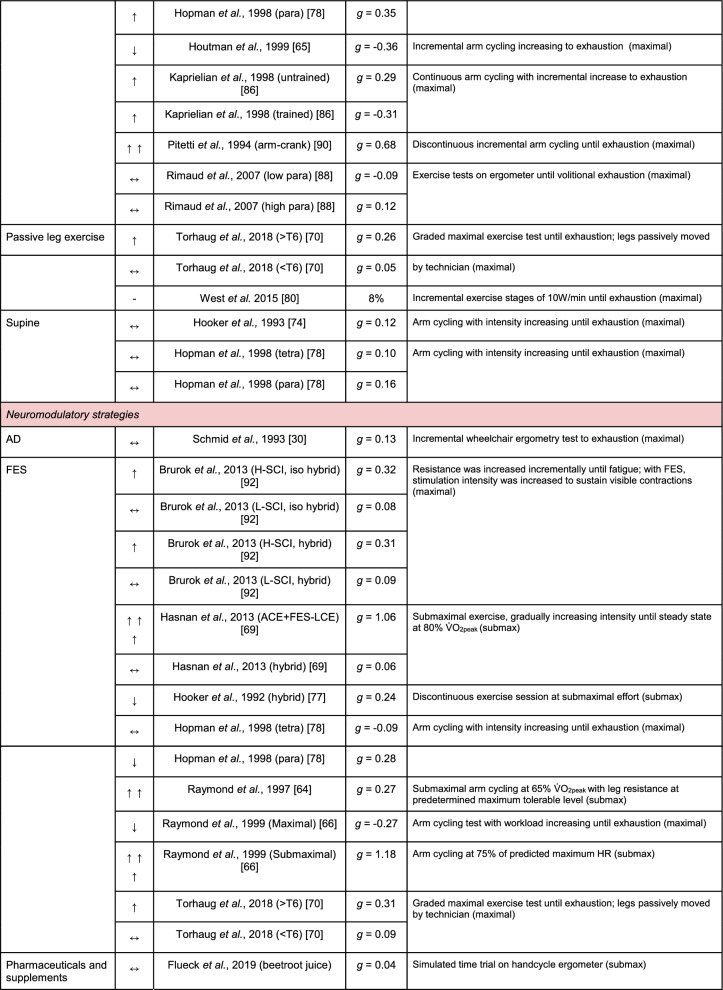

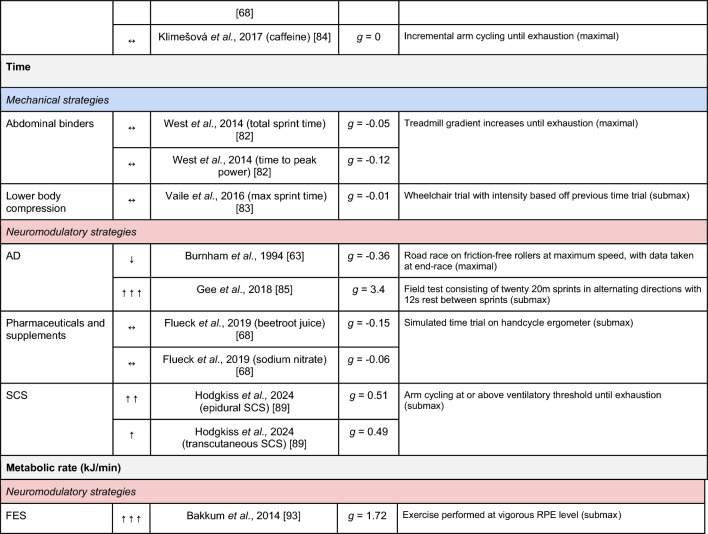
Effects of individual mechanical and neuromodulatory strategies on performance outcomes

Hedges’ *g* effect sizes are calculated for each individual study. Where studies reported outcomes for multiple exercise intensities (e.g., submaximal and maximal), only data as close to peak as possible were extracted for the calculation of effect sizes. Further exercise protocol information can be found in supplementary material. Positive effects are denoted by ↑ = small (> 0.2), ↑↑ = medium (> 0.5), ↑↑↑ = large (> 0.8). Negative effects are denoted by ↓ = small (< − 0.2), ↓↓ = medium (< − 0.5), ↓↓↓ = large (< − 0.8). No change is denoted by ↔ (− 0.2 to 0.2). Given that case-reports (N = 1) prohibit the calculation of an effect size, the effects of these strategies are presented using percent change between ergogenic and control

*AD* autonomic dysreflexia (‘boosting’), *CV* cardiovascular, *FES* functional electrical stimulation, *H-SCI* high-level spinal cord injury, *L-SCI* low-level spinal cord injury, *SCS* spinal cord stimulation, ${{\dot{\text{V}}}{\text{O}}}_{2}$ peak oxygen uptake, *W* watts.

‘-‘ means that an effect size could not be calculated

**Table 2:**
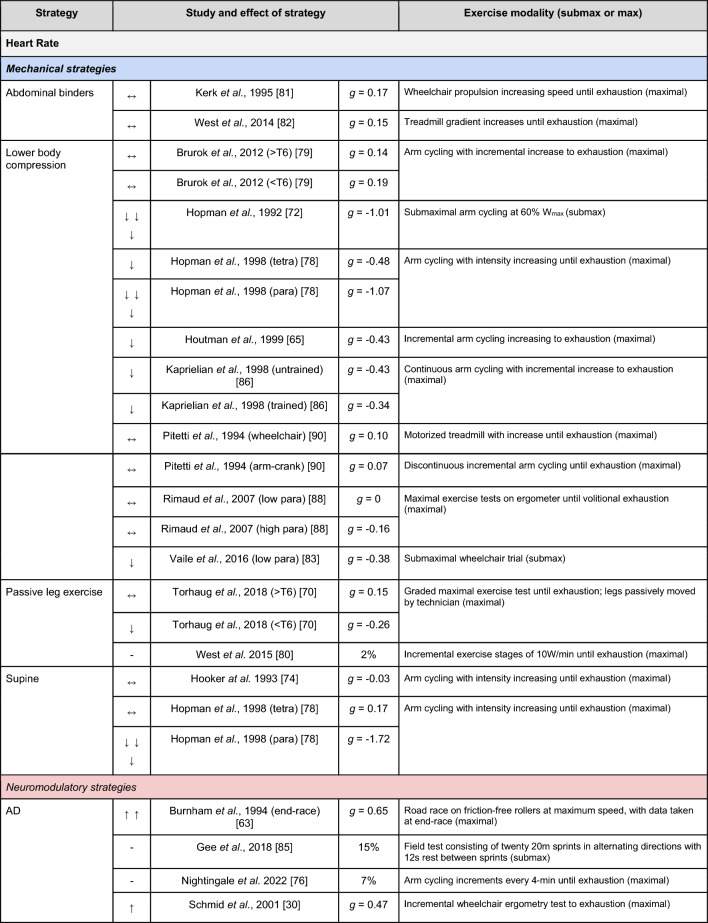

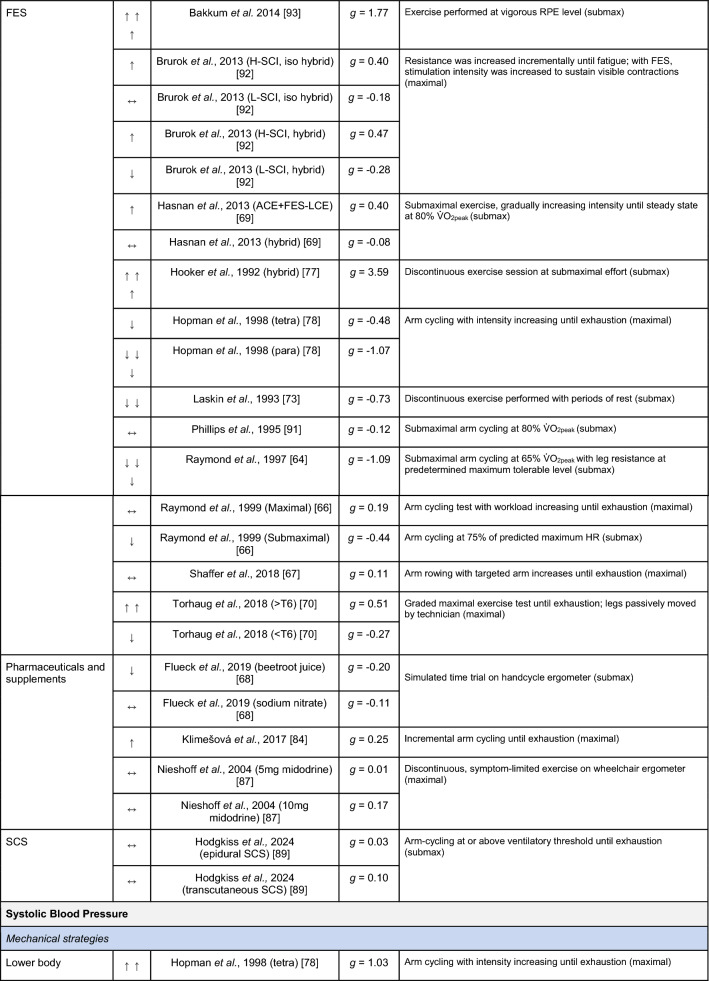

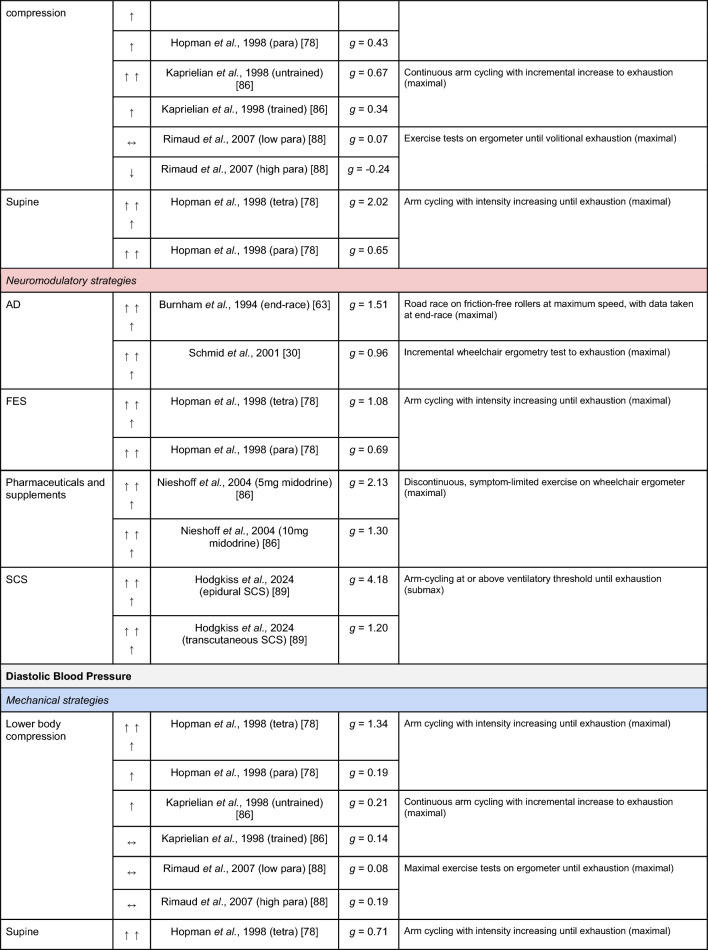

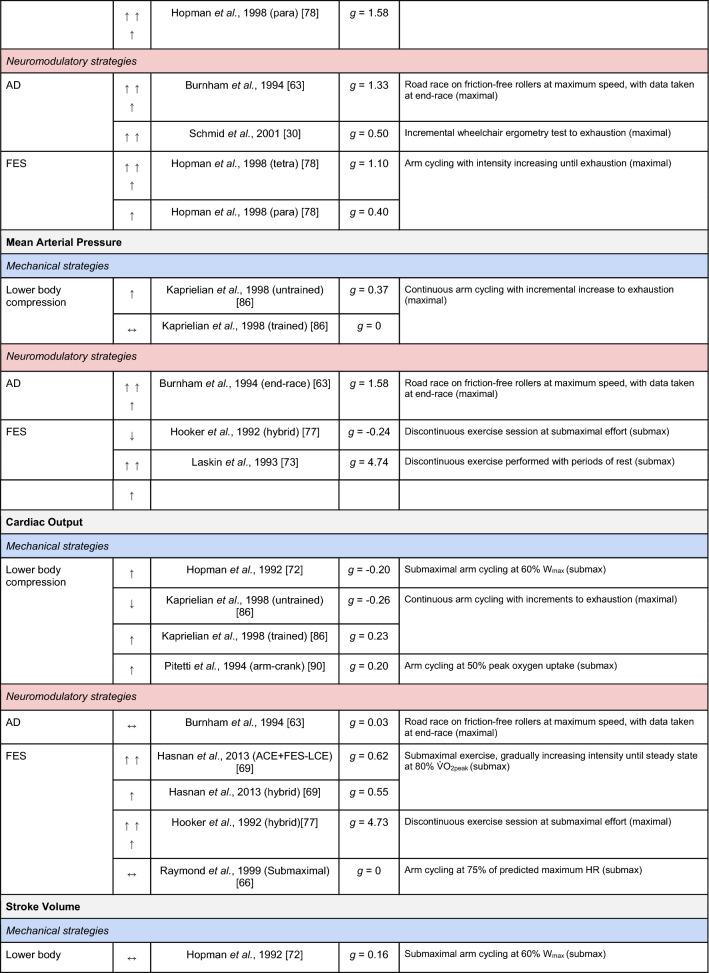

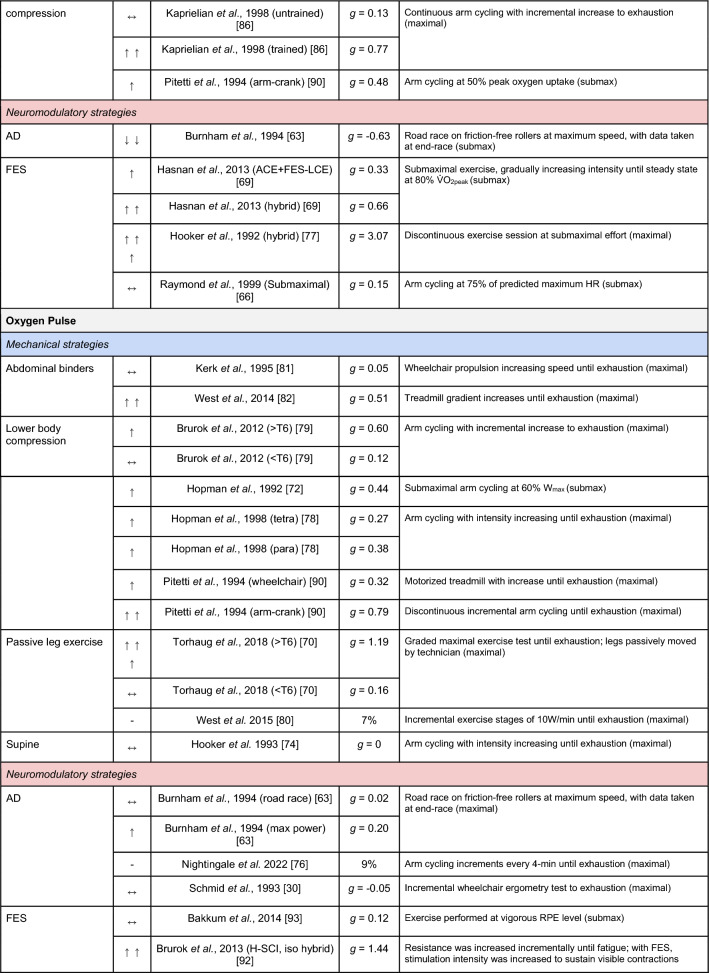

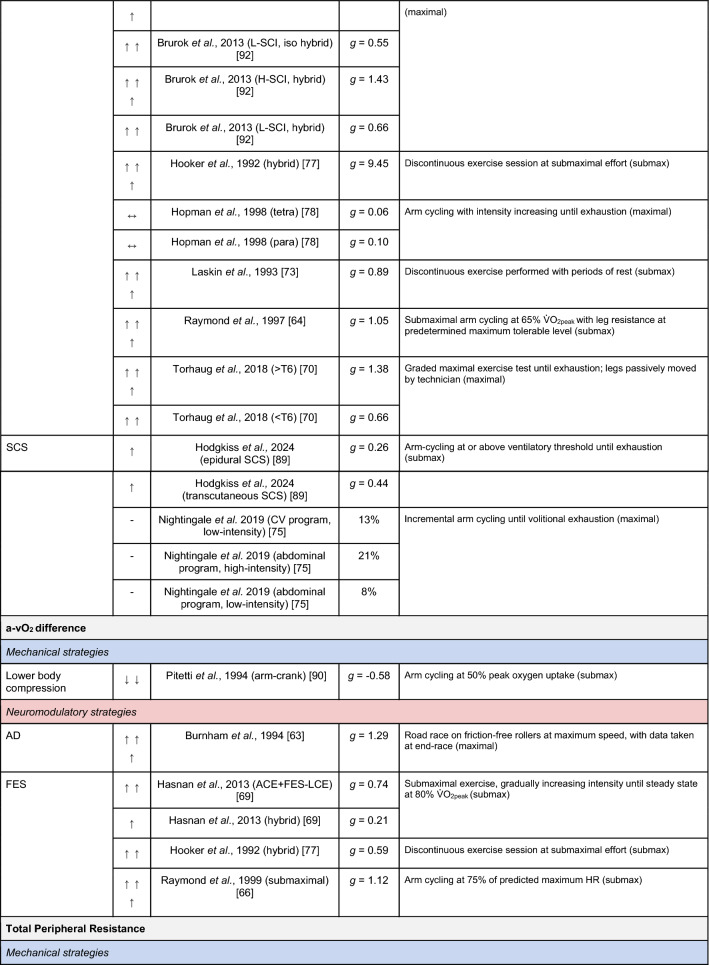

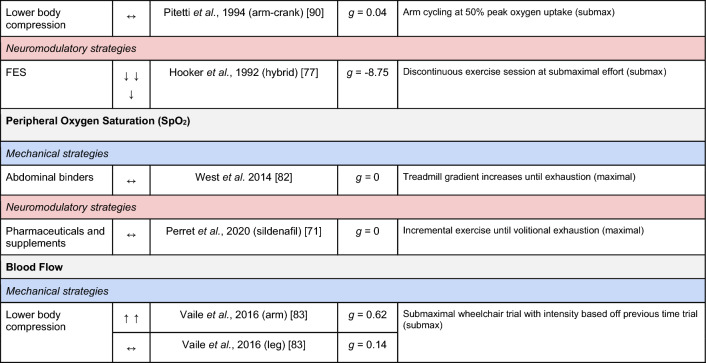
Effects of individual mechanical and neuromodulatory strategies on cardiovascular outcomes

Hedges’ *g* effect sizes are calculated for each individual study. Where studies reported outcomes for multiple exercise intensities (e.g., submaximal and maximal), only data as close to peak as possible were extracted for the calculation of effect sizes. Further exercise protocol information can be found in supplementary material. Positive effects are denoted by ↑ = small (> 0.2), ↑↑ = medium (> 0.5), ↑↑↑ = large (> 0.8). Negative effects are denoted by ↓ = small (< − 0.2), ↓↓ = medium (< − 0.5), ↓↓↓ = large (< − 0.8). No change is denoted by ↔ (− 0.2 to 0.2). Given that case-reports (N = 1) prohibit the calculation of an effect size, the effects of these strategies are presented using percent change between ergogenic and control.

*AD* autonomic dysreflexia (‘boosting’), *CV* cardiovascular, *DBP* diastolic blood pressure, *FES* functional electrical stimulation, *HR* heart rate, *H-SCI* high-level spinal cord injury, *L-SCI* low-level spinal cord injury, *MAP* mean arterial pressure, *Q* cardiac output, *SBP* systolic blood pressure, *SCS* spinal cord stimulation, *SV* stroke volume, *TPR* total peripheral resistance

The most reported performance outcomes were absolute ${{\dot{\text{V}}}{\text{O}}}_{2}$ (9/14 mechanical; 11/21 neuromodulatory strategies), relative ${{\dot{\text{V}}}{\text{O}}}_{2}$ (8/14 mechanical; 12/21 neuromodulatory strategies) and power output (9/14 mechanical; 10/20 neuromodulatory strategies). Time (e.g., total sprint time, time to exhaustion) was reported in 2/14 and 4/21 mechanical and neuromodulatory strategies, respectively.

The most reported cardiovascular outcome was HR (14/14 mechanical; 17/21 neuromodulatory strategies). Oxygen pulse was reported in 5/14 mechanical and 7/21 neuromodulatory strategies, and was calculated by authors using ${{\dot{\text{V}}}{\text{O}}}_{2}$ and HR data in 5/14 mechanical and 5/21 neuromodulatory strategies. Systolic BP (SBP) was reported in 3/14 mechanical and 5/21 neuromodulatory strategies. Cardiac output and stroke volume were reported in 3/14 mechanical and 4/21 neuromodulatory strategies. Diastolic BP and mean arterial pressure were reported in 3/14 mechanical and 3/21 neuromodulatory strategies. Arteriovenous oxygen difference was reported in 1/14 and 4/21 mechanical and neuromodulatory strategies, respectively.

Adverse events were reported in two of the mechanical strategies. Kerk et al. [81] described that two female participants expressed discomfort with the abdominal binder impinging on their breast line, which may have limited tidal volume and served as a noxious stimulus to decrease range of trunk motion and thus prevent further discomfort. Hooker et al. [74] reported one episode of AD prior to supine cycling, but once the participant had voided their bladder they performed the test without any complications. Adverse events were reported in eight of the neuromodulatory strategies. Reports of SBP exceeding 200 mmHg were found in two boosting studies [30, 63]. In particular, Burnham et al. [63] stated that two SBP measurements (pre- and post-race) in the same participant were > 200 mmHg, of which this individual indicated that he could not reliably predict when he would be in a boosted state. An FES study [92] reported that two individuals with tetraplegia (C5 and C6) exhibited symptoms of hypotension (feeling faint, nausea and pallor) during peak ACE testing but not during FES hybrid testing. One study assessing the effects of midodrine [87] stated that two participants experienced dizziness and light-headedness, but these reports were during the placebo trials. Flueck et al. [68] reported that one participant experienced gastrointestinal side-effects of beetroot ingestion, but it is uncertain whether this was a participant with SCI or a non-SCI control. One study assessing the effects of sildenafil citrate [71] reported that participants complained more frequently about side effects from sildenafil ingestion during and after exercise, relative to the placebo. These included erections, dizziness, headache and heartburn. However, as non-SCI participants were also included in this study, it cannot be determined how many symptoms were experienced by the SCI group alone. One SCS study reported that a participant (with an epidural implant) developed a skin lesion on their back due to friction with their chair but this was neither related to the exercise protocol nor receiving SCS [89].

### Effects of Strategies

**Fig. 4:**
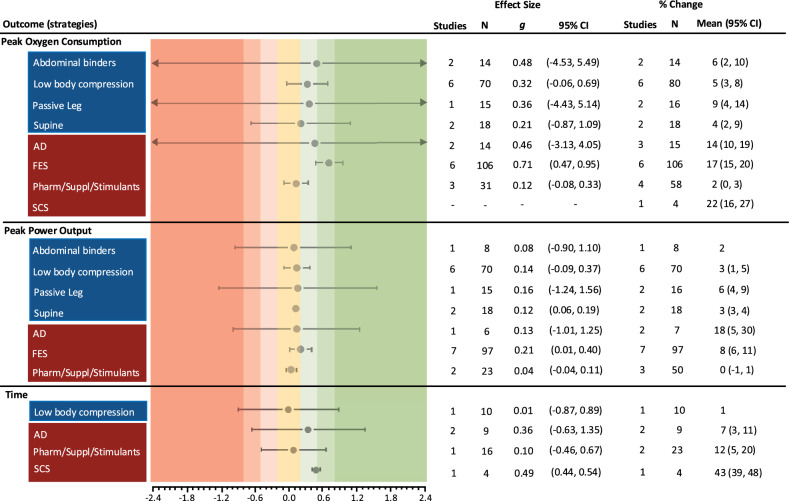
Standardised effect sizes (Hedges’* g*) with 95% confidence intervals (CI) for exercise performance outcomes, grouped by mechanical (blue) and neuromodulatory (red) ergogenic strategies. Percentage changes with 95% CIs are presented to account for case-reports and studies in which an effect size could not be calculated. N refers to the number of observations, not participants *per se*, given that some studies repeated trials within participants. *AD* autonomic dysreflexia, *FES* functional electrical stimulation, *Pharm/Suppl/Stimulants* pharmaceuticals, supplements and stimulants, *SCS* spinal cord stimulation

**Fig. 5:**
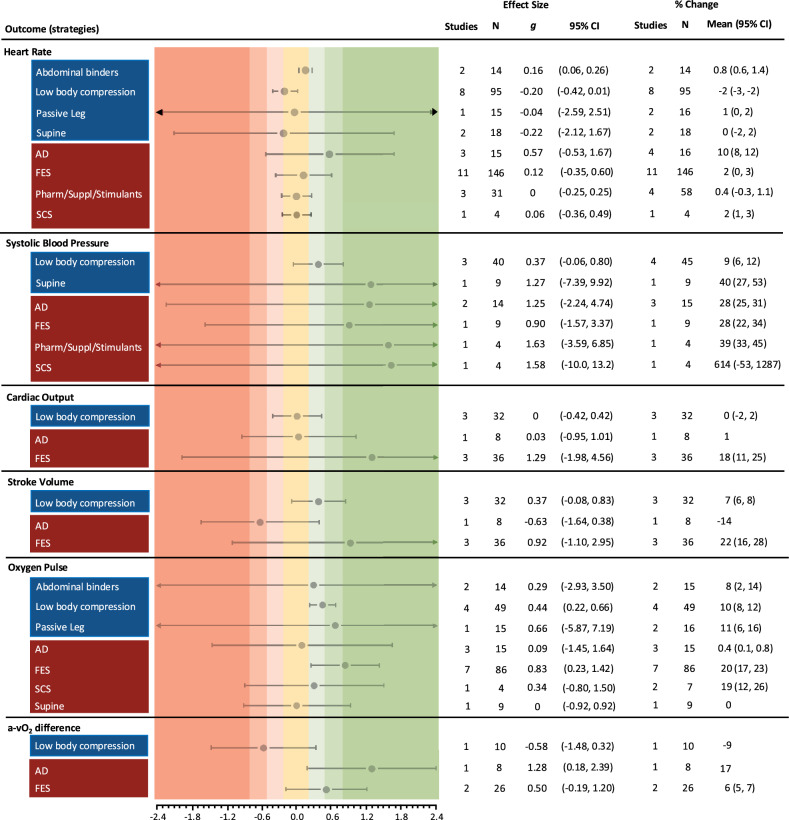
Standardised effect sizes (Hedges’ *g*) with 95% confidence intervals (CI) for cardiovascular outcomes, grouped by mechanical (blue) and neuromodulatory (red) ergogenic strategies. Only data as close to peak as possible were extracted for the calculation of cardiovascular effect sizes (i.e., where studies performed at intensities corresponding to 40, 60 and 80% ${{\dot{\text{V}}}{\text{O}}}_{{2{\text{peak}}}}$, only the outcome data for 80% ${{\dot{\text{V}}}{\text{O}}}_{{2{\text{peak}}}}$ were extracted). Percentage changes with 95% CIs are presented to account for case-reports and studies in which an effect size could not be calculated. N refers to the number of observations, not participants *per se*, given that some studies repeated trials within participants. *AD* autonomic dysreflexia, *FES* functional electrical stimulation, *N* number of observations, *Pharm/Suppl/Stimulants* pharmaceuticals, supplements and stimulants, *SCS* spinal cord stimulation

Effects of the ergogenic strategies on performance and cardiovascular outcomes are reported within Figs. 4 and 5, respectively.

On the whole, the mechanical strategies had negligible or small effects on cardiovascular outcomes, which parallels the minor changes observed in performance. Supine arm cycling had a large effect on SBP, relative to seated cycling, but this did not map onto meaningful improvements in exercise performance. Evidence for the neuromodulatory strategies was mixed, with small-to-large effects across all cardiovascular outcomes. There were greater mean changes in ${{\dot{\text{V}}}{\text{O}}}_{{2{\text{peak}}}}$ with neuromodulatory strategies, but there was little evidence to suggest that these may also augment peak power output. However, when accounting for case-reports the % change in peak power output with AD appears to be the greatest.

Overall, despite some moderate-to-large effect sizes most CIs overlap the line of no effect, indicating large variability in responses. The percentage changes in cardiovascular and performance outcomes were mainly greater with the neuromodulatory strategies and therefore appear more robust than the mechanical strategies. Notably, SCS had the largest % change in ${{\dot{\text{V}}}{\text{O}}}_{{2{\text{peak}}}}$ across all strategies, but this was only reported in an N-of-1 study. Likewise, the % change for performance time was greatest with SCS, but this was only reported in one study with four participants.

## Discussion

This systematic scoping review examined various strategies to augment the cardiovascular system and acutely enhance exercise performance in individuals with SCI. We described and synthesised the specific cardiovascular and performance outcomes reported for each ergogenic strategy type. Strategies were identified to be either mechanical (i.e., strategies that aim to prevent blood pooling in the lower limbs and abdomen by the mobilisation of the lower limbs or application of an external aid) or neuromodulatory (i.e., strategies that aim to act on the nervous system, in order to elicit a normalised sympathetic response to exercise). Finally, we calculated effect sizes for individual cardiovascular and performance outcomes to quantify the effects of each strategy.

### Mechanical Strategies

Abdominal binders were first reported to improve resting haemodynamics in the clinical setting in the 1980s, when an inflated cuff was used to increase abdominal pressure and prevent orthostatic hypotension [94]. Since then, studies have suggested that this strategy can facilitate venous return [95] and increase resting left-ventricular stroke volume and cardiac output by 15% and 28%, respectively, in individuals with SCI [96]. In the current review, the evidence on its effectiveness for enhancing exercise performance was mixed. While one study observed moderate and large improvements in oxygen pulse and ${{\dot{\text{V}}}{\text{O}}}_{{2{\text{peak}}}}$, respectively [82], another study showed no change [81]. Elsewhere, abdominal binders have elicited improvements in field-based assessments of exercise performance, including acceleration/deceleration time and distance covered during a repeated 4-min wheelchair push test [97]. One suggested mechanism for an improved exercise performance is due to enhanced configuration of the respiratory muscle pump and reduced vascular compliance preventing abdominal blood pooling [82]. Others have noted improvements in respiratory function with abdominal binders [96, 98], which may also contribute to greater performance benefits. Notably, both abdominal binding studies included in this review recruited young national-level athletes with chronic SCI. Future research could consider whether abdominal binders may be effective in older or sedentary individuals with SCI.

Lower-body compression, implemented using socks/stockings or occlusion cuffs, are designed to prevent blood pooling in the lower extremities, facilitate venous return and augment stroke volume. Likewise, lower-body positive pressure systems and anti-gravity suits are designed to provide a “milking action on the lower limbs” to mimic the skeletal muscle pump and thus increase venous return and stroke volume [65, 86]. For comparison, lower-body negative pressure is shown to reduce stroke volume [99]. Compression stockings seemingly have no effect on ${{\dot{\text{V}}}{\text{O}}}_{{2{\text{peak}}}}$ [88], peak power output [88], or maximal sprint time [83], and evidence on the effectiveness of lower-body pressure via occlusion cuffs or bladders is also inconclusive. Kaprielian et al. [86] showed that occlusion bladders inflated between the ankle and the groin area (between 55 and 60 mmHg) did not improve ${{\dot{\text{V}}}{\text{O}}}_{{2{\text{peak}}}}$ or peak power output in either trained or untrained individuals with lower-level paraplegia, and did not significantly alter resting cardiovascular outcomes. On the other hand, Brurok et al. [79] inflated occlusion cuffs on the thighs to 100 mmHg above resting SBP and observed significant improvements in ${{\dot{\text{V}}}{\text{O}}}_{{2{\text{peak}}}}$, peak power output and oxygen pulse in individuals with high- (> T6) but not low- (< T6) level SCI. Therefore, these effects may only be present in individuals with impaired cardiovascular function, or there may be a need for a greater occlusion pressure to observe meaningful changes. With regards to anti-gravity suits, one study with fluctuating pressure (50–75 mmHg) demonstrated an increase in ${{\dot{\text{V}}}{\text{O}}}_{{2{\text{peak}}}}$ during graded arm-crank and wheelchair tests, with accompanying improvements in submaximal mean arterial pressure and stroke volume [90]. However, two studies showed no change in ${{\dot{\text{V}}}{\text{O}}}_{{2{\text{peak}}}}$ (continuous pressure of 52–55 mmHg) despite a higher resting BP and stroke volume [72, 78], and one study even showed a negative effect on ${{\dot{\text{V}}}{\text{O}}}_{{2{\text{peak}}}}$ (pulsating pressure of 35–70 mmHg) with no changes in peak power output or HR either [65]. This latter study was performed on individuals with low-level paraplegia and relatively intact sympathetic control (HR_peak_ ~ 172–175 bpm). Ultimately, differences in the pressure applied on the lower limbs across studies, and whether this is static/continuous or pulsating, or is applied in individuals with disrupted or stable supraspinal sympathetic control, may explain the inconsistent findings on exercise performance. Future work could look to quantify the degree of compression required to yield meaningful cardiovascular and thus acute performance benefits. Furthermore, research should look to confirm whether only those with impaired cardiovascular function are likely to experience any benefits.

A recent study by Soriano et al. [100] observed that passive leg cycling augmented SBP, HR, stroke volume and cardiac output. However, this study did not include a control comparison. In the current review, one study split participants into high- (> T6) and low-level (< T6) SCI but observed no alterations for either group in cardiovascular responses, nor improvements in ${{\dot{\text{V}}}{\text{O}}}_{{2{\text{peak}}}}$ or peak power output, relative to an arm-crank only control comparison [70]. In contrast, a case study of a retired world-class wheelchair basketball athlete with a T3-level SCI demonstrated that active arm cycling combined with passive leg cycling elicited a greater ${{\dot{\text{V}}}{\text{O}}}_{{2{\text{peak}}}}$ and peak power output in comparison to arm cycling alone, with a ~ 7% higher peak oxygen pulse [80]. Other studies on healthy males with tetraplegia and paraplegia have utilised passive leg exercise as a control strategy and noted that it elicits a lower submaximal ${{\dot{\text{V}}}{\text{O}}}_{2}$, arterio-venous oxygen extraction and HR at the same corresponding fixed workload, in comparison to FES cycling, but induces a similar left-ventricular stroke volume [101, 102]. Evidently, further work is required to ascertain whether combining a lower-body passive form of exercise (without FES) with volitional, upper-body exercise is beneficial for driving cardiovascular responses and enhancing acute exercise performance in comparison to more widely researched strategies such as FES [93].

Two studies investigated the effects of supine exercise relative to an upright, seated position [74, 78]. The rationale behind this strategy focuses on alleviating the effects of gravity by preventing blood pooling in the legs, promoting venous return and an increase in central blood volume to augment stroke volume and thus cardiac output [103]. Hooker et al. [74] reported no improvements in ${{\dot{\text{V}}}{\text{O}}}_{{2{\text{peak}}}}$, peak power output or peak HR in individuals with upper-thoracic SCI (T1–T5). However, Hopman et al. [78] observed that individuals with tetraplegia, but not paraplegia, had a significantly greater ${{\dot{\text{V}}}{\text{O}}}_{{2{\text{peak}}}}$ with supine exercise. Unfortunately, this particular paper appears to be relatively underpowered given the lack of other statistically significant findings despite several large mean differences. For example, the percentage changes in SBP with supine exercise, as compared to seated, were 53% and 26% for individuals with tetraplegia and paraplegia, respectively, suggesting that a greater blood volume shift from the periphery likely contributes to performance changes. Notably, one study investigating the effects of supine exercise was excluded during screening as a number of participants concurrently wore compression stockings and/or binders during supine exercise and therefore the effects of the supine strategy alone could not be isolated [103]. This study reported a significantly greater ${{\dot{\text{V}}}{\text{O}}}_{{2{\text{peak}}}}$ and peak power output in both high- (C7 and above) and low- (below C7) SCI groups. Alongside any cardiovascular alterations, it could be hypothesised that lying supine may also contribute to a greater trunk stability to support performance. Given the postural instability typically exhibited in the seated position [104], exercising whilst supine may remove the need for participants to compensate for trunk impairments. However, more research is required to explore these hypotheses.

Whilst not included in this review, as the effects of each individual strategy could not be isolated, Hopman et al. [78] combined compression stockings with abdominal binders during arm-crank ergometry. Yet, this seemingly had no additive effect on exercise performance nor cardiovascular outcomes in individuals with paraplegia or tetraplegia. This suggests there may be a need to activate the sympathetic nervous system directly or restore the natural internal skeletal muscle pump mechanics, as opposed to via external methods, to achieve meaningful performance benefits. This also highlights that the evidence base for comparing single versus combined strategies is limited. As there are multiple aetiologies for cardiovascular impairments in the SCI population, combining interventions may have a synergistic effect that may not be captured in isolated strategies and should be an avenue of future research.

### Neuromodulatory Strategies

AD is a potentially life-threatening condition triggered by noxious or non-noxious stimuli and is characterised by a sudden increase in sympathetic vasoconstriction below the lesion level [105]. This vasoconstriction leads to an increase in SBP that can exceed 200 mmHg [106], and can have severe consequences including seizures, arrhythmias, cerebral haemorrhage, and myocardial infarction [106]. Given that athletes with higher neurological levels of injury are at a physiological disadvantage during training and competition, owing to a disrupted sympathetic response to exercise, it is not uncommon for such individuals to intentionally induce AD to obtain a beneficial exercise pressor response and enhance performance [107]. This may be induced by clamping the urinary catheter to produce bladder distension, excessive tightening of leg straps, twisting and/or sitting on the scrotum, or even via self-inflicting injury below the SCI [108]. This practice is known as ‘boosting’ and is prohibited by the International Paralympic Committee [109]. Research on athletes with SCI has demonstrated that boosting can increase ${{\dot{\text{V}}}{\text{O}}}_{{2{\text{peak}}}}$ [30, 63], peak power output [30], and time-trial performance [63]. These enhancements are accompanied by increases in BP, HR, circulating catecholamines, and a greater arterio-venous oxygen extraction [29, 30, 63]. Whilst there are typical symptoms indicative of AD (e.g., profuse sweating, goosebumps, red-flushed skin, severe headache), it is important to note that some individuals can be asymptomatic and be completely unaware that they are in a boosted state. Burnham et al. [63] reported that five out of six individuals who intentionally induced AD via bladder overdistension could not reliably predict when they would be boosting and could not stop at their own discretion. Indeed, cases of accidental/unintentional boosting have also been reported in the literature [76, 85]. Unintentional boosting in an elite wheelchair rugby athlete was shown to improve repeated sprint performance in a field-based test, whilst elevating HR and lowering ratings of perceived exertion (RPE) [85]. Another case-report in an untrained individual with tetraplegia observed that a change in SBP of 58 mmHg resulted in 29% and 24% improvements in ${{\dot{\text{V}}}{\text{O}}}_{{2{\text{peak}}}}$ and peak power output, respectively [76].

FES has traditionally been utilised for locomotor recovery but has also been shown to be effective at improving aerobic capacity following exercise interventions in individuals with SCI [110]. While categorised as a neuromodulatory intervention herein, FES also operates mechanically by promoting lower-limb movement to activate the skeletal muscle pump and facilitate venous return [69, 111]. Overall, the summary effect sizes indicate robust improvements in ${{\dot{\text{V}}}{\text{O}}}_{{2{\text{peak}}}}$, but not peak power output, with the addition of lower-body FES (i.e., the ergogenic strategy) to upper-body exercise (i.e., the control strategy). This finding is perhaps unsurprising given the expected increase in oxygen consumption requirements with activation of a larger skeletal muscle mass. However, while there is a range of small-to-large effect sizes across cardiovascular outcomes, the large variability makes it difficult to draw conclusions on the exact mechanisms underpinning the changes in performance. Studies included in this review, and elsewhere, have reported alterations in central haemodynamics during FES including increases in indices of left-ventricular stroke volume [64, 66, 70, 77, 92, 112], whilst others have shown no change [69, 93, 101, 102]. Evidence on the effect of FES at improving tissue oxygen extraction is also mixed, with studies reporting an increase [66, 113] or no change [69, 77]. The lack of consistent findings across studies is likely owing to the range of FES modalities reported in the literature, including static lower-body NMES or FES-induced cycling paired with upper-body exercise, and hybrid FES (e.g., rowing), whereby FES engages lower-body musculature with upper-body movements in a more coordinated manner. Notably, however, a study by Brurok et al. [92] found no differences in performance between static lower-body NMES and hybrid FES cycling strategies, in comparison to arm cycling only. Studies also may not have included a preconditioning, or accommodation, period prior to testing. These ‘lead in’ sessions are often necessary to familiarise participants to receiving FES so that atrophied lower limb muscles do not fatigue too quickly and may explain the variable responses [114]. Nonetheless, the range of FES parameters reported across studies, including pulse amplitude, widths and frequencies, makes it challenging to determine the optimal settings for augmenting the cardiovascular system and enhancing exercise performance. It should be noted that there are suggestions of FES potentially inducing AD, in studies included in this review [92] and elsewhere [112, 115], which may therefore be clouding the effects of FES alone.

This review included several studies investigating the effects of pharmaceuticals, supplements or stimulants on exercise performance. Overall, the summary effect sizes for ${{\dot{\text{V}}}{\text{O}}}_{{2{\text{peak}}}}$ and peak power output were negligible, but there was evidence of a mean 12% improvement in performance time across two studies [68, 84]. While there were no overall effects on HR there was a very large effect on SBP, but this was only measured in one study. Caffeine is a well-known stimulant of the central nervous system, with evidence indicating that 3–6 mg/kg dosage prior to exercise may improve aerobic exercise performance in non-SCI individuals [116–118], but the effects are highly variable. Klimešová et al. [84] observed that 3 mg/kg caffeine ingestion prior to exercise had no impact on ${{\dot{\text{V}}}{\text{O}}}_{{2{\text{peak}}}}$, peak power output, HR or RPE in individuals with SCI. Of note, a study in the non-SCI population demonstrated that 4 mg/kg caffeine ingestion improved cycling, but not handcycling, time-trial performance [119], suggesting that the effects of caffeine may be limited by the amount of active musculature engaged during exercise. Midodrine is an alpha-sympathomimetic agent typically administered to treat hypotension, with demonstrated efficacy for mitigating hypotension in individuals with SCI [120, 121]. Nieshoff et al. [87] observed that ${{\dot{\text{V}}}{\text{O}}}_{{2{\text{peak}}}}$ and SBP increased more with a 10 mg dose in comparison to 5 mg, but responses were variable across all four participants. Given there are reports that midodrine can worsen BP instability and result in unwanted side-effects [122, 123], further work is necessary to conclude whether this strategy is safe and could be used as an aid to support acute exercise performance or rehabilitation in the clinical setting. Beetroot juice and/or sodium nitrate had no benefits on time-trial performance, submaximal ${{\dot{\text{V}}}{\text{O}}}_{2}$ or power output, nor resting BP, in national level Para cyclists [68]. It is important to note that these supplements are likely not activating the sympathetic circuitry directly, but still theorised to impact cardiovascular mechanisms (e.g., via increased nitric oxide availability and thus improved vascular function). In non-SCI individuals, nitrate supplementation has been shown to improve exercise performance through other mechanisms such as by influencing vascular tone (i.e., nitric oxide-mediated vasodilation), oxygen transport, mitochondrial efficiency and/or glucose uptake [124–127]. The largest study on supplements in this review investigated the use of sildenafil citrate on exercise performance in 27 athletes with SCI [71]. Relative to a placebo, ${{\dot{\text{V}}}{\text{O}}}_{{2{\text{peak}}}}$ was unchanged and peak power output decreased with sildenafil citrate ingestion, with adverse side effects also reported. It was concluded that benefits at a submaximal exercise intensity would also be unlikely and therefore this strategy is not recommended for use by individuals with SCI.

Electrical SCS is rapidly emerging as a promising strategy to restore various neurological functions following SCI. While originally designed to treat chronic pain over 50 years ago [128], there has been an explosion in the literature over the last decade with studies revealing the beneficial effects of epidural SCS on both motor and autonomic functions [129–133]. Essentially, epidural SCS consists of a surgically implanted electrode array on top of the dura, connected to a subcutaneous abdominal pulse generator, that provides direct electrical stimulation to the spinal cord. By activating the sympathetic circuitry below the spinal lesion, a number of studies have reported improvements in cardiovascular function at rest [15, 18, 19, 134]. With regards to exercise, a case report by Nightingale et al. [75] compared the effects of four different SCS programs on upper-body exercise performance in a middle-aged, untrained male with chronic, motor-complete tetraplegia. Specific electrode configurations (i.e., selection of specific anodes and cathodes on the array) and stimulation parameters (i.e., frequency, pulse width and amplitude) were selected for two abdominal and two cardiovascular programs. The abdominal programs were designed to target trunk muscle activation only, whereas the cardiovascular programs were designed to activate the trunk and lower-limb musculature. Each of these programs were then administered at a low- (below motor threshold) and high-intensity (highest tolerable intensity) stimulation amplitude. In comparison to a control condition without epidural SCS, all four programs improved ${{\dot{\text{V}}}{\text{O}}}_{{2{\text{peak}}}}$ by 15–26% during a graded arm-crank test to exhaustion, which was accompanied by an 8–21% increase in peak oxygen pulse. The greatest improvement in ${{\dot{\text{V}}}{\text{O}}}_{{2{\text{peak}}}}$ was observed with the low-intensity cardiovascular program, which augmented mean arterial pressure by 15 mmHg and was independent of trunk and lower-limb muscle activation. This suggests that epidural SCS can restore supraspinal vasomotor control, to increase venous return and stroke volume (and thus increase cardiac output), and therefore acutely enhance exercise performance. Notably, the 26% improvement in ${{\dot{\text{V}}}{\text{O}}}_{{2{\text{peak}}}}$ in this study exceeds the percentage changes calculated across all other strategies in this review. While this holds significant promise for individuals with SCI interested in using this strategy for performance benefits, further work is required to corroborate these findings. Furthermore, epidural SCS is constrained by its invasive and expensive surgical procedure, which limits its accessibility for the majority of the SCI population.

On the other hand, transcutaneous SCS is a non-invasive form of SCS consisting of electrodes placed on the skin surface, which may offer a cheaper and more accessible strategy in comparison to epidural SCS. Several studies have demonstrated that transcutaneous SCS can activate the same neural structures as epidural SCS, specifically the dorsal root afferents [135–137], and remarkable improvements in cardiovascular autonomic function have been reported [17, 138]. Data from a case-series [89] reveals similar modulations in cardiovascular function (i.e., BP, predicted left-ventricular cardiac contractility, total peripheral resistance) with both epidural and transcutaneous SCS at rest in individuals with motor-complete SCI above T6. These responses map onto improvements in upper-body time to exhaustion for exercise performed at the same corresponding submaximal workloads. Specifically, individuals could exercise for up to 19 min longer [average 48% (range: 32 - 80%) improvement] with cardiovascular-optimised epidural or transcutaneous SCS, relative to sham conditions. These findings highlight the outstanding potential for non-invasive transcutaneous SCS to be utilised as an ergogenic aid, perhaps not only during exercise rehabilitation or elite performance, but also for simply lessening symptoms of fatigue during activities of daily living. Accordingly, a number of ongoing clinical trials (ISRCTN17856698; NCT05960448; NCT06313515) are investigating the acute ergogenic effects of transcutaneous SCS, as well as potential long-term therapeutic benefits that may occur following an exercise intervention paired with stimulation [139].

### Considerations, Applications and Future Directions

Across several studies, the effects of ergogenic aids in individuals with higher neurological levels of injury (i.e., cervical or upper-thoracic) may have been clouded by the inclusion of participants with lower-thoracic and/or lumbar SCI. Whilst a few studies attempted to account for impaired supraspinal sympathetic control by dichotomising cohorts into above and below T6, it is possible that not all participants with SCI > T6 were also autonomically complete. As there were a limited number of studies that did this, we were unable to report a summary on the effects of each strategy on individuals with SCI above and below T6. Notably, recent studies have demonstrated that alterations in cardiovascular control cannot be determined by neurological level of injury and injury severity derived from a motor-sensory exam [13, 140]. Consequently, researchers should incorporate a comprehensive autonomic test battery [141] to determine an individual’s degree of autonomic dysfunction prior to exercise testing, and should endeavour to compare the effects of ergogenic strategies on those with neurological levels of SCI above and below T6.

A recent opinion piece has touted acute intermittent hypoxia (AIH) as a plausible alternative strategy to re-engage dormant sublesional sympathetic circuits following SCI [142]. This strategy has demonstrated a restoration of sympathetic cardiovascular control in experimental animal models [143], but does not augment HR or arterial BP 30-min post-treatment in individuals with SCI [144]. Whether AIH could be used as a ‘prehab’ strategy to prime the sympathetic nervous system for exercise remains uncertain and warrants further investigation.

Ultimately, studies should continue to confirm the safety, efficacy and practicality of utilising the ergogenic aids discussed in this review outside of lab-based settings to discover whether they are appropriate for supporting clinical or community-based exercise, or elite athletic performance. Pairing longitudinal exercise interventions with ergogenic aids may result in chronic, therapeutic adaptations, as has been shown in FES research [110]. This is particularly important given that individuals with tetraplegia specifically may not achieve meaningful benefits following an exercise training programme [145] as a result of reduced cardiovascular responses to exercise. Furthermore, older adults with SCI experience reduced exercise intervention-related cardiorespiratory fitness benefits compared to younger adults [50], thus utilising ergogenic aids could mitigate these reductions. Finally, there are a few reports of combining strategies during exercise (e.g., compression stockings and abdominal binders) [78, 103], and a combined mechanical and neuromodulatory strategy such as abdominal FES has also shown significant promise [146]. Whether other combinations (e.g., AIH and SCS) could lead to enhanced acute performance benefits should be explored.

Whilst the purpose of this review was to identify ergogenic aids that augment the cardiovascular system to enhance acute exercise performance, we acknowledge that the level of injury affects systems other than the cardiovascular system and may subsequently influence aerobic exercise capacity through these systems. For example, individuals with higher level injuries often exhibit impaired pulmonary function due to the loss of neural drive to respiratory muscles [147]. As the cardiovascular and respiratory systems are intricately linked, altered pulmonary function (e.g., respiratory muscle weakness, dynamic hyperinflation) likely influence blood flow and cardiac function and, subsequently, aerobic exercise capacity [23, 148]. Research on strategies that targets both cardiovascular and respiratory impairments during exercise in individuals with SCI would be noteworthy.

Finally, several other strategies were identified during screening that, while not designed to target the cardiovascular system, were investigated with regards to exercise capacity and/or performance, including creatine [149, 150], non-invasive ventilation [151, 152], and lower-body negative pressure [99].

### Limitations of the Included Studies

Relatively small sample sizes across studies (mean n: 9, range 1–27), with participants often further dichotomised into groups of paraplegic and tetraplegic individuals, or above and below T6, made it difficult for conclusive recommendations to be made on which ergogenic aids should be used to support exercise performance without further adequately powered studies. Furthermore, as sex-based differences in cardiorespiratory responses to exercise have been noted in the non-SCI population [153], future studies should examine these differences in the context of SCI. Recent work in SCI [154] and the non-SCI population [155–158] has discussed the lack of female recruitment and has suggested that it is simply no longer acceptable to exclude female participants without valid justification. Whilst it is acknowledged that a greater number of young adult males live with SCI than young females [159], there is a concerning difference in the number of male and female participants in the studies included herein. Across 32 studies, 94% of participants were male and the highest number of females included within a single study was a mere four [81]. Additionally, future studies should also strive for more inclusive study designs with respect to age, neurological level of injury and severity to further improve the generalisability of their findings.

Secondly, data replication across studies made it difficult for decisions to be made on study inclusion. For example, studies on leg vascular occlusion [79] and FES hybrid cycling [92] both report the same data for the ACE control and FES hybrid cycling strategies. These studies also raised concerns given that ${{\dot{\text{V}}}{\text{O}}}_{2}$ and HR were identical but oxygen pulse was different despite the calculation for oxygen pulse being ${{\dot{\text{V}}}{\text{O}}}_{2}$/HR. Other studies report almost identical protocols and/or participant demographics [69, 88, 91, 113, 160, 161], but do not acknowledge the preceding paper. Transparent reporting is therefore encouraged to enable readers to determine whether there is a duplication of participants across studies.

Thirdly, some studies did not randomise the order of strategies, or strategies were combined within a single-session, which may have resulted in an additive or residual effect of the preceding strategy. Several articles were also excluded for not including a discrete control comparison (i.e., investigating FES leg cycling alone), but these studies were useful in informing the discussion. Finally, reporting on adverse events was poor overall. Authors are encouraged to be transparent going forward and report on all (including no) adverse events to enable practitioners to identify which ergogenic strategies are most likely to be safe for this population.

### Limitations of the Review

This systematic scoping review is not without its limitations. Firstly, studies that were not published in English were excluded and thereby introduces a source of language bias. However, only 1 out of 84 full texts were excluded as a result of being unavailable in English and it is therefore unlikely that this will have influenced our findings. Secondly, we did not include unpublished studies from the grey literature (i.e., Google Scholar, conference abstracts, theses etc.). Thus, there may be novel, unidentified strategies that have been explored but not reported herein. Finally, given the relatively small number of strategies, sample sizes, and substantial heterogeneity across studies, in keeping with Cochrane guidance we were unable to categorise our analysis as a meta-analysis per se. However, we still calculated study-level effect sizes, and subsequently pooled these, to provide a rudimentary insight into the efficacy of each ergogenic strategy. These findings should be considered with caution given the inherent limitations with pooling data with small sample sizes.

## Conclusion

This scoping review systematically synthesised articles exploring the use of ergogenic aids for augmenting the cardiovascular system and acutely enhancing exercise performance in individuals with SCI. Despite the growing body of literature, it is difficult to make strong recommendations on the most optimal strategy (or strategies) owing to considerable heterogeneity across study designs and a lack of adequately powered studies. Activating the sympathetic circuitry below the SCI with neuromodulatory approaches such as SCS is a novel area of investigation; further work that explores the modulation of cardiovascular control with SCS, and in particular non-invasive transcutaneous SCS, could enable a safe, optimal, and widely accessible strategy for reliably supporting exercise rehabilitation and/or competitive exercise performance for individuals with SCI.

## Supplementary Information

Supplementary Material 1.

Supplementary Material 2.

## Data Availability

All data collated during this study are included in the published article or supplementary material.

## Abbreviations

ACE: Arm crank ergometry
AD: Autonomic dysreflexia
AIH: Acute intermittent hypoxia
ANS: Autonomic nervous system
BP: Blood pressure
CI: Confidence interval
FES: Functional electrical stimulation
HR: Heart rate
NMES: Neuromuscular electrical stimulation
RPE: Rating of perceived exertion
SBP: Systolic blood pressure
SCI: Spinal cord injury
SCS: Spinal cord stimulation
SD: Standard deviation
SPNs: Sympathetic pre-ganglionic neurons
TSI: Time since injury
$${{\dot{\text{V}}}{\text{O}}}_{2}$$: Oxygen uptake
${{\dot{\text{V}}}{\text{O}}}_{{2{\text{peak}}}}$: Peak oxygen uptake
