# Supplementary material for: Strategies to Augment the Cardiovascular System and Acutely Enhance Exercise Performance in Individuals with Spinal Cord Injury: A Systematic Scoping Review
Source: Sports Med Open. 2025 Nov 6;11:125. doi: 10.1186/s40798-025-00909-7 (PMC12592629; doi:10.1186/s40798-025-00909-7)
Supplement: Supplementary file 1 — Supplementary Material 1. [file 40798_2025_909_MOESM1_ESM.docx]

**Supplementary Material S1 – S2**

**Strategies to augment the cardiovascular system and acutely enhance exercise performance in individuals with spinal cord injury: A systematic scoping review**

**Sports Medicine Open**

Hodgkiss, D.D ^1^, Balthazaar, S.J.T ^1,2,3^, Gee, C.M^2,4^, Chiou, SY^1^, Lucas, S.J.E^1,5^, Nightingale, T.E^1,2^

**^1^** School of Sport, Exercise and Rehabilitation Sciences, University of Birmingham, UK;

**^2^** International Collaboration on Repair Discoveries (ICORD), University of British Columbia, Vancouver, British Columbia, Canada. **^3^** Department of Cardiology, University Hospitals Birmingham National Health Service (NHS) Foundation Trust, Birmingham, UK; **^4^** Department of Orthopaedics, University of British Columbia, Vancouver, BC, Canada; ^5;^ Centre for Human Brain Health, University of Birmingham, United Kingdom.

**Corresponding author:** Tom E. Nightingale PhD, [T.E.Nightingale@bham.ac.uk](mailto:T.E.Nightingale@bham.ac.uk)

**Supplementary Table S1.** MEDLINE and EMBASE search strategy and results

| **#** | **Searches** | **Results** |
| --- | --- | --- |
| TERM 1: Search keywords for spinal cord injury | | |
| 1 | tetrapleg*.af. | 12,496 |
| 2 | parapleg*.af. | 73,577 |
| 3 | quadripleg*.af. | 35,350 |
| 4 | spinal cord injur*.af. | 157,995 |
| 5 | spinal cord lesion*.af. | 14,351 |
| 6 | spinal cord transection*.af. | 3,566 |
| 7 | spinal cord impair*.af. | 305 |
| 8 | spinal injur*.af. | 31,100 |
| 9 | spinal lesion*.af. | 5,547 |
| 10 | spinal transection*.af. | 1,696 |
| 11 | spinal impairm*.af. | 76 |
| 12 | spinal paraly*.af. | 645 |
| 13 | 1 or 2 or 3 or 4 or 5 or 6 or 7 or 8 or 9 or 10 or 11 or 12 | 268,445 |
| TERM 2: Search keywords for exercise/performance | | |
| 14 | exercis*.af. | 1,347,317 |
| 15 | aerobic exercis*.af. | 48,797 |
| 16 | physical activit*.af. | 546,323 |
| 17 | sport*.af. | 785,428 |
| 18 | paralympi*af. | 3,603 |
| 19 | para sport*.af. | 442 |
| 20 | adaptive sport*.af. | 341 |
| 21 | endurance exercis*.af. | 13,290 |
| 22 | power output*.af. | 23,237 |
| 23 | cardiorespiratory fitness.af. | 24,603 |
| 24 | oxygen uptake.af. | 60,108 |
| 25 | oxygen consumption*.af. | 272,136 |
| 26 | functional capacity.af. | 47,393 |
| 27 | aerobic capacity.af. | 25,459 |
| 28 | performance.af. | 4,016,537 |
| 29 | enhanc*.af. | 19,688,519 |
| 30 | ergogenic.af. | 6,144 |
| 31 | time trial.af. | 5,283 |
| 32 | time-trial.af. | 5,283 |
| 33 | time to fatigue.af. | 1,889 |
| 34 | time to exhaustion.af. | 4,677 |
| 35 | sprint*.af. | 29,958 |
| 36 | simulated match play.af. | 81 |
| 37 | simulated game play.af. | 4 |
| 38 | race.af. | 479,231 |
| 39 | 14 or 15 or 16 or 17 or 18 or 19 or 20 or 21 or 22 or 23 or 24 or 25 or 26 or 27 or 28 or 29 or 30 or 31 or 32 or 33 or 34 or 35 or 36 or 37 or 38 | 23,849,977 |
| TERM 3: Search key words related to the cardiovascular system | | |
| 40 | cardiovascul*.af. | 4,662,379 |
| 41 | heart rate*.af. | 684,801 |
| 42 | blood pressure*.af. | 1,323,746 |
| 43 | stroke volume*.af. | 117,152 |
| 44 | venous return.af. | 15,462 |
| 45 | cardiac.af. | 2,500,896 |
| 46 | blood flow*.af. | 688,316 |
| 47 | haemodynamic*.af. | 89,215 |
| 48 | hemodynamic*.af. | 654,182 |
| 49 | vascula*.af. | 2,953,234 |
| 50 | oxygen extraction.af. | 9,662 |
| 51 | arteriovenous.af. | 154,945 |
| 52 | 40 or 41 or 42 or 43 or 44 or 45 or 46 or 47 or 48 or 49 or 50 or 51 | 13,853,990 |
| TERM 4: Search keywords for ergogenic strategies | | |
| 53 | neuromuscular electrical stimulation*.af. | 5,674 |
| 54 | functional electrical stimulation*.af. | 8,405 |
| 55 | pharma*.af. | 12,474,502 |
| 56 | drug*.af. | 22,437,763 |
| 57 | caffeine.af. | 104,769 |
| 58 | boost*.af. | 282,121 |
| 59 | autonomic dysreflex*.af. | 3,357 |
| 60 | abdominal bind*.af. | 751 |
| 61 | anti-g*.af. | 72,928 |
| 62 | antigravity.af. | 1,448 |
| 63 | anti gravity.af. | 645 |
| 64 | spinal stim*.af. | 915 |
| 65 | spinal cord stim*.af. | 19,121 |
| 66 | positive pressure.af. | 50,549 |
| 67 | compression.af. | 398,162 |
| 68 | stocking*.af. | 24,623 |
| 69 | trunk muscle activ*.af. | 1,189 |
| 70 | trunk support.af. | 168 |
| 71 | precondition*.af. | 63,417 |
| 72 | stimulant *.af. | 100,201 |
| 73 | agonist*.af. | 795,755 |
| 74 | 53 or 54 or 55 or 56 or 57 or 58 or 59 or 60 or 61 or 62 or 63 or 64 or 65 or 66 or 67 or 68 or 69 or 70 or 71 or 72 or 73 | 26,619,069 |
| 75 | 13 and 39 and 52 and 74 | 7,239 |

**Supplementary Table S2.** Web of Science search strategy and results

| TERM 1: Search keywords for spinal cord injury | | |
| --- | --- | --- |
| #1 | **TS=(tetraplegia OR paraplegia OR quadriplegia OR spinal cord lesion OR spinal cord transection OR spinal cord impairment OR spinal injuries OR spinal cord injuries OR spinal lesion OR spinal transection OR spinal impairment OR spinal paralysis)** | 160,465 |
| TERM 2: Search keywords for exercise/performance | | |
| #2 | **TS=(exercise OR aerobic exercise OR exercise condition OR exercise prescription OR exercise therapy OR physical activity OR sport OR paralympic OR endurance exercise OR power output OR cardiorespiratory fitness OR oxygen uptake OR oxygen consumption OR functional capacity OR aerobic capacity OR performance OR enhance OR ergogenic OR time trial OR time to fatigue OR time to exhaustion OR sprint OR race)** | 12,418,401 |
| TERM 3: Search key words related to the cardiovascular system | | |
| #3 | **TS=(cardiovascular OR heart rate OR blood pressure OR stroke volume OR cardiac output OR venous return OR cardiac OR cardio OR blood flow OR haemodynamic OR hemodynamic OR vascular OR oxygen extraction OR arteriovenous)** | 3,213,942 |
| TERM 4: Search keywords for ergogenic strategies | | |
| #4 | **TS=(neuromuscular electrical stimulation OR functional electrical stimulation OR pharmaceutical OR pharmacological OR drug OR caffeine OR boost OR autonomic dysreflexia OR abdominal binding OR anti-gravity OR spinal stimulation OR spinal cord stimulation OR positive pressure OR compression OR stockings OR trunk muscle activation OR trunk support OR preconditioning OR stimulant OR agonist)** | 4,472,536 |
| #5 | **#1 AND #2 AND #3 AND #4** | 1,177 |
