# Supplementary material for: Strategies to Augment the Cardiovascular System and Acutely Enhance Exercise Performance in Individuals with Spinal Cord Injury: A Systematic Scoping Review
Source: Sports Med Open. 2025 Nov 6;11:125. doi: 10.1186/s40798-025-00909-7 (PMC12592629; doi:10.1186/s40798-025-00909-7)
Supplement: Supplementary file 2 — Supplementary Material 2. [file 40798_2025_909_MOESM2_ESM.docx]

**Supplementary Material S3 – S4**

**Strategies to augment the cardiovascular system and acutely enhance exercise performance in individuals with spinal cord injury: A systematic scoping review**

**Sports Medicine Open**

Hodgkiss, D.D ^1^, Balthazaar, S.J.T ^1,2,3^, Gee, C.M^2,4^, Chiou, SY^1^, Lucas, S.J.E^1,5^, Nightingale, T.E^1,2^

**^1^** School of Sport, Exercise and Rehabilitation Sciences, University of Birmingham, UK;

**^2^** International Collaboration on Repair Discoveries (ICORD), University of British Columbia, Vancouver, British Columbia, Canada. **^3^** Department of Cardiology, University Hospitals Birmingham National Health Service (NHS) Foundation Trust, Birmingham, UK; **^4^** Department of Orthopaedics, University of British Columbia, Vancouver, BC, Canada; ^5;^ Centre for Human Brain Health, University of Birmingham, United Kingdom.

**Corresponding author:** Tom E. Nightingale PhD, [T.E.Nightingale@bham.ac.uk](mailto:T.E.Nightingale@bham.ac.uk)

| **Supplementary Table S3. Mechanical strategies** | | | | | |
| --- | --- | --- | --- | --- | --- |
| **Study/**  **Country** | **Demographics** | **Ergogenic & Control Strategies** | **Exercise** | **Performance Outcomes** | **Psychophysiological Outcomes** |
| ***Abdominal binders*** | | | | | |
| Kerk  1995  USA  [1] | *N* = 6 (2M / 4F)  *Age* = 21.8 ± 4.4 years  *TSI* = 3.7 ± 0.98 years  *Classification* = 0T / 6P  *LOI* = T1-T6  *Severity* = 6 comp / 0 incomp  *Physical activity level* = Experienced road racing and/or track event athletes competing on the national or international level for at least 1 year. | Ergogenic strategy  Abdominal binder  *Model: Tartan Orthopedic Ltd, Denver, CO. The binder was placed to decrease the total girth measurement by 10% (~4-5cm across subjects).*  Control strategy  Without abdominal binder | Discontinuous submaximal exercise test on wheelchair rollers with incremental propulsion speeds until completion of the 6.3 m/s stage, followed by an incremental maximal exercise test to exhaustion. | ↔ % V̇O_2peak_ at 6.3m/s  Without strategy = 84.9 ± 3.4  With strategy = 85.7 ± 3.2  ↔ V̇O_2max_ (L/min)  Without strategy = 2.49 ± 0.19  With strategy = 2.50 ± 0.19 | ↔ HR (bpm)  Without strategy = 188 ± 6  With strategy = 189 ± 5  O_2_ pulse (mL/beat)  Without strategy = 13.2 ± 1.1  With strategy = 13.2 ± 1.1 |
| West  2014  United Kingdom  [2] | *N* = 8 (7M / 1F)  *Age* = 29 ± 2 years  *TSI* = 9 ± 3 years  *Classification* = 8T / 0P  *LOI* = C5-C7  *Severity* = 8 comp / 0 incomp  *Physical activity level* = National wheelchair rugby athletes, performing 15 hours/week of endurance, resistance and sport-specific training, and were familiar with treadmill exercise testing. | Ergogenic strategy  Abdominal binder  *Model: 493R Universal Back Support; McDavid Inc., Woodridge, USA. The binder incorporated a semirigid neoprene back panel with six plastic stays, flexible side-panels, and a flexible neoprene front panel with double Velcro fastening. The binder was individually fitted with the upper edge just beneath the costal margin so that it interfered minimally with rib cage movement. An inflatable rubber reservoir with a known volume of air was connected to a digital manometer and placed between the binder and the anterior abdominal wall. Binder tightness was adjusted until end-expiratory gastric pressure was approx twice that in the unbound condition.*  Control strategy  No abdominal binder | Maximal wheelchair test on a motorised treadmill at a fixed speed, cohen according to responses elicited during a prior submaximal test, and an increase in gradient of 0.2% every 40s. | ↑ V̇O_2_ (L/min)  Without strategy = 1.29 ± 0.33  With strategy = 1.43 ± 0.35  ↑ V̇O_2_ (mL/kg/min)  Without strategy = 19.0 ± 2.1  With strategy = 21.2 ± 2.8  ↔ Power output (W)  Without strategy = 49 ± 12  With strategy = 50 ± 13 | ↔ HR (bpm)  Without strategy = 120 ± 12  With strategy = 122 ± 13  ↔ V̇E (L/min)  Without strategy = 48.9 ± 14.1  With strategy = 46.1 ± 8.7  ↑ O_2_ pulse (mL/beat)  Without strategy = 10.7 ± 3.1  With strategy = 12.4 ± 3.2  ↔ SpO_2_ (%)  Without strategy = 95 ± 3  With strategy = 95 ± 3  ↔ RER  Without strategy = 1.08 ± 0.12  With strategy = 1.08 ± 0.13  ↔ RPE (dyspnea, Borg 0-10)  Without strategy = 7.0 ± 2.7  With strategy = 7.1 ± 2.9  ↔ RPE (arm discomfort, Borg 0-10)  Without strategy = 7.5 ± 2.0  With strategy = 7.4 ± 2.0  ↔ [La^-^]_b_ (mmol/L)  Without strategy = 4.6 ± 1.2  With strategy = 3.8 ± 1.0 |
| ***Lower body compression (anti-gravity suits/stockings)*** | | | | | |
| Brurok  2012  Norway  [3] | *High SCI (≥T6)*  *N* = 8 (8M / 0F)  *Age* = 35.0 ± 12.3 years  *TSI* = 12.9 ± 10.8 years  *Classification* = 4T / 4P  *LOI* = C4-T5  *Severity* = 8 comp / 0 incomp  *Physical activity level* = Moderately active  *Low SCI (<T6)*  *N* = 7 (5M / 2F)  *Age* = 43.6 ± 12.8 years  *TSI* = 13.5 ± 11.7 years  *Classification* = 0T / 7P  *LOI* = T8-T12  *Severity* = 7 comp / 0 incomp  *Physical activity level* = Moderately active | Ergogenic strategy  ACE + Leg Vascular Occlusion (LEVO)  *Bilateral lower extremity vascular occlusion, single-hose thigh cuffs (NIBP; Criticare, Waukesha, WI, USA) were inflated to 100 mmHg above resting SBP*  Control strategy  No abdominal binder | Four minutes of ACE at 30W followed by an incremental, graded test to volitional exhaustion. Increments were 5W/min and 10W/min for individuals with tetraplegia and paraplegia, respectively. | *High SCI (≥T6)*  ↑ V̇O_2peak_ (L/min)  Without strategy = 1.24 ± 0.40  With strategy = 1.48 ± 0.43  ↑ V̇O_2peak_ (mL/kg/min)  Without strategy = 17.7 ± 5.0  With strategy = 20.0 ± 5.0  ↑ Power output (W)  Without strategy = 72.5 ± 32.0  With strategy = 80.0 ± 34.0  *Low SCI (<T6)*  ↔ V̇O_2peak_ (L/min)  Without strategy = 1.74 ± 0.24  With strategy = 1.81 ± 0.24  ↔ V̇O_2peak_ (mL/kg/min)  Without strategy = 23.7 ± 3.6  With strategy = 24.4 ± 3.7  ↔ Power output (W)  Without strategy = 96 ± 23  With strategy = 100 ± 22 | *High SCI (≥T6)*  ↑ Ο_2_ pulse (mL/beat)  Without strategy = 11.7 ± 1.9  With strategy = 13.1 ± 2.5  ↔ HR_peak_ (bpm)  Without strategy = 149 ± 34  With strategy = 154 ± 33  ↔ V̇E (L/min)  Without strategy = 50.4 ± 20.8  With strategy = 54.3 ± 22.7  ↔ RER  Without strategy = 1.14 ± 0.07  With strategy = 1.20 ± 0.10  ↔ RPE (Borg 6-20)  Without strategy = 18 ± 1  With strategy = 18 ± 1  ↔ [La^-^]_b_ (mmol/L)  Without strategy = 7.5 ± 1.1  With strategy = 7.5 ± 1.2  *Low SCI (<T6)*  ↔ Ο_2_ pulse (mL/beat)  Without strategy = 12.8 ± 1.7  With strategy = 13.0 ± 1.5  ↔ HR_peak_ (bpm)  Without strategy = 185 ± 11  With strategy = 187 ± 9  ↔ V̇E (L/min)  Without strategy = 76.6 ± 13.4  With strategy = 80.8 ± 11.5  ↔ RER  Without strategy = 1.3 ± 0.1  With strategy = 1.3 ± 0.1  ↔ RPE (Borg 6-20)  Without strategy = 19 ± 1  With strategy = 19 ± 1  ↔ [La^-^]_b_ (mmol/L)  Without strategy = 9.3 ± 0.9  With strategy = 10.3 ± 1.5 |
| Hopman  1992  USA  [4] | *N* = 5 (5M / 0F)  *Age* = 37 ± 7 years  *TSI* = 9.6 ± 3.1 years  *Classification* = 0T / 5P  *LOI* = T6-T12  *Severity* = 5 comp / 0 incomp  *Physical activity level* = Competitive wheelchair marathon athletes (8.6 ± 2.9 hours of training per week). | Ergogenic strategy  Anti-gravity suit  *The standard Air Force anti-G suit (Anti-G Garment Cutaway CSU-13B/P) consisted of five interconnected bladders (two calf, two thigh, and one abdominal), with the hip and knee areas left uncovered. The suit was inflated with air from the abdominal bladder using a manual pump to a pressure of 52 mmHg (1 psi) within 5s.*  Control strategy  Sitting position | Submaximal ACE at 20%, 40%, 60% W_max_.  *Data presented for 60% W_max_ only.* | ↔ V̇O_2_ (L/min)  Without strategy = 1.35 ± 0.26  With strategy = 1.34 ± 0.25 | ↔ Q̇ (L/min)  Without strategy = 12.9 ± 3.5  With strategy = 12.2 ± 2.7  ↔ SV (mL)  Without strategy = 99 ± 30  With strategy = 104 ± 25  ↓ HR (bpm)  Without strategy = 132 ± 12  With strategy = 118 ± 13  ↑ SBP (mmHg)  Without strategy = 117  With strategy = 127  ↔ DBP (mmHg)  Without strategy = 78  With strategy = 79  O_2_ pulse (mL/beat)  Without strategy = 10.23 ± 2.18  With strategy = 11.36 ± 2.46 |
| Hopman 1998  USA  [5] | *Participants with tetraplegia*  *N* = 5 (5M / 0F)  *Age* = 34 ± 9 years  *TSI* = 11.4 ± 8.1 years  *Classification* = 5T / 0P  *LOI* = C5-C6  *Severity* = 5 comp / 0 incomp  *Physical activity level* = Low-to-moderately trained.  *Participants with paraplegia*  *N* = 4 (4M / 0F)  *Age* = 28 ± 7 years  *TSI* = 7 ± 4.5 years  *Classification* = 0T / 4P  *LOI* = T7-T12  *Severity* = 3 comp / 1 incomp  *Physical activity level* = Low-to-moderately trained. | Ergogenic strategy  Anti-gravity suit  *The anti-G suit (Anti-G Garment Cutaway CSU-13B/P) consisted of five interconnected bladders (two calf, two thigh, and one abdominal), with the hip and knee areas left uncovered. The suit was inflated using a foot pump to a pressure of 55 mmHg. The suit was deflated during the rest periods.*  Control strategy  Sitting position | Discontinuous maximal ACE test, consisting of three 7-min submaximal exercise bouts interspersed by 5-min rest periods up to 80% PPO, upon which intensity was increased by 2-10 W/min until exhaustion. | *Participants with tetraplegia*  ↔ V̇O_2peak_ (L/min)  Without strategy = 0.87 ± 0.24  With strategy = 0.87 ± 0.28  ↔ V̇O_2peak_ (mL/kg/min)  Without strategy = 12.7 ± 2.1  With strategy = 12.8 ± 2.9  ↔ PPO (W)  Without strategy = 42.0 ± 21.3  With strategy = 44.6 ± 22.5  *Participants with paraplegia*  ↔ V̇O_2peak_ (L/min)  Without strategy = 1.85 ± 0.22  With strategy = 1.86 ± 0.13  ↔ V̇O_2peak_ (mL/kg/min)  Without strategy = 26.1 ± 2.0  With strategy = 26.3 ± 2.6  ↔ PPO (W)  Without strategy = 96.5 ± 11.8  With strategy = 101.0 ± 10.5 | *Participants with tetraplegia*  ↔ HR (bpm)  Without strategy = 118 ± 29  With strategy = 106 ± 14  ↔ SBP (mmHg)  Without strategy = 77 ± 23  With strategy = 97 ± 9  ↔ DBP (mmHg)  Without strategy = 41 ± 17  With strategy = 60 ± 6  ↔ V̇E (L/min)  Without strategy = 41.5 ± 12.0  With strategy = 40.9 ± 16.3  ↔ RER  Without strategy = 1.09 ± 0.08  With strategy = 1.10 ± 0.09  ↓ RPE (Borg 6-20)  Without strategy = 18.4 ± 0.6  With strategy = 17.0 ± 1.2  *Participants with paraplegia*  ↔ HR (bpm)  Without strategy = 185 ± 4  With strategy = 178 ± 7  ↔ SBP (mmHg)  Without strategy = 135 ± 41  With strategy = 156 ± 43  ↔ DBP (mmHg)  Without strategy = 57 ± 15  With strategy = 60 ± 12  ↔ V̇E (L/min)  Without strategy = 81.7 ± 17.1  With strategy = 83.1 ± 19.8  ↔ RER  Without strategy = 1.21 ± 0.08  With strategy = 1.16 ± 0.14  ↔ RPE (Borg 6-20)  Without strategy = 17.8 ± 1.0  With strategy = 18.3 ± 0.5 |
| Houtman  1999  Netherlands  [6] | *N* = 5 (5M / 0F)  *Age* = 29 ± 5 years  *TSI* = 10.7 ± 7.6 years  *Classification* = NR  *LOI* = T6-L1  *Severity* = NR  *Physical activity level* = Well-trained | Ergogenic strategy  Lower body positive pressure  *The anti-g suit (CSU-13B/P, USAF) consisted of five interconnected bladders (one on the abdomen and two on the thigh and calf). The suit elicited a pulsating pressure alternating every 2s between 4.7 kPa and 9.3 kPa (35 and 70 mmHg, respectively).*  Control strategy  Anti-g suit worn in a deflated state | Incremental ACE test to exhaustion, beginning at 10W and increasing by 10W/min. | ↓ V̇O_2peak_ (mL/kg/min)  Without strategy = 30.0 ± 4.2  With strategy = 29.0 ± 3.9  ↔ PPO (W)  Without strategy = 120 ± 7  With strategy = 117 ± 8 | ↔ HR (bpm)  Without strategy = 175 ± 4  With strategy = 172 ± 8  ↔ RER  Without strategy = 1.22 ± 0.02  With strategy = 1.23 ± 0.05 |
| Kaprielian  1998  Canada  [7] | *Untrained participants*  *N* = 10 (10M / 0F)  *Age* = 23 ± 6 years  *TSI* = 10.7 ± 7.6 years  *Classification* = 0T / 10P  *LOI* = T6-T12  *Severity* = 10 comp / 0 incomp  *Physical activity level* = Inactive or occasionally participated in light recreational activities only.  *Trained participants*  *N* = 7 (7M/ 0F)  *Age* = 26 ± 6 years  *TSI* = 11.6 ± 7.1 years  *Classification* = 0T / 7P  *LOI* = T6-T12  *Severity* = 7 comp / 0 incomp  *Physical activity level* = Trained for at least 3 years, performing four training sessions per week including aerobic and resistance exercise. | Ergogenic strategy  Lower body positive pressure  *LBPP was applied using a LymphaPress pressure unit (Global Medical Imports, Mississauga, Ontario), that provides a pulsatile pressure by inflating 12 overlapping air bladders sequentially from the feet to the groin area in order to create a “milking” action on the lower limbs. The entire cycle lasts 30s (24s inflation, 2s pressure-hold, 4s deflation), with an inflation pressure set between 55 to 60 mmHg.*  Control strategy  No lower body positive pressure | Three submaximal ACE bouts were performed at power outputs designed to elicit heart rates of 100-110, 115-125 and 130-140 bpm. Each bout lasted 6-min, separated by 2-3 min of loadless cranking. Upon the end of the third submaximal exercise bout, participants performed a continuous incremental (10 W/min) maximal exercise test to exhaustion.  *Data are presented for submaximal physiological responses to ACE at a V̇O_2_ of 1.21 L/min (Q, SV, HR, SBP, DBP, MAP). Data are also presented for peak physiological responses to incremental, maximal ACE (*̇*V̇O_2peak_, PPO, HR_max_, V̇E_peak_, RER_max_).* | *Untrained participants*  ↔ V̇O_2peak_ (L/min)  Without strategy = 1.32 ± 0.25  With strategy = 1.39 ± 0.28  ↔ PPO (W)  Without strategy = 50.8 ± 12.6  With strategy = 52.0 ± 12.6  *Trained participants*  ↔ V̇O_2peak_ (L/min)  Without strategy = 2.21 ± 0.37  With strategy = 2.24 ± 0.37  ↔ PPO (W)  Without strategy = 85.0 ± 16.7  With strategy = 82.9 ± 16.7 | *Untrained participants*  ↔ Q̇ (L/min)  Without strategy = 10.3 ± 1.3  With strategy = 10.0 ± 0.9  ↔ SV (mL)  Without strategy = 66.5 ± 15.5  With strategy = 68.6 ± 16.4  ↓ HR (bpm)  Without strategy = 159 ± 14  With strategy = 140 ± 25  ↔ SBP (mmHg)  Without strategy = 141 ± 32  With strategy = 161 ± 25  ↔ DBP (mmHg)  Without strategy = 77 ± 16  With strategy = 82 ± 28  ↔ MAP (mmHg)  Without strategy = 96 ± 22  With strategy = 104 ± 19  ↔ HR_max_ (bpm)  Without strategy = 180 ± 13  With strategy = 175 ± 9  ↔ V̇E_peak_ (L/min)  Without strategy = 53.3 ± 15.8  With strategy = 56.2 ± 12.6  ↔ RER_max_  Without strategy = 1.10 ± 0.13  With strategy = 1.10 ± 0.09  *Trained participants*  ↔ Q̇ (L/min)  Without strategy = 10.0 ± 1.3  With strategy = 10.3 ± 1.1  ↔ SV (mL)  Without strategy = 78.7 ± 10.6  With strategy = 87.4 ± 10.6  ↓ HR (bpm)  Without strategy = 127 ± 8  With strategy = 117 ± 7  ↔ SBP (mmHg)  Without strategy = 143 ± 11  With strategy = 147 ± 11  ↔ DBP (mmHg)  Without strategy = 66 ± 8  With strategy = 67 ± 5  ↔ MAP (mmHg)  Without strategy = 92 ± 5  With strategy = 92 ± 5  ↔ HR_max_ (bpm)  Without strategy = 190 ± 11  With strategy = 186 ± 11  ↔ V̇E_peak_ (L/min)  Without strategy = 90.7 ± 18.5  With strategy = 93.4 ± 18.5  ↔ RER_max_  Without strategy = 1.24 ± 0.08  With strategy = 1.24 ± 0.08 |
| Pitetti  1994  USA  [8] | *N* = 10 (10M / 0F)  *Age* = 30.2 ± 7.7 years  *TSI* = 6 ± 3.5 years  *Classification* = 8T / 2P  *LOI* = C5-T11  *Severity* = 6 comp / 4 incomp  *Physical activity level* = NR | Ergogenic strategy  Low body positive pressure  *The fighter pilot anti-G suit consisted of air bladders over the abdomen and lower legs. Pressure within the suit fluctuated between 50 mmHg to 75 mmHg was reversed every 2-min to mimic muscle pump activity in the lower body musculature.*  Control strategy  No lower body positive pressure | Discontinuous, incremental ACE test to exhaustion. Resistance was increased by 5-10W every 3-min, with a 1-min rest period between stages to measure blood pressure.  Incremental wheelchair exercise performed on a motorised treadmill. Speed started between 2-4 mph at a grade of 0-3% and increased incrementally by 0.5-1.0% every 3-min to exhaustion.  *Peak and submaximal (measured at 50%V̇O_2peak_) cardiovascular responses are reported for ACE.*  *Statistical significance was set at p<0.025.* | *ACE test*  ↑ V̇O_2peak_ (L/min)  Without strategy = 0.839 ± 0.218  With strategy = 1.042 ± 0.212  ↑ V̇O_2peak_ (mL/kg/min)  Without strategy = 12.5 ± 2.0  With strategy = 15.7± 2.4  ↑ Work level (W)  Without strategy = 40 ± 13  With strategy = 50 ± 15  *Wheelchair exercise test*  ↑ V̇O_2peak_ (L/min)  Without strategy = 0.828 ± 0.312  With strategy = 0.960 ± 0.322  ↑ V̇O_2peak_ (mL/kg/min)  Without strategy = 11.24  With strategy = 14.3 | *ACE test*  ↔ HR_peak_ (bpm)  Without strategy = 125 ± 14  With strategy = 126 ± 12  ↑ V̇E_peak_ (L/min)  Without strategy = 35 ± 9  With strategy = 46 ± 17  ↔ RER_peak_  Without strategy = 1.10 ± 0.11  With strategy = 1.15 ± 0.19  Peak O_2_ pulse (mL/beat)  Without strategy = 6.71 ± 1.90  With strategy = 8.27 ± 1.86  ↔ HR _(50%V̇O2peak)_ (bpm)  Without strategy = 98 ± 10  With strategy = 93 ± 8  ↔ Q̇ _(50%V̇O2peak)_ (L/min)  Without strategy = 8.3 ± 2.5  With strategy = 8.8 ± 2.2  ↑ SV _(50%V̇O2peak)_ (mL)  Without strategy = 84 ± 20  With strategy = 94 ± 20  ↑ MAP _(50%V̇O2peak)_ (mmHg)  Without strategy = 71 ± 13  With strategy = 80 ± 14  ↔ (a-v)O_2 (50%V̇O2peak)_ (mL/100mL)  Without strategy = 8.3 ± 1.3  With strategy = 7.6 ± 1.0  ↔ TPR _(50%V̇O2peak)_ (dynes/s/cm_5_)  Without strategy = 748 ± 231  With strategy = 756 ± 159  *Wheelchair exercise test*  ↔ HR_peak_ (bpm)  Without strategy = 124 ± 20  With strategy = 126 ± 20  ↔ V̇E_peak_ (L/min)  Without strategy = 38.6 ± 14.0  With strategy = 41.4 ± 14.0  ↔ RER_peak_  Without strategy = 1.17 ± 0.21  With strategy = 1.24 ± 0.28  Peak O_2_ pulse (mL/beat)  Without strategy = 6.68 ± 2.74  With strategy = 7.62 ± 2.83 |
| Rimaud  2007  France  [9] | *Low paraplegia*  *N* = 9 (9M / 0F)  *Age* = 36.9 ± 11.8 years  *TSI* = 13.6 ± 10.0 years  *Classification* = 0T / 9P  *LOI* = T7-T12  *Severity* = 9 comp / 0 incomp  *Physical activity level* = Mixture of international athletes and individuals partaking in recreational activities (4.8 ± 2.4 hours of training per week).  *High paraplegia*  *N* = 5 (5M / 0F)  *Age* = 36.6 ± 11.3 years  *TSI* = 9.6 ± 7.6 years  *Classification* = 0T / 5P  *LOI* = T4-T5  *Severity* = 5 comp / 0 incomp  *Physical activity level* = Mixture of international athletes and individuals partaking in recreational activities (4.4 ± 2.6 hours of training per week). | Ergogenic strategy  Compression stockings  *Microfibers-2 tights with grip-top, graduated compression knee-length stockings (Olympique, Tournier-Bottu S.A., Gibaud Products, Saint-Etienne, France) were worn. The stockings created the greatest amount of elastic pressure at the ankle (21 mmHg), with decreased pressure up the leg to the top of the calf (15 mmHg).*  Control strategy  No compression socks | Maximal wheelchair exercise tests on a wheelchair ergometer. Participants rested with or without the stockings for 15-min, then completed a 6-min warm-up against no load. The load was then increased by 5W or 10W for high-level and low-level paraplegic participants, respectively, until volitional exhaustion. | *Low paraplegia*  ↔ V̇O_2peak_ (mL/kg/min)  Without strategy = 26.2 ± 6.9  With strategy = 24.8 ± 7.1  ↔ W_max_ (W)  Without strategy = 88 ± 32  With strategy = 91 ± 31  *High paraplegia*  ↔ V̇O_2peak_ (mL/kg/min)  Without strategy = 17.3 ± 5.3  With strategy = 16.8 ± 5.4  ↔ W_max_ (W)  Without strategy = 55 ± 22  With strategy = 52 ± 24 | *Low paraplegia*  ↔ HR (bpm)  Without strategy = 177 ± 15  With strategy = 177 ± 18  ↔ SBP (mmHg)  Without strategy = 141 ± 38  With strategy = 144 ± 40  ↔ DBP (mmHg)  Without strategy = 79 ± 13  With strategy = 80 ± 12  *High paraplegia*  ↔ HR (bpm)  Without strategy = 171 ± 14  With strategy = 169 ± 7  ↔ SBP (mmHg)  Without strategy = 127 ± 21  With strategy = 119 ± 37  ↔ DBP (mmHg)  Without strategy = 80 ± 16  With strategy = 84 ± 22 |
| Vaile et al. (2016)  Australia  [10] | *N* = 10 (10M / 0F)  *Age* = 30.2 ± 7.7 years  *TSI* = 13 ± 7 years  *Classification* = 10T / 0P  *LOI* = C5-C7  *Severity* = 3 comp / 7 incomp  *Physical activity level* = National wheelchair rugby athletes. | Ergogenic strategy  Compression socks  *Participants wore below-knee medical grade compression socks (Venosan, Class II).*  Control strategy  No compression socks | Wheelchair time trial. Participants first completed a time trial around an 86m standard court, the fastest lap time was used to prescribe the submaximal target intensity for the exercise trials. These trials consisted of 4 x 8-min exercise bouts (quarters) of fixed-intensity (set at 85% of each individual’s fastest lap time) laps around the court to replicate the duration of a typical wheelchair rugby game. Two-minute rest periods were implemented between quarters and 5-min for “half-time”.  *Performance data reported for Q4 only.* | ↔ Max sprint time (s)  Without strategy = 22.66 ± 2.61  With strategy = 22.63 ± 3.07  ↓ Average lap time (s)  Without strategy = 26.64 ± 3.70  With strategy = 24.77 ± 2.81 | ↔ HR  Without strategy = 128 ± 14  With strategy = 123 ± 11  ↑ ΔArm blood flow (mL/100mL/min)  Without strategy = 6.21 ± 5.73  With strategy = 10.77 ± 8.24  ↔ Δ Leg blood flow (mL/100mL/min)  Without strategy = 2.20 ± 1.85  With strategy = 2.51 ± 2.34  ↑ RPE (Borg 6-20)  Without strategy = 17 ± 3  With strategy =18 ± 2 |
| ***Passive leg exercise*** | | | | | |
| Torhaug  2018  Norway  [11] | *High SCI (≥T6)*  *N* = 8 (8M / 0F)  *Age* = 36.4 ± 13.5 years  *TSI* = 12.9 ± 10.8 years  *Classification* = NR  *LOI* = C4-T5  *Severity* = 8 comp / 0 incomp  *Physical activity level* = Moderately active.  *Low SCI (<T6)*  *N* = 7 (5M / 2F)  *Age* = 43.4 ± 12.7 years  *TSI* = 13.6 ± 11.7 years  *Classification* = NR  *LOI* = T8-T12  *Severity* = 7 comp / 0 incomp  *Physical activity level* = Moderately active. | Ergogenic strategy  ACE + Passive leg cycling (ACE-PLC)  Control strategy  ACE only | Graded, maximal exercise tests, consisting of 4 minutes of ACE at 30W followed by incremental stages of 5W/min and 10W/min to exhaustion for High SCI and Low SCI, respectively. Legs were passively moved by a technician at 70 rpm. | *High SCI (≥T6)*  ↔ V̇O_2peak_ (L/min)  Without strategy = 1.23 ± 0.40  With strategy = 1.56 ± 0.43  ↔ V̇O_2peak_ (mL/kg/min)  Without strategy = 17.6 ± 5.0  With strategy = 21.0 ± 3.8  ↔ Power output (W)  Without strategy = 72.5 ± 32  With strategy = 80 ± 20  *Low SCI (<T6)*  ↔ V̇O_2peak_ (L/min)  Without strategy = 1.74 ± 0.24  With strategy = 1.75 ± 0.31  ↔ V̇O_2peak_ (mL/kg/min)  Without strategy = 23.7 ± 3.6  With strategy = 23.6 ± 2.7  ↔ Power output (W)  Without strategy = 96 ± 23  With strategy = 97 ± 18 | *High SCI (≥T6)*  ↔ Ο_2_ pulse (mL/beat)  Without strategy = 8.3 ± 1.7  With strategy = 10.2 ± 1.3  ↔ HR_peak_ (bpm)  Without strategy = 149 ± 34  With strategy = 154 ± 30  ↔ V̇E (L/min)  Without strategy = 50.4 ± 20.8  With strategy = 56.7 ± 22.6  ↔ RER  Without strategy = 1.14 ± 0.07  With strategy = 1.18 ± 0.10  ↔ RPE (Borg 6-20)  Without strategy = 18 ± 1  With strategy = 18 ± 1  ↔ [La^-^]_b_ (mmol/L)  Without strategy = 7.5 ± 1.1  With strategy = 7.4 ± 1.2  *Low SCI (<T6)*  ↔ Ο_2_ pulse (mL/beat)  Without strategy = 9.4 ± 0.9  With strategy = 9.6 ± 1.4  ↔ HR_peak_ (bpm)  Without strategy = 185 ± 11  With strategy = 182 ± 11  ↔ V̇E (L/min)  Without strategy = 76.6 ± 13.4  With strategy = 74.7 ± 16.4  ↔ RER  Without strategy = 1.25 ± 0.11  With strategy = 1.24 ± 0.11  ↔ RPE (Borg 6-20)  Without strategy = 19 ± 1  With strategy = 18 ± 1  ↔ [La^-^]_b_ (mmol/L)  Without strategy = 9.3 ± 0.9  With strategy = 9.0 ± 1.8 |
| West  2015  Canada  [12] | *N* = 1 (1M / 0F)  *Age* = 43 years  *TSI* = 24 years  *Classification* = 1P / 0T  *LOI* = T3  *Severity* = 1 comp / 0 incomp  *Physical activity level* = The participant was previously a Paralympic wheelchair basketball player but had not trained for competitive sport for almost 5 years. They were still physically active and participated in recreational sport for 3-4.5 hours per week. | Ergogenic strategy  Active arm and passive leg exercise (AAPLE)  *Performed using a commercially available ergometer (IF PRO2; SCI-FIT, Tulsa, OK).*  Control strategy  ACE only | Maximal, incremental exercise tests consisting of incremental stages of 10W/min. | V̇O_2peak_ (L/min)  Without strategy = 1.38  With strategy = 1.49  V̇O_2peak_ (mL/kg/min)  Without strategy = 20.9  With strategy = 22.6  Power output (W)  Without strategy = 78  With strategy = 84 | Ο_2_ pulse (mL/beat)  Without strategy = 7.0  With strategy = 7.5  HR (bpm)  Without strategy = 195  With strategy = 198  V̇E (L/min)  Without strategy = 65.0  With strategy = 70.3  *f*_R_ (breaths/min)  Without strategy = 38  With strategy = 39  V_T_ (L)  Without strategy = 1.89  With strategy = 2.15  VCO_2_ (L/min)  Without strategy = 1.74  With strategy = 1.95  RER  Without strategy = 1.28  With strategy = 1.34  RPE (CR10)  Without strategy = 9  With strategy = 10 |
| ***Supine exercise*** | | | | | |
| Hopman  1998  USA  [5] | *Participants with tetraplegia*  *N* = 5 (5M / 0F)  *Age* = 34 ± 9 years  *TSI* = 11.4 ± 8.1 years  *Classification* = 5T / 0P  *LOI* = C5-C6  *Severity* = 5 comp / 0 incomp  *Physical activity level* = Low-to-moderately trained.  *Participants with paraplegia*  *N* = 4 (4M / 0F)  *Age* = 28 ± 7 years  *TSI* = 7 ± 4.5 years  *Classification* = 0T / 4P  *LOI* = T7-T12  *Severity* = 3 comp / 1 incomp  *Physical activity level* = Low-to-moderately trained. | Ergogenic strategy  Supine arm cycling  Control strategy  Sitting position | Discontinuous maximal ACE test, consisting of three 7-min submaximal exercise bouts interspersed by 5-min rest periods up to 80% PPO, upon which intensity was increased by 2-10 W/min until exhaustion. | *Participants with tetraplegia*  ↔ V̇O_2peak_ (L/min)  Without strategy = 0.87 ± 0.24  With strategy = 0.97 ± 0.24  ↑ V̇O_2peak_ (mL/kg/min)  Without strategy = 12.7 ± 2.1  With strategy = 14.2 ± 1.7  ↔ PPO (W)  Without strategy = 42.0 ± 21.3  With strategy =44.2 ± 19.3  *Participants with paraplegia*  ↔ V̇O_2peak_ (L/min)  Without strategy = 1.85 ± 0.22  With strategy = 1.81 ± 0.11  ↔ V̇O_2peak_ (mL/kg/min)  Without strategy = 26.1 ± 2.0  With strategy = 25.3 ± 2.4  ↔ PPO (W)  Without strategy = 96.5 ± 11.8  With strategy = 98.3 ± 7.6 | *Participants with tetraplegia*  ↔ HR (bpm)  Without strategy = 118 ± 29  With strategy = 123 ± 25  ↔ SBP (mmHg)  Without strategy = 77 ± 23  With strategy = 118 ± 12  ↔ DBP (mmHg)  Without strategy = 41 ± 17  With strategy = 51 ± 6  ↔ V̇E (L/min)  Without strategy = 41.5 ± 12.0  With strategy = 45.3 ± 15.8  ↔ RER  Without strategy = 1.09 ± 0.08  With strategy = 1.17 ± 0.11  ↔ RPE (Borg 6-20)  Without strategy = 18.4 ± 0.6  With strategy = 18.0 ± 1.0  *Participants with paraplegia*  ↓ HR (bpm)  Without strategy = 185 ± 4  With strategy = 178 ± 3  ↔ SBP (mmHg)  Without strategy = 135 ± 41  With strategy = 170 ± 52  ↔ DBP (mmHg)  Without strategy = 57 ± 15  With strategy = 74 ± 22  ↔ V̇E (L/min)  Without strategy = 81.7 ± 17.1  With strategy = 82.2 ± 14.7  ↔ RER  Without strategy = 1.21 ± 0.08  With strategy = 1.26 ± 0.11  ↔ RPE (Borg 6-20)  Without strategy = 17.8 ± 1.0  With strategy = 18.3 ± 0.5 |
| Hooker  1993  USA  [13] | *N* = 9 (9M / 0F)  *Age* = 33.0 ± 6.6 years  *TSI* = 9.5 ± 8.9 years  *Classification* = 0T / 9P  *LOI* = T1-T5  *Severity* = 4 comp / 5 incomp  *Physical activity level* = Sedentary or minimally active lifestyle and were not upper-body aerobically trained | Ergogenic strategy  Supine arm cycling  Control strategy  Sitting position | A 5-min unloaded ACE familiarisation period in the seated position was conducted. Following this, participants performed 3-min unloaded ACE in either the supine or seated position upon which the resistance was increased incrementally by 8.2 W/min until volitional exhaustion. | ↔ V̇O_2peak_ (L/min)  Without strategy = 1.10 ± 0.32  With strategy = 1.18 ± 0.29  ↔ V̇O_2peak_ (mL/kg/min)  Without strategy = 15.8 ± 6.5  With strategy = 17.0 ± 5.3  ↔ PPO (W)  Without strategy = 69 ± 15  With strategy = 71 ± 16 | ↔ HR_peak_ (bpm)  Without strategy = 151 ± 26  With strategy = 150 ± 23  ↔ O_2_ pulse (mL/beat)  Without strategy = 7.4 ± 1.9  With strategy = 7.4 ± 2.8  ↔ V̇E (L/min)  Without strategy = 42.9 ± 14.6  With strategy = 42.0 ± 15.6  ↔ RER  Without strategy = 1.07 ± 0.17  With strategy = 1.13 ± 0.14 |
| Data are reported as Mean ± SD, unless stated otherwise. ↑ = significantly greater relative to control strategy; ↓ = significantly lower relative to control; ↔ = no change or inconclusive. Outcomes without arrows were either calculated by reviewers from data included in the paper or statistical analysis was not reported to determine an effect. Some studies included multiple ergogenic strategies, hence why studies appear more than once. ACE, arm-crank ergometry; comp, motor-complete; F, females; incomp, motor-incomplete; LOI, level of injury; M, males; NR, not reported; P, paraplegia; OUES, oxygen uptake efficiency slope; PPO, peak power output; SCI, spinal cord injury; SV, stroke volume; T, tetraplegia; VO_2_, oxygen consumption; VA, alveolar ventilation; VT, tidal volume | | | | | |
| **Supplementary Table S4. Neuromodulatory strategies** | | | | | |
| **Study/**  **Country** | **Demographics** | **Ergogenic & Control Strategies** | **Exercise** | **Performance Outcomes** | **Psychophysiological Outcomes** |
| ***Autonomic dysreflexia (‘boosting’)*** | | | | | |
| Burnham  1994  Canada  [14] | *N* = 8 (8M / 0F)  *Age* = NR  *TSI* = NR  *Classification* = 8T / 0P  *LOI* = C6-C8  *Severity* = NR  *Physical activity level* = Elite athletes. | Ergogenic strategy  Boosting (bladder filling/clamped catheter) in 7 participants and prolonged sitting in 1 participant.  Control strategy  No boosting | Continuous graded maximal aerobic power test on friction-free rollers, starting with wheeling at 10 km/h, increasing in velocity by 2 km/h every 2-min until volitional exhaustion. *Data presented for end-race.*  Simulated 7.5 km road race on friction-free rollers with participants wheeling at maximum speed for the duration of the race. BP was collected at 3.5km during an imaginary downhill coasting section of the race whereby the rollers continued rolling during this period. *Data presented for mid-race [mid] and end-race [end], where possible.* | *Maximum aerobic power test*  ↑ V̇O_2peak_ (L/min)  Without strategy = 1.42 ± 0.33  With strategy =1.64 ± 0.25  *Simulated 7.5km road race*  ↓ Race time (min)  Without strategy = 25.6 ± 9.0  With strategy = 22.6 ± 6.6  ↑ V̇O_2_ (L/min) [mid]  Without strategy = 1.28 ± 0.28  With strategy = 1.51 ± 0.28  ↑ V̇O_2_ (L/min) [end]  Without strategy = 1.44 ± 0.26  With strategy = 1.68 ± 0.27 | *Maximum aerobic power test*  ↑ Noradrenaline (nmol/L)  Without strategy = 2.90 ± 1.10  With strategy = 4.47 ± 1.90  ↔ Adrenaline (nmol/L)  Without strategy = 0.17 ± 0.11  With strategy = 0.24 ± 0.09  ↔ HR (bpm)  Without strategy = 122 ± 16  With strategy = 134 ± 19  ↔ V̇E (L/min)  Without strategy = 69.2 ± 13.8  With strategy = 71.6 ± 17.0  ↔ RPE (Borg 6-20)  Without strategy = 17.4 ± 1.2  With strategy = 16 ± 2.6  ↔ RER  Without strategy = 1.22 ± 0.19  With strategy = 1.09 ± 0.11  ↔ Free fatty acids (mmol/L)  Without strategy = 0.37 ± 0.24  With strategy = 0.23 ± 0.10  ↔ Glucose (mmol/L)  Without strategy = 4.20 ± 0.46  With strategy = 4.30 ± 0.56  ↔ Lactate (mM)  Without strategy = 5.08 ± 1.42  With strategy = 6.11 ± 2.90  O_2_ pulse (mL/beat)  Without strategy = 11.64 ± 3.11  With strategy = 12.24 ± 2.55  *Simulated 7.5km road race*  ↑ Noradrenaline (nmol/L) [end]  Without strategy = 2.35 ± 0.83  With strategy = 7.10 ± 0.35  ↔ Adrenaline (nmol/L) [end]  Without strategy = 0.23 ± 0.21  With strategy = 0.23 ± 0.20  ↔ HR (bpm) [mid]  Without strategy = 111 ± 18  With strategy = 130 ± 16  ↔ HR (bpm) [end]  Without strategy = 119 ± 14  With strategy = 138 ± 22  ↔ V̇E (L/min) [mid]  Without strategy = 51.7 ± 11.4  With strategy = 62.0 ± 15.9  ↔ V̇E (L/min) [end]  Without strategy = 64.1 ± 15.2  With strategy = 71.9 ± 20.3  ↔ RPE (Borg 6-20) [mid]  Without strategy = 14.3 ± 1.4  With strategy = 13.9 ± 1.2  ↔ RPE (Borg 6-20) [end]  Without strategy = 16.5 ± 2.0  With strategy = 16.0 ± 1.5  ↓ SV (mL) [mid]  Without strategy = 109 ± 22  With strategy = 95 ± 20  ↔ Q̇ (L/min) [mid]  Without strategy = 12.08 ± 3.20  With strategy = 12.16 ± 2.50  ↑ (a-v)O_2_ diff (mL/100mL) [mid]  Without strategy = 10.50 ± 0.88  With strategy = 12.60 ± 2.00  ↔ RER [mid]  Without strategy = 0.99 ± 0.09  With strategy = 0.99 ± 0.05  ↔ RER [end]  Without strategy = 1.06 ± 0.17  With strategy = 1.06 ± 0.09  ↔ Free fatty acids (mmol/L)[end]  Without strategy = 0.35 ± 0.15  With strategy = 0.35 ± 0.12  ↔ Glucose (mmol/L) [end]  Without strategy = 4.00 ± 0.27  With strategy = 3.90 ± 0.38  ↔ Lactate (mM) [end]  Without strategy = 4.29 ± 1.38  With strategy = 4.24 ± 2.12  ↑ SBP (mmHg) [mid]  Without strategy = 105 ± 18  With strategy = 138 ± 23  ↑ SBP (mmHg) [end]  Without strategy = 99 ± 19  With strategy = 132 ± 32  ↔ DBP (mmHg) [mid]  Without strategy = 59 ± 6  With strategy = 78 ± 18  ↔ DBP (mmHg) [end]  Without strategy = 55 ± 8  With strategy = 62 ± 21  ↑ MAP (mmHg) [mid]  Without strategy = 74 ± 7  With strategy = 98 ± 19  ↔ MAP (mmHg) [end]  Without strategy = 69 ± 8  With strategy = 85 ± 23  O_2_ pulse (mL/beat)  Without strategy = 12.10 ± 2.61  With strategy = 12.17 ± 3.31 |
| Gee  2018  Canada  [15] | *N* = 1 (1M / 0F)  *Age* = NR  *TSI* = Chronic (>1 year)  *Classification* = 1T / 0P  *LOI* = C6  *Severity* = 1 comp / 0 incomp  *Physical activity level* = Elite wheelchair rugby athlete | Ergogenic strategy  Boosting via unintentional bladder overdistension  Control strategy  No AD | Field test consisting of twenty 20m sprints in alternating directions with 12s rest between sprints.  *Data presented for sprints 14-20, for 20m lengths only.* | Time (s)  Without strategy = 6.87 ± 0.05  With strategy = 6.70 ± 0.05 | HR (bpm)  Without strategy = 120 ± 1  With strategy = 141 ± 4  RPE (Borg 6-20)  Without strategy = 18 ± 0  With strategy = 15 ± 2 |
| Nightingale  2022  Canada  [16] | *N* = 1 (1M / 0F)  *Age* = 35 years  *TSI* = 7 years  *Classification* = 1T / 0P  *LOI* = C5  *Severity* = 1 comp / 0 incomp  *Physical activity level* = NR | Ergogenic strategy  Unintentional non-noxious stimuli  *The trigger for AD was likely bladder distension subsequent to bladder filling. A urinary tract infection was ruled out following. A subsequent urine culture. Prolonged sitting was also considered as a potential trigger for AD despite the use of multiple silicone gel pads. Participant described that his Botulinum toxin injections to treat overactive bladder may have become ineffective.*  Control strategy  No AD  *Foley catheter for continuous bladder drainage was fitted 4 days prior to testing and participant was encouraged to perform regular pressure releases.* | Standard incremental ACE CPET to exhaustion. Following >35 min rest, 4-min incremental ACE stages were performed at 20%, 40%, 60% and 80% of peak workload.  *Data presented for the second CPET with 4-min stages only (with AD during one visit and without AD on another).* | V̇O_2peak_ (mL/kg/min)  Without strategy = 15.14  With strategy = 18.75  PPO (W)  Without strategy = 65  With strategy = 84  Test duration (mm:ss)  Without strategy = 20:01  With strategy = 25:02 | HR (bpm)  Without strategy = 135  With strategy = 144  O_2_ pulse (mL/beat)  Without strategy = 9.33 ± 1.77  With strategy = 10.17 ± 1.91  V̇E (L/min)  Without strategy = 66.6  With strategy = 78.0  RER  Without strategy = 1.34  With strategy = 1.25  Δ SBP (pre-post ex) (mmHg)  Without strategy = 12  With strategy = 58  Δ DBP (pre-post ex) (mmHg)  Without strategy = -6  With strategy = 32  Δ MAP (pre-post ex) (mmHg)  Without strategy = -1  With strategy = 41 |
| Schmid  2001  Germany  [17] | *N* = 6 (6M / 0F)  *Age* = 36.7 ± 5.3 years  *TSI* = 10.5 ± 7.3 years  *Classification* = 5T / 1P  *LOI* = C7-T5  *Severity* = 2 comp / 4 incomp  *Physical activity level* = National wheelchair marathon racers and wheelchair rugby athletes (7.3 ± 6.4 hours of training per week). | Ergogenic strategy  AD (boosting)  *Fluid intake without miction or catheterisation was utilised to trigger AD.*  Control strategy  No AD  *Catheterisation and bowel care used to prevent AD.* | Incremental wheelchair ergometry test to volitional exhaustion. The test began at 20W with. A constant speed of 10 km/h, increasing by 10W every 3-min. | ↑ V̇O_2peak_ (L/min)  Without strategy = 1.85 ± 0.73  With strategy = 1.96 ± 0.70  ↑ PPO (W)  Without strategy = 72.5 ± 37  With strategy = 77.5 ± 36 | ↑ HR_peak_ (bpm)  Without strategy = 149 ± 24  With strategy = 161 ± 23  ↔ Lactate_peak_ (mmol/L)  Without strategy = 7.11 ± 2.10  With strategy = 7.00 ± 2.28  O_2_ pulse (mL/beat)  Without strategy = 12.42 ± 5.29  With strategy = 12.17 ± 4.68  ↑ Noradrenaline _max-ex_ (ng/mL)  Without strategy = 1.05 ± 0.75  With strategy = 0.67 ± 0.46  ↑ Adrenaline _max-ex_ (ng/mL)  Without strategy = 0.14 ± 0.07  With strategy = 0.20 ± 0.15  ↑ SBP _max-ex_ (mmHg)  Without strategy = 138 ± 36  With strategy = 172 ± 29  ↔ DBP _max-ex_ (mmHg)  Without strategy = 87 ± 17  With strategy = 97 ± 20 |
| ***Functional electrical stimulation*** | | | | | |
| Bakkum  2014  Netherlands  [18] | *N* = 9 (8M / 1F)  *Age* = 40 ± 13 years  *TSI* = 12 ± 10 years  *Classification* = 5T / 4P  *LOI* = C3-T11  *Severity* = 6 comp / 3 incomp  *Physical activity level* = NR | Ergogenic strategy  Hybrid cycling  *The hybrid cycle (BerkelBike Pro, BerkelBike B.V., St Michielsgesterl, The Netherlands) combined synchronous handcycling with asynchronous FES cycling. Stimulation was applied bilaterally over the quadriceps, hamstrings and gluteus muscles, with a pulse width of 400 µs, maximal current amplitude of 150 mA, and frequencies between 20-35 Hz. Current could be altered during cycling using steps of 15 mA.*  Control strategy  Hand cycling  *The handcycle (Speedy-Bike, Reha-Technik GmbH, Delbruck, Germany) was mounted on an ergotrainer (Tacx Flow, Technische Industrie, Tacx B.V., Wassenaar, The Netherlands).* | 2-minute warm up and cool down session of the specific test cycling. Before starting with the hybrid cycle test, five stimulation programs were tested to determine the most suitable program for exercise testing.  *Exercise bout performed at RPE level 6 on the Borg category ratio (vigorous).* | ↑ Metabolic Rate (kJ/min)  Without strategy = 20.48 ± 2.10  With strategy = 24.85 ± 2.69 | ↔ O_2_ pulse (mL/beat)  Without strategy = 9.59 ± 0.76  With strategy = 9.68 ± 0.66  ↑ HR (bpm)  Without strategy = 109 ± 7  With strategy = 124 ± 9 |
| Brurok  2013  Norway  [19] | *High-SCI*  *N* = 8 (8M / 0F)  *Age* = 35 ± 12 years  *TSI* = 13 ± 11 years  *Classification* = 4T / 4P  *LOI* = C4-T5  *Severity* = 8 comp / 0 incomp  *Physical activity level* = NR  *Low-SCI*  *N* = 7 (5M / 2F)  *Age* = 44 ± 13 years  *TSI* = 14 ± 12 years  *Classification* = 0T / 7P  *LOI* = T8-T12  *Severity* = 7 comp / 0 incomp  *Physical activity level* = NR | Ergogenic strategy  FES iso hybrid  *Performed on an ERGYS II system (Therapeutic Alliances, Inc., Fairborn, OH, USA) using the Motionstim 8 (Medel Electronics, Hamburg, Germany) system for FES, with stimulation applied over the quadriceps, hamstrings and gluteus muscles. Frequency was set at 40 Hz, with biphasic rectangular pulses at 500 µs pulse widths. Stimulation times were 3s on and 2s off. Maximum stimulation intensity was 125 mA.*  Control strategy  ACE  *Model: Ergomed 840L* | FES iso hybrid test was performed after the ACE test on a separate day. For ACE, participants performed a 6-min warm-up at 30W followed by incremental stages of 5-10W (which was typically 5W for tetraplegic and 10W for paraplegic) until volitional fatigue. For FES iso-hybrid, ergometer pedals were set in locked mode and stimulation intensity was increased by test personnel to maintain visible contractions. | *High-SCI*  ↑ V̇O_2peak_ (L/min)  Without strategy = 1.24 ± 0.40  With strategy = 1.74 ± 0.30  ↑ V̇O_2peak_ (mL/kg/min)  Without strategy = 17.6 ± 5.0  With strategy = 23.6 ± 3.6  ↔ PPO (W)  Without strategy = 72.5 ± 32  With strategy = 82.5 ± 27  *Low-SCI*  ↔ V̇O_2peak_ (L/min)  Without strategy = 1.74 ± 0.24  With strategy = 1.85 ± 0.32  ↔ V̇O_2peak_ (mL/kg/min)  Without strategy = 23.7 ± 3.6  With strategy = 25.2 ± 4.6  ↔ PPO (W)  Without strategy = 96 ± 23  With strategy = 98 ± 21 | *High-SCI*  ↑ O_2_ pulse (mL/beat)  Without strategy = 8.2 ± 1.7  With strategy = 10.8 ± 1.7  ↔ HR_peak_ (bpm)  Without strategy = 149 ± 34  With strategy = 161 ± 21  ↑ V̇E (L/min)  Without strategy = 50.4 ± 20.8  With strategy = 58.2 ± 20.7  ↔ RER  Without strategy = 1.14 ± 0.07  With strategy = 1.14 ± 0.07  ↔ [La^-^]_b_ (mmol/L)  Without strategy = 7.5 ± 1.1  With strategy = 8.4 ± 1.9  ↔ RPE  Without strategy = 18 ± 1  With strategy = 18 ± 1  *Low-SCI*  ↔ O_2_ pulse (mL/beat)  Without strategy = 9.4 ± 0.9  With strategy = 10.1 ± 1.4  ↔ HR_peak_ (bpm)  Without strategy = 185 ± 11  With strategy = 183 ± 10  ↔ V̇E (L/min)  Without strategy = 76.6 ± 13.4  With strategy = 82.0 ± 14.5  ↔ RER  Without strategy = 1.25 ± 0.1  With strategy = 1.23 ± 0.1  ↔ [La^-^]_b_ (mmol/L)  Without strategy = 9.3 ± 0.9  With strategy = 9.3 ± 1.8  ↔ RPE  Without strategy = 19 ± 1  With strategy = 19 ± 1 |
| Brurok  2013  Norway  [19] | *High-SCI*  *N* = 8 (8M / 0F)  *Age* = 35 ± 12 years  *TSI* = 13 ± 11 years  *Classification* = 4T / 4P  *LOI* = C4-T5  *Severity* = 8 comp / 0 incomp  *Physical activity level* = NR  *Low-SCI*  *N* = 7 (5M / 2F)  *Age* = 44 ± 13 years  *TSI* = 14 ± 12 years  *Classification* = 0T / 7P  *LOI* = T8-T12  *Severity* = 7 comp / 0 incomp  *Physical activity level* = NR | Ergogenic strategy  FES hybrid cycling  Control strategy  ACE  *Model: Ergomed 840L* | FES hybrid cycling was performed after the ACE test on a separate day. For ACE, participants performed a 6-min warm-up at 30W followed by incremental stages of 5-10W (which was typically 5W for tetraplegic and 10W for paraplegic) until volitional fatigue. For FES hybrid cycling, 2 mins of manually assisted FES cycling warm-up movements were conducted at 50% intensity of muscle thresholds. No resistance was provided during peak FES hybrid cycling. | *High-SCI:*  ↑ V̇O_2peak_ (L/min)  Without strategy = 1.24 ± 0.40  With strategy = 1.80 ± 0.40  ↑ V̇O_2peak_ (mL/kg/min)  Without strategy = 17.6 ± 5.0  With strategy = 24.4 ± 4.1  ↑ PPO (W)  Without strategy = 72.5 ± 32  With strategy = 83.0 ± 32  *Low-SCI*  ↔ V̇O_2peak_ (L/min)  Without strategy = 1.74 ± 0.24  With strategy = 1.89 ± 0.38  ↔ V̇O_2peak_ (mL/kg/min)  Without strategy = 23.7 ± 3.6  With strategy = 25.6 ± 4.1  ↔ PPO (W)  Without strategy = 96 ± 23  With strategy = 98 ± 19 | *High-SCI*  ↑ O_2_ pulse (mL/beat)  Without strategy = 8.2 ± 1.7  With strategy = 11.0 ± 2.0  ↔ HR_peak_ (bpm)  Without strategy = 149 ± 34  With strategy = 163 ± 20  ↑ V̇E (L/min)  Without strategy = 50.4 ± 20.8  With strategy = 61.4 ± 19.8  ↔ RER  Without strategy = 1.14 ± 0.07  With strategy = 1.18 ± 0.07  ↑ [La^-^]_b_ (mmol/L)  Without strategy = 7.5 ± 1.1  With strategy = 9.4 ± 1.7  ↔ RPE  Without strategy = 18 ± 1  With strategy = 18 ± 1  *Low-SCI*  ↔ O_2_ pulse (mL/beat)  Without strategy = 9.4 ± 0.9  hybrid = 10.4 ± 1.8  ↔ HR_peak_ (bpm)  Without strategy = 185 ± 11  With strategy = 182 ± 9  ↔ V̇E (L/min)  Without strategy = 76.6 ± 13.4  With strategy = 80.2 ± 21.1  ↔ RER  Without strategy = 1.25 ± 0.1  With strategy = 1.23 ± 0.1  ↔ [La^-^]_b_ (mmol/L)  Without strategy = 9.3 ± 0.9  With strategy = 10.6 ± 2.5  ↔ RPE  Without strategy = 19 ± 1  With strategy = 19 ± 1 |
| Hasnan  2013  Australia  [20] | *N* = 9 (9M / 0F)  *Age* = 40.6 ± 3.3 years  *TSI* = 6.6 ± 1.2 years  *Classification* = NR  *LOI* = C6-T12  *Severity* = CD  *Physical activity level* = Recreationally active | Ergogenic strategy  Combined ACE and FES leg cycle exercise (ACE+FES-LCE)  *For FES, electrodes were placed over the quadriceps, hamstrings, and glutei muscles groups. Stimulation was delivered via biphasic rectangular pulses at 35 Hz and 300 µs pulse width. The “firing” angles were fixed and timings were preset by a computer program. The maximum stimulation amplitude was limited to 140 mA.*  Control strategy  Arm crank ergometry (ACE) | All participants were assessed in the same order. Firstly, participants underwent a combined maximal ACE and maximal FES-LCE test (as described above). Following the maximal exercise test, participants performed submaximal exercise with intensity increasing incrementally until reaching a period of steady state at 40%, 60%, and 80%V̇O_2peak_. Participants had a short recovery period after reaching each intensity.  *Data for submaximal exercise presented for 80%V̇O_2peak_ only.* | ↑ Power output (W)  Without strategy = 74.4 ± 22.5  With strategy = 100.7 ± 24.9  ↑ V̇O_2peak_ (mL/kg/min)  Without strategy = 18.4 ± 5.1  With strategy = 20.8 ± 5.1 | Maximal exercise  ↔ V̇_E_ (L/min)  Without strategy = 49.4 ± 8.7  With strategy = 63.7 ± 13.8  ↔ RER  Without strategy = 1.36 ± 0.15  With strategy = 1.47 ± 0.18  ↑ Lactate (mmol/L)  Without strategy = 6.3 ± 1.5  With strategy = 9.9 ± 2.7  Submaximal exercise  ↔ HR (bpm)  Without strategy = 145 ± 12  With strategy = 150 ± 12  ↔ Q̇ (L/min)  Without strategy = 12.9 ± 2.8  With strategy = 14.5 ± 2.1  ↔ SV (mL)  Without strategy = 77 ± 36  With strategy = 87 ± 18  ↔ (Ca-Cv)V̇O_2_  (mL/100mL/min)  Without strategy = 9.0 ± 0.9  With strategy = 9.7 ± 0.9  ↑ Lactate (mmol/L)  Without strategy = 5.7 ± 1.5  With strategy = 7.4 ± 2.4 |
| Hasnan  2013  Australia  [20] | *N* = 9 (9M / 0F)  *Age* = 40.6 ± 3.3 years  *TSI* = 6.6 ± 1.2 years  *Classification* = NR  *LOI* = C6-T12  *Severity* = CD  *Physical activity level* = Recreationally active | Ergogenic strategy  Hybrid cycling (arm and leg tricycle)  *Model: HYBRID (Berkelboke BV, ‘s-Hertogenbosch, the Netherlands). Participants had their feet and legs strapped and held in position by leg supports. The Hybrid ergometer was mounted on a stationary cycle resistance trainer which calculated external power output during combined arm and leg effort.*  Control strategy  Arm crank ergometry (ACE) | Firstly, participants performed maximal exercise tests. For ACE, participants warmed up at 0W for 3-min followed by incremental stages of 5-10W/min until volitional exhaustion. For Hybrid cycling, participants warmed-up at 0W for 3-min followed by incremental stages of 10W/min. The electrical stimulation was increased manually in four increments (minimum contraction, 33%, 66% and 100% maximum amplitude of 140 mA) at equivalent resting HR, 33%, 66% and 100% of maximum HR reserve; the goal of this ws to exhaust the arms and legs simultaneously. Following the maximal exercise test, participants performed submaximal exercise with intensity increasing incrementally until reaching a period of steady state at 40%, 60%, and 80%V̇O_2peak_. Participants had a short recovery period after reaching each intensity.  *Data for submaximal exercise presented for 80%V̇O_2peak_ only.* | ↔ Power output (W)  Without strategy = 74.4 ± 22.5  With strategy = 75.6 ± 15.0  ↔ V̇O_2peak_ (mL/kg/min)  Without strategy = 18.4 ± 5.1  With strategy = 21.5 ± 4.8 | Maximal exercise  ↔ V̇_E_ (L/min)  Without strategy = 49.4 ± 8.7  With strategy = 64.8 ± 19.8  ↔ RER  Without strategy = 1.36 ± 0.15  With strategy = 1.35 ± 0.12  ↑ Lactate (mmol/L)  Without strategy = 6.3 ± 1.5  With strategy = 9.2 ± 1.  Submaximal exercise  ↔ HR (bpm)  Without strategy = 145 ± 12  With strategy = 144 ± 12  ↔ Q̇ (L/min)  Without strategy = 12.9 ± 2.7  With strategy = 14.3 ± 2.1  ↔ SV (mL)  Without strategy = 77 ± 36  With strategy = 101 ± 33  ↔ (Ca-Cv)V̇O_2_  (mL/100mL/min)  Without strategy = 9.0 ± 0.9  With strategy = 9.4 ± 2.4  ↑ Lactate (mmol/L)  Without strategy = 5.7 ± 1.5  With strategy = 8.3 ± 1.5 |
| Hooker  1992  USA  [21] | *N* = 8 (7M / 1F)  *Age* = 32.6 ± 4.7 years  *TSI* = 7.6 ± 4.9 years  *Classification* = 8T / 0P  *LOI* = C5-C8  *Severity* = 1 comp / 7 incomp  *Physical activity level* = Had recently completed a 12-15 week FES-LCE intervention | Ergogenic strategy  Hybrid cycling (ACE+FES-LCE)  *FES-LCE was performed using the ERGYS I FES leg cycle ergometer (Therapeutic Technologies Inc., Tampa, FL, USA). Electrodes were placed over the quadriceps, hamstrings and glutei muscle groups, and stimulation was provided by monophasic rectangular-wave pulses of 0.375 ms duration at 35 Hz. Maximal current output was limited to ~130 mA. Participants with incomplete spinal lesions and minimal lower limb motor function were instructed not to provide voluntary muscle contractions during FES-LCE. The selected power output for FES-LCE was the highest power output that could be maintained at 50 rpm for 20 min continuously.*  Control strategy  Arm crank exercise (ACE) | Each participant completed an exercise session consisting of the following sequence: 5 min seated rest; 10 min upright ACE; 10 min seated rest; 1 min passive (technician-assisted) LCE; 1 min FES-LCE at 0W (unloaded flywheel); 1 min FES-LCE during which current output was gradually increased to the level required to maintain at 50 rpm; 10 min FES-LCE; 10 min hybrid exercise and 2 min passive LCE. | ↑ V̇O_2_ (L/min)  Without strategy = 0.66 ± 0.06  With strategy = 1.02 ± 0.06  Power Output (W)  Without strategy = 19.4 ± 10.5  With strategy = 22.4 ± 12.7 | ↑ Pulmonary Ventilation (L/min)  Without strategy = 25.9 ± 2.0  With strategy = 39.7 ± 2.3  ↑ HR (bpm)  Without strategy = 99 ± 6  With strategy = 117 ± 3  ↑ SV (mL)  Without strategy = 57 ± 8  With strategy = 80 ± 6  ↑ Q̇ (L/min)  Without strategy = 6.4 ± 0.6  With strategy = 9.4 ± 0.6  ↔ a-vO_2_ diff (mL/100mL)  Without strategy = 10.7 ± 0.8  With strategy = 11.2 ± 0.8  ↔ MAP (mmHg)  Without strategy = 72 ± 4  With strategy = 71 ± 4  ↓ TPR (mmHg/L/min)  Without strategy = 10.9 ± 0.4  With strategy = 7.2 ± 0.4  O_2_ pulse (mL/beat)  Without strategy = 6.7 ± 0.2  With strategy = 8.7 ± 0.2 |
| Hopman 1998  USA  [5] | *Participants with tetraplegia*  *N* = 5 (5M / 0F)  *Age* = 34 ± 9 years  *TSI* = 11.4 ± 8.1 years  *Classification* = 5T / 0P  *LOI* = C5-C6  *Severity* = 5 comp / 0 incomp  *Physical activity level* = Low-to-moderately trained.  *Participants with paraplegia*  *N* = 4 (4M / 0F)  *Age* = 28 ± 7 years  *TSI* = 7 ± 4.5 years  *Classification* = 0T / 4P  *LOI* = T7-T12  *Severity* = 3 comp / 1 incomp  *Physical activity level* = Low-to-moderately trained. | Ergogenic strategy  FES  *FES was applied to both lower limbs with two EMPI “Focus” Neuromuscular Stimulators (EMPI Inc, St. Paul Minnesota) via electrodes over the quadriceps, hamstrings, gastrocnemius and tibialis anterior. Stimulation consisted of symmetric biphasic pulses of 300 ms at 35 Hz delivered across a 1000 ohm load at 80 mA, over a duty cycle of 2.5s “on” and 5s “off”, with a 2s “rise” and “fall” time for each pulse.*  Control strategy  Sitting position | Following a pretest for familiarisation, five discontinuous maximal exercise tests were performed on different days under the following conditions: 1) sitting, 2) supine, 3) sitting with G-suit/ stockings/ abdominal binder/ FES of legs. Tests were assigned in random order with one day between tests. Each session consisted of submaximal and maximal exercise. Test was terminated when the cadence fell below 60 rpm. | *Participants with tetraplegia*  ↔ V̇O_2peak_ (L/min)  Without strategy = 0.87 ± 0.24  With strategy = 0.92 ± 0.21  ↔ V̇O_2peak_ (mL/kg/min)  Without strategy = 12.7 ± 2.1  With strategy = 13.4 ± 1.4  ↔ PPO (W)  Without strategy = 42.0 ± 21.3  With strategy = 39.8 ± 20.9  *Participants with paraplegia*  ↔ V̇O_2peak_ (L/min)  Without strategy = 1.85 ± 0.22  With strategy = 1.87 ± 0.12  ↔ V̇O_2peak_ (mL/kg/min)  Without strategy = 26.1 ± 2.0  With strategy = 26.6 ± 3.1  ↔ PPO (W)  Without strategy = 96.5 ± 11.8  With strategy = 99.8 ± 8.8 | *Participants with tetraplegia*  ↔ HR (bpm)  Without strategy = 118 ± 29  With strategy = 106 ± 14  ↔ SBP (mmHg)  Without strategy = 77 ± 23  With strategy = 104 ± 22  ↔ DBP (mmHg)  Without strategy = 41 ± 17  With strategy = 59 ± 12  ↔ V̇E (L/min)  Without strategy = 41.5 ± 12.0  With strategy = 39.1 ± 8.6  ↔ RER  Without strategy = 1.09 ± 0.08  With strategy = 1.06 ± 0.08  ↔ RPE (Borg 6-20)  Without strategy = 18.4 ± 0.6  With strategy = 17.8 ± 0.8  *Participants with paraplegia*  ↔ HR (bpm)  Without strategy = 185 ± 4  With strategy = 178 ± 7  ↔ SBP (mmHg)  Without strategy = 135 ± 41  With strategy = 164 ± 31  ↔ DBP (mmHg)  Without strategy = 57 ± 15  With strategy = 62 ± 4  ↔ V̇E (L/min)  Without strategy = 81.7 ± 17.1  With strategy = 84.6 ± 19.9  ↔ RER  Without strategy = 1.21 ± 0.08  With strategy = 1.19 ± 0.12  ↔ RPE (Borg 6-20)  Without strategy = 17.8 ± 1.0  With strategy = 17.5 ± 1.3 |
| Laskin  1993  Canada  [22] | *N* = 8 (7M / 1F)  *Age* = 27.9 ± 4.2 years  *TSI* = 8.1 ± 6 years  *Classification* = 6T / 2P  *LOI* = C6-T6  *Severity* = NR  *Physical activity level* = NR | Ergogenic strategy  Simultaneous arm row and stimulation  *FES was applied over both the quadriceps and hamstring muscle groups, with adjustments made to the on-off cadence and level of stimulation in order to propel backwards and forwards. The apparatus incorporated a bucket chair for trunk stabilization, leg supports for biomechanical efficiency, and an elastic 'leash' which acts as a brake on extension and biases the movement towards flexion (to aid the generally weaker hamstring muscles). Stimulation was achieved by use of a hand-held stimulator ('Quadstim', Biomech Designs Ltd) with a specially designed adaptor to allow the cadence to be altered.*  Control strategy  Arm row only | Exercise was performed for 10-minutes for each strategy, with rest periods of 1:3 allowed for values to return to baseline. The order of the tests was randomised to control for a cumulative fatigue effect. | ↑ %V̇O_2peak_ (% max)  Without strategy = 71.0 ± 9.3  With strategy = 82.9 ± 9.6  ↑ V̇O_2_ (L/min)  Without strategy = 0.825 ± 0.17  With strategy = 1.02 ± 0.15  ↑ V̇O_2_ (mL/kg/min)  Without strategy = 13.27 ± 2.86  With strategy = 16.34 ± 2.09 | ↔ V̇_E_ (L/min)  Without strategy = 32.9 ± 7.35  With strategy = 34.4 ± 8.77  ↔ RER  Without strategy = 0.99 ± 0.08  With strategy = 0.96 ± 0.06  ↔ HR (bpm)  Without strategy = 112 ± 10  With strategy = 105 ± 8  ↑ MAP (mmHg)  Without strategy = 76.7 ± 3.9  With strategy = 96.5 ± 4.0  O_2_ pulse (mL/beat)  Without strategy = 7.37 ± 2.39  With strategy = 9.71 ± 2.56 |
| Phillips  1995  USA  [23] | *N* = 8 (7M / 1F)  *Age* = 33 ± 8 years  *TSI* = 6 ± 4 years  *Classification* = 3T / 5P  *LOI* = C6-T12  *Severity* = 7 comp / 1 incomp  *Physical activity level* = NR | Ergogenic strategy  ACE+FNS 80 mA (80%V̇O_2peak_)  *Functional neuromuscular stimulation (FNS) was applied to both lower limbs (EMPI Inc., St Paul, MN, USA), over the motor points of each major muscle (quadriceps, hamstrings, gastrocnemius, and tibialis anterior). Stimulation consisted of symmetric biphasic pulses of 300 ms at 35 Hz delivered across a 1000-ohm load, over a “duty cycle” of 2.5-s “on” and 5-s “off”, with a 2-s “rise time” and a 2-s “fall time” for each pulse.*  Control strategy  ACE (80%V̇O_2peak_) | 3 minutes gradual warm-up to 5 minutes ACE with or without FNS (80mA) at 80% VO_2peak_. Upper and lower leg muscles were stimulated alternately, with the stim pattern set so that the fall time of the upper leg muscles coincided with the rise time of the lower leg muscles, and vice versa.  *Study reports exercise performed with low (40 mA) and high FNS (80 mA) at 60% and 80%V̇O_2peak_, but only 80 mA at V̇O_2peak_ is reported herein.* | ↑ V̇O_2peak_ (mL/kg/min)  Without strategy = 14.8 ± 4.5  With strategy = 17.2 ± 3.8 | ↔ RER  Without strategy = 0.94 ± 0.10  With strategy = 0.95 ± 0.06  ↔ V̇_E_ (L/min)  Without strategy = 37 ± 14  With strategy = 39 ± 15  ↔ HR (bpm)  Without strategy = 131 ± 30  With strategy = 127 ± 33  ↔ RPE (Borg 6-20)  Without strategy = 14.8 ± 1.2  With strategy = 15.1 ± 1.2 |
| Raymond  1997  Australia  [24] | *N* = 7 (7M / 0F)  *Age* = 31.9 ± 7.1 years  *TSI* = NR  *Classification* = 0T / 7P  *LOI* = T4-T12  *Severity* = NR  *Physical activity level* = NR | Ergogenic strategy  ACE+ES-LCE  *Stimulation electrodes were placed over the motor points of the gluteal, quadricep and hamstring muscle groups. The leg cycle ergometer (Ergys 1, Therapeutic Alliances Inc., Dayton. USA) was placed underneath the ACE to allow simultaneous arm-cranking and leg cycling.*  Control strategy  ACE | ACE resistance was set at 65%V̇O_2peak_ and leg resistance was set at the highest level the participants could tolerate for 5-min (determined prior to testing). At the end of 5-min combined exercise, participants continued for a further 5-min of only arms exercise. | ↑ Steady state V̇O_2_ (L/min)  Without strategy = 1.26 ± 0.34  With strategy = 1.58 ± 0.32  ↑ Total power output (W)  Without strategy = 29.6 ± 16.7  With strategy = 34.9 ± 19.6 | ↓ HR (bpm)  Without strategy = 149 ± 16  With strategy = 132 ± 13  ↑ O_2_ pulse (mL/beat)  Without strategy = 8.6 ± 3.2  With strategy = 12.2 ± 3.2  ↑ V̇CO_2_ (L/min)  Without strategy = 1.24 ± 0.26  With strategy = 1.56 ± 0.21  ↔ Steady state V̇E (L/min)  Without strategy = 43.8 ± 10.3  With strategy = 44.7 ± 5.8 |
| Raymond  1999  Australia  [25] | *N* = 10 (10M / 0F)  *Age* = 36 ± 6.3 years  *TSI* = NR  *Classification* = 0T / 10P  *LOI* = T5-T12  *Severity* = NR  *Physical activity level* = NR | Ergogenic strategy  ACE+ES-LCE  *Stimulation electrodes were placed over the motor points of the gluteal, quadricep and hamstring muscle groups. During cycling, stimulation was delivered via monophasic, rectangular pulses at a frequency of 35 Hz and duration of 0.375 ms. The amplitude and timing of the stimulation were preset by a microprocessor which received feedback based on crank position and velocity. Electrical stimulation output to the leg muscles was varied by the microprocessor to maintain a cycling cadence of 50 rev·min^−1^. The maximum stimulus delivered to the muscle was 132 mA and the minimum cycling cadence was 35 rev·min^-1^.*  Control strategy  ACE | For the maximal ACE test, workload was increased by 5-10W/min until exhaustion. For the maximal ACE+ES-LCE test, participants performed the same protocol as the ACE test, but while simultaneously performing FES-induced leg cycling against 0W. Following maximal exercise tests, participants performed submaximal steady state exercise. All participants were tested on different days in a randomized order and was preceded by a 5-min seated rest period. For ACE alone, participants arm-cranked at 50 rpm for 5-min at 25W and 50W power outputs, separated by a 5-min recovery period. The test was terminated upon request or following the workload that elicited an exercise HR of 75% age-predicted maximum. For ACE+ES-LCE, adhered to the same protocol as the ACE test while simultaneously performing ES-induced leg cycling. All participants (N = 10) were able to cycle against 0W, and those who were able to do so cycled against 6W (N = 6) and 12W (N = 2) for each respective arm crank workload. Those who could leg cycle against 6W but not 12W, cycled against 0W for the second submaximal work-load.  *Note, two trials are reported in Table 1 (Trial 1: N=10, six participants performed ES-LCE at 6W and four performed ES-LCE at 0W; Trial 2: N=7, two participants performed ES-LCE at 12W and five performed ES-LCE at 0W). Only data from Trial 1 is displayed herein.* | Maximal exercise  ↔ Power Output (W)  Without strategy = 85.3 ± 21  With strategy = 79.5 ± 20  ↑ V̇O_2_ (L/min)  Without strategy = 1.81 ± 0.32  With strategy = 2.04 ± 0.25  Submaximal exercise  ↔ Total Power Output (W)  Without strategy = 25.5 ± 0.6  With strategy = 28.0 ± 2.8  ↓ Efficiency (%)  Without strategy = 8.2 ± 0.9  With strategy = 6.7 ± 1.3  ↑ V̇O_2_ (L/min)  Without strategy = 0.91 ± 0.09  With strategy = 1.19 ± 0.16 | Maximal exercise  ↔ HR_peak_ (bpm)  Without strategy = 175 ± 13  With strategy = 177 ± 6  Submaximal exercise  ↔ HR (bpm)  Without strategy = 121 ± 19  With strategy = 113 ± 16  ↑ a-vO2 diff (mL/100 mL)  Without strategy = 8.6 ± 1.3  With strategy = 10.5 ± 1.9  ↑ V̇_E_ (L/min)  Without strategy = 27.6 ± 3.8  With strategy = 37.0 ± 7.0  ↑ SV(mL)  Without strategy = 89 ± 14  With strategy = 91 ± 12  ↔ Q̇ (L/min)  Without strategy = 2.9 ± 0.2  With strategy = 2.9 ± 0.2 |
| Shaffer  2018  USA  [26] | *N* = 24 (22M / 2F)*  *Age* = 29.1 ± 1.5 years  *TSI* = 16 participants < 2 years, 8 participants > 2 years  *Classification* = 7T / 17P  *LOI* = C4-T8  *Severity** = 22 comp / 2 incomp  *Physical activity level* = NR  **Note, two participants used compression socks and one also used an abdominal binder.* | Ergogenic strategy  Hybrid FES rowing  *Model: Concept-2 adapted rowing machine (Morrisville, VT, USA).*  Control strategy  Arms only rowing | Graded FES rowing maximal exercise test to exhaustion, with workload increasing every 1-2 min by 5-10W. | ↑ V̇O_2peak_ (mL/kg/min)  Without strategy = 16.4 ± 5.4  With strategy = 20.4 ± 5.4 | ↑ RER_peak_  Without strategy = 1.26 ± 0.10  With strategy = 1.17 ± 0.05  ↔ HR_peak_ (bpm)  Without strategy = 160 ± 29  With strategy = 163 ± 25  ↔ V̇E_peak_ (L/min)  Without strategy = 49.5 ± 17.6  With strategy = 50.0 ± 14.2 |
| Torhaug  2018  Norway  [11] | **High SCI (≥T6)**  *N* = 8 (8M / 0F)  *Age* = 36.4 ± 13.5 years  *TSI* = 12.9 ± 10.8 years  *Classification* = NR  *LOI* = C4-T5  *Severity* = 8 comp / 0 incomp  *Physical activity level* = Moderately active  **Low SCI (<T6)**  *N* = 7  *Age* = 43.4 ± 12.7 years  *TSI* = 13.6 ± 11.7 years  *Classification* = NR  *LOI* = T8-T12  *Severity* = 7 comp / 0 incomp  *Physical activity level* = Moderately active | Ergogenic strategy  FES hybrid cycling  *Electrical stimulation was delivered at an impulse frequency of 40 Hz and amplitude of 140 mA. Where necessary, the test personnel provided manual assistance to ensure that pedalling cadence did not drop below a default preset stimulation cut-off speed of 35 rpm and to ensure stimulation current throughout the test.*  Control strategy  ACE only | Graded, maximal exercise tests. ACE consisted of 4 minutes at 30W followed by incremental stages of 5W/min and 10W/min to exhaustion for High SCI and Low SCI, respectively. Hybrid FES consisted of 4 minutes of ACE at 30W combined with 2 minutes of FES manual warm-up, followed by incremental stages of 5W/min and 10W/min as described. | High SCI (≥T6)  ↑ V̇O_2peak_ (L/min)  Without strategy = 1.23 ± 0.40  With strategy = 1.80 ± 0.40  ↑ V̇O_2peak_ (mL/kg/min)  Without strategy = 17.6 ± 5.0  With strategy = 24.4 ± 4.1  ↑ Power output (W)  Without strategy = 72.5 ± 32  With strategy = 83 ± 31  Low SCI (<T6)  ↔ V̇O_2peak_ (L/min)  Without strategy = 1.74 ± 0.24  With strategy = 1.90 ± 0.38  ↔ V̇O_2peak_ (mL/kg/min)  Without strategy = 23.7 ± 3.6  With strategy = 25.6 ± 4.1  ↔ Power output (W)  Without strategy = 96 ± 23  With strategy = 98 ± 19 | High SCI (≥T6)  ↑ Ο_2_ pulse (mL/beat)  Without strategy = 8.3 ± 1.7  With strategy = 11.0 ± 2.0  ↔ HR_peak_ (bpm)  Without strategy = 149 ± 34  With strategy = 164 ± 20  ↑ V̇_E_ (L/min)  Without strategy = 50.4 ± 20.8  With strategy = 61.4 ± 19.8  ↔ RER  Without strategy = 1.14 ± 0.07  With strategy = 1.18 ± 0.07  ↔ RPE  Without strategy = 18 ± 1  With strategy = 19 ± 1  ↑ [La^-^]_b_ (mmol/L)  Without strategy = 7.5 ± 1.1  With strategy = 9.4 ± 1.7  Low SCI (<T6)  ↔ Ο_2_ pulse (mL/beat)  Without strategy = 9.4 ± 0.9  With strategy = 10.4 ± 1.8  ↔ HR_peak_ (bpm)  Without strategy = 185 ± 11  With strategy = 182 ± 9  ↔ V̇_E_ (L/min)  Without strategy = 76.6 ± 13.4  With strategy = 80.2 ± 21.1  ↔ RER  Without strategy = 1.25 ± 0.11  With strategy = 1.23 ± 0.07  ↔ RPE  Without strategy = 19 ± 1  With strategy = 19 ± 1  ↔ [La^-^]_b_ (mmol/L)  Without strategy = 9.3 ± 0.9  With strategy = 10.6 ± 2.5 |
| ***Spinal cord stimulation*** | | | | | |
| Hodgkiss  2024  Canada  [27] | *N* = 2 (2M / 0F)  *Age* = 42 ± 25 (24 – 59) years  *TSI* = 4 ± 1 (3 – 5) years  *Classification* = 1T / 1P  *LOI* = C6 – T4  *Severity* = AIS A – B  *Physical activity level* = Excellent to good V̇O_2peak_ classification. | Ergogenic strategy  Epidural spinal cord stimulation (specific electrode configurations and stimulation parameters that were optimised for improved cardiovascular control)  *Participant 1: pulse width 500 µs, frequency 30 Hz, intensity 6.1 mA.*  *Participant 2: pulse width 380 µs, frequency 300 Hz, intensity 3.6 mA.*  Control strategy  Sham stimulation  (specific electrode configurations and stimulation parameters that were **not** optimised for improved cardiovascular control)  *Participant 1: pulse width 500 µs, frequency 30 Hz, intensity 2.9 mA.*  *Participant 2: pulse width 220 µs, frequency 40 Hz, intensity 4.2 mA.* | ACE to exhaustion at a constant workload corresponding to at or above ventilatory threshold, prescribed individually. | Time (mins)  Without strategy = 39 ± 15  With strategy = 54 ± 20  Time (s)  Without strategy = 2334 ± 891  With strategy = 3263 ± 1205  V̇O_2_ (L/min)  Without strategy = 1.09 ± 0.12  With strategy = 1.22 ± 0.12  V̇O_2_ (mL/kg/min)  Without strategy = 14.6 ± 0.3  With strategy = 16.3 ± 0.6 | Δ SBP (mmHg)  Without strategy = 1 ± 2  With strategy = 12 ± 1  Peak Ο_2_ pulse (mL/beat)  Without strategy = 12.3 ± 4.4  With strategy = 14.4 ± 4.9  HR_peak_ (bpm)  Without strategy = 131 ± 36  With strategy = 133 ± 35  V̇_E_ (L/min)  Without strategy = 33.5 ± 5.6  With strategy = 36.1 ± 6.0  RER  Without strategy = 0.96 ± 0.01  With strategy = 0.95 ± 0.06  RPE (Borg 6-20)  Without strategy = 17 ± 0  With strategy = 15 ± 3 |
| Hodgkiss  2024  Canada  [27] | *N* = 2 (2M / 0F)  *Age* = 47 ± 10 (40 – 54) years  *TSI* = 13 ± 9 (6 – 19) years  *Classification* = 0T / 2P  *LOI* = T4  *Severity* = AIS A  *Physical activity level* = Excellent to fair V̇O_2peak_ classification. | Ergogenic strategy  Transcutaneous spinal cord stimulation (specific cathode electrode locations and stimulation parameters that were optimised for improved cardiovascular control)  *Participant 1: T11 and L1 cathode placement, frequency 30 Hz burst + 10 kHz carrier, pulse width 1.0 ms, intensity 39 mA.*  *Participant 2: T11 and L1 cathode placement, frequency 30 Hz burst + 10 kHz carrier, pulse width 1.0 ms, intensity 78 mA.*  Control strategy  Sham stimulation  (specific cathode electrode locations and stimulation parameters that were **not** optimised for improved cardiovascular control)  *Intensity was increased to sensory threshold and slowly decreased to 0 mA prior to the start of the trial.* |  | Time (mins)  Without strategy = 37 ± 20  With strategy = 54 ± 19  Time (s)  Without strategy = 2199 ± 1221  With strategy = 3227 ± 1164  V̇O_2_ (L/min)  Without strategy = 1.16 ± 0.02  With strategy = 1.65 ± 0.68  V̇O_2_ (mL/kg/min)  Without strategy = 20.5 ± 9.5  With strategy = 21.7 ± 10.8 | Δ SBP (mmHg)  Without strategy = 1 ± 2  With strategy = 12 ± 1  Peak Ο_2_ pulse (mL/beat)  Without strategy = 12.3 ± 4.4  With strategy = 14.4 ± 4.9  HR_peak_ (bpm)  Without strategy = 131 ± 36  With strategy = 133 ± 35  V̇_E_ (L/min)  Without strategy = 33.5 ± 5.6  With strategy = 36.1 ± 6.0  RER  Without strategy = 0.96 ± 0.01  With strategy = 0.95 ± 0.06  RPE (Borg 6-20)  Without strategy = 17 ± 0  With strategy = 15 ± 3 |
| Nightingale  2019  Canada  [28] | *N* = 1 (1M / 0F)  *Age* = 33 years  *TSI* = 5 years  *Classification* = 1T / 0P  *LOI* = C5  *Severity* = AIS B  *Physical activity level* = NR | Ergogenic strategy  Epidural spinal cord stimulation (cardiovascular program, high-intensity)  *Pulse width 300 µs, frequency 35 Hz, intensity 6 V.*  Control strategy  No stimulation  *An average across two control sessions was calculated.* | Incremental ACE test to volitional exhaustion. Following a 2-min warm-up at 0W, workload was increased by 10W/min until volitional exhaustion. | V̇O_2peak_ (L/min)  Without strategy = 0.78 ± 0.01  With strategy = 0.95  V̇O_2peak_ (mL/kg/min)  Without strategy = 9.36 ± 0.25  With strategy = 11.08 | Δ MAP (mmHg)  Without strategy = -0.5 ± 2  With strategy = 15  RPE at 60W (Borg 6-20)  Without strategy = 18 ± 3  With strategy = 15 |
| Nightingale  2019  Canada  [28] | *N* = 1 (1M / 0F)  *Age* = 33 years  *TSI* = 5 years  *Classification* = 1T / 0P  *LOI* = C5  *Severity* = AIS B  *Physical activity level* = NR | Ergogenic strategy  Epidural spinal cord stimulation (cardiovascular program, low-intensity)  *Pulse width 300 µs, frequency 35 Hz, intensity 3.5 V.*  Control strategy  No stimulation  *An average across two control sessions was calculated.* | Incremental ACE test to volitional exhaustion. Following a 2-min warm-up at 0W, workload was increased by 10W/min until volitional exhaustion. | V̇O_2peak_ (L/min)  Without strategy = 0.78 ± 0.01  With strategy = 0.97  V̇O_2peak_ (mL/kg/min)  Without strategy = 9.36 ± 0.25  With strategy = 11.83 | O_2_ pulse (mL/beat)  Without strategy = 6.94 ± 0.14  With strategy = 7.82  Δ MAP (mmHg)  Without strategy = -0.5 ± 2  With strategy = 14  RPE at 60W (Borg 6-20)  Without strategy = 18 ± 3  With strategy = 15 |
| Nightingale  2019  Canada  [28] | *N* = 1 (1M / 0F)  *Age* = 33 years  *TSI* = 5 years  *Classification* = 1T / 0P  *LOI* = C5  *Severity* = AIS B  *Physical activity level* = NR | Ergogenic strategy  Epidural spinal cord stimulation (abdominal program, high-intensity)  *Pulse width 420 µs, frequency 40 Hz, intensity 6 V.*  Control strategy  No stimulation  *An average across two control sessions was calculated.* | Incremental ACE test to volitional exhaustion. Following a 2-min warm-up at 0W, workload was increased by 10W/min until volitional exhaustion. | V̇O_2peak_ (L/min)  Without strategy = 0.78 ± 0.01  With strategy = 0.95  V̇O_2peak_ (mL/kg/min)  Without strategy = 9.36 ± 0.25  With strategy = 11.78 | O_2_ pulse (mL/beat)  Without strategy = 6.94 ± 0.14  With strategy = 8.41  Δ MAP (mmHg)  Without strategy = -0.5 ± 2  With strategy = -0.5  RPE at 60W (Borg 6-20)  Without strategy = 18 ± 3  With strategy = 14 |
| Nightingale  2019  Canada  [28] | *N* = 1 (1M / 0F)  *Age* = 33 years  *TSI* = 5 years  *Classification* = 1T / 0P  *LOI* = C5  *Severity* = AIS B  *Physical activity level* = NR | Ergogenic strategy  Epidural spinal cord stimulation (abdominal program, low-intensity)  *Pulse width 420 µs, frequency 40 Hz, intensity 3.5 V.*  Control strategy  No stimulation  *An average across two control sessions was calculated.* | Incremental ACE test to volitional exhaustion. Following a 2-min warm-up at 0W, workload was increased by 10W/min until volitional exhaustion. | V̇O_2peak_ (L/min)  Without strategy = 0.78 ± 0.01  With strategy = 0.91  V̇O_2peak_ (mL/kg/min)  Without strategy = 9.36 ± 0.25  With strategy = 10.81 | O_2_ pulse (mL/beat)  Without strategy = 6.94 ± 0.14  With strategy = 7.52  Δ MAP (mmHg)  Without strategy = -0.5 ± 2  With strategy = -0.5  RPE at 60W (Borg 6-20)  Without strategy = 18 ± 3  With strategy = 15 |
| ***Pharmaceuticals, supplements or stimulants*** | | | | | |
| Flueck  2019  Switzerland  [29] | *N* = 8 (8M / 0F)  *Age* = 41 ± 11 years  *TSI* = NR  *Classification* = 2T / 6P  *LOI* = C4-L4  *Severity* = NR  *Physical activity level* = National level paracyclists, training 11 ± 4 hours split across 7 ± 2 sessions per week. | Ergogenic strategy  Sodium nitrate  *The sodium nitrate (6 mmoL nitrate) supplement was prepared by adding 510 mg of sodium nitrate (Pure sodium nitrate, POCH S.A., supplier: Pharmaserv LTD, Stansstad, Switzerland) in 85 mL of plain water. Only 60% of athletes could distinguish between the sodium nitrate and placebo supplements.*  Control strategy  Placebo  *Plain water.* | Simulated 10 km time trial (0.5% incline) on a handcycle ergometer with handibike. | ↔ V̇O_2average_ (mL/kg/min)  Without strategy = 35.2 ± 9.9  With strategy = 35.4 ± 9.5    ↔ Power output average (W)  Without strategy = 142 ± 49  With strategy = 144 ± 44    ↔ Time to complete (s)  Without strategy = 1106 ± 247  With strategy = 1091 ± 235 | ↔ HR_average_ (bpm)  Without strategy = 162 ± 27  With strategy = 158 ± 30  ↔ HR_max_ (bpm)  Without strategy = 176 ± 24  With strategy = 170 ± 31  ↔ RPE (Borg 6-20)  Without strategy = 19 (17;20)  With strategy = 19 (17;20)  ↔ [La^-^]_b_ (mmol/L)  Without strategy = 10.13 ± 5.98  With strategy = 10.05 ± 6.37 |
| Flueck  2019  Switzerland  [29] | *N* = 8 (8M / 0F)  *Age* = 41 ± 11 years  *TSI* = NR  *Classification* = 2T / 6P  *LOI* = C4-L4  *Severity* = NR  *Physical activity level* = National level paracyclists, training 11 ± 4 hours split across 7 ± 2 sessions per week. | Ergogenic strategy  Beetroot juice  *Beetroot juice (6 mmoL nitrate) was produced and delivered in the form of a standardised shot (Biotta AG, Tagerwilem, Switzerland). This was then bottled in an 85 mL non-transparent flask. All athletes were able to distinguish beetroot juice apart from the placebo, given its taste and colour.*  Control strategy  Placebo  *Plain water.* | Simulated 10 km time trial (0.5% incline) on a handcycle ergometer with handibike. | ↔ V̇O_2average_ (mL/kg/min)  Without strategy = 35.2 ± 9.9  With strategy = 34.7 ± 10.3  ↔ Power output average (W)  Without strategy = 142 ± 49  With strategy = 145 ± 42  ↔ Time to complete (s)  Without strategy = 1106 ± 247  With strategy = 1071 ± 199 | ↔ HR_average_ (bpm)  Without strategy = 162 ± 27  With strategy = 158 ± 31  ↔ HR_max_ (bpm)  Without strategy = 176 ± 24  With strategy = 173 ± 28  ↔ RPE (Borg 6-20)  Without strategy = 19 (17;20)  With strategy = 19 (17;20)  ↔ [La^-^]_b_ (mmol/L)  Without strategy = 10.13 ± 5.98  With strategy = 9.92 ± 5.99 |
| Klimesova  2017  Czech Republic  [30] | *N* = 7 (7M / 0F)  *Age* = 28.0 ± 5.4 years  *TSI* = 9.6 ± 5.6 years  *Classification* = 6T / 1P  *LOI* = C6-T1  *Severity* = 7 comp / 0 incomp  *Physical activity level* = Elite wheelchair rugby athletes with 4.0 (range: 1.5 - 11) years experience | Ergogenic strategy  Caffeine capsule  *Participants received gelatine capsules packaged in either 30mg or 15mg anhydrous caffeine (Nutrend D.S., Olomuc, Czech Republic). Dosage was weight-dependent (3mg/kg). Capsules were swallowed with 500 mL of tap water.*  Control strategy  Placebo  *Placebo capsules contained inert microcrystallines. Participants received the same number of capsules as the caffeine trial. Capsules were swallowed with 500 mL of tap water.* | Incremental ACE to volitional exhaustion. Following a warm-up between 20 – 40W, the test began at a workload of 50W and increased incrementally by 5W every minute until exhaustion.  **Note, test duration and RER are reported as median (range).* | ↔ V̇O_2 peak_ (mL/kg/min)  Without strategy = 11.35 ± 2.6  With strategy = 12.02 ± 2.33  ↔ Maximum power (W/kg)  Without strategy = 0.83 ± 0.15  With strategy = 0.83 ± 0.14  ↔ Test duration (minutes)  Without strategy = 4.37 (0.38 - 10.0)  With strategy = 5.80 (0.36 - 10.0) | ↔ HR_peak_ (bpm)  Without strategy = 106 ± 13  With strategy = 111 ± 22  ↔ RPE (6-20)  Without strategy = 16 ± 1  With strategy = 16 ± 1  ↔ RER  Without strategy = 1.13 (1.03 - 1.19)  With strategy = 1.14 (1.03 - 1.21) |
| Nieshoff  2004  USA  [31] | *N* = 4 (3M / 1F)  *Age* = 35.3 ± 5.9 years  *TSI* = 9.6 ± 5.5 years  *Classification* = 4T / 0P  *LOI* = C6-C8  *Severity* = 4 comp / 0 incomp  *Physical activity level* = One participant was involved in a regular exercise programme, while the remaining participants were sedentary other than performing activities of daily living. | Ergogenic strategy  Midodrine 5mg  *Consumed orally in an unmarked capsule, 60-min prior to testing.*  Control strategy  Placebo  *Consumed orally in an unmarked capsule, 60-min prior to testing.* | Discontinuous, symptom-limited exercise test on a wheelchair ergometer. Following a 2-3 min warm-up at 0W, workload was increased by 5W/min. Every 3-min participants were permitted a 2-3 min rest period to delay local fatigue.  No statistical analysis. | V̇O_2peak_ (mL/kg/min)  Without strategy = 12.34 ± 1.18  With strategy = 12.76 ± 1.96 | HR_peak_ (bpm)  Without strategy = 117 ± 15  With strategy = 118 ± 23  SBP_peak_ (mmHg)  Without strategy = 100 ± 12  With strategy = 132 ± 14  RPE (Borg 6-20)  Without strategy = 16 ± 1  With strategy = 14 ± 1 |
| Nieshoff  2004  USA  [31] | *N* = 4 (3M / 1F)  *Age* = 35.3 ± 5.9 years  *TSI* = 9.6 ± 5.5 years  *Classification* = 4T / 0P  *LOI* = C6-C8  *Severity* = 4 comp / 0 incomp  *Physical activity level* = One participant was involved in a regular exercise programme, while the remaining participants were sedentary other than performing activities of daily living. | Ergogenic strategy  Midodrine 10mg  *Consumed orally in an unmarked capsule, 60-min prior to testing.*  Control strategy  Placebo  *Consumed orally in an unmarked capsule, 60-min prior to testing.* | Discontinuous, symptom-limited exercise test on a wheelchair ergometer. Following a 2-3 min warm-up at 0W, workload was increased by 5W/min. Every 3-min participants were permitted a 2-3 min rest period to delay local fatigue. | V̇O_2peak_ (mL/kg/min)  Without strategy = 12.34 ± 1.18  With strategy = 13.44 ± 3.56 | HR_peak_ (bpm)  Without strategy = 117 ± 15  With strategy = 120 ± 22  SBP_peak_ (mmHg)  Without strategy = 100 ± 12  With strategy = 145 ± 41  RPE (Borg 6-20)  Without strategy = 16 ± 1  With strategy = 15 ± 2 |
| Perret  2020  Switzerland  [32] | *N* = 27 (27M / 0F)  *Age* = 40 (21-54) years (median, range)  *TSI* = 151 (5-427) months (median, range)  *Classification* = NR  *LOI* = C5-l3  *Severity* = 27 comp / 0 incomp  *Physical activity level* = Wheelchair athletes | Ergogenic strategy  Sildenafil citrate  *One hour before testing, participants consumed a capsule containing 50mg sildenafil citrate with 300 – 500 mL water.*  Control strategy  Placebo  *One hour before testing, participants consumed a placebo capsule (mannitol) with 300 – 500 mL water.* | Incremental ACE to volitional exhaustion. Following a 2-min warm-up at 20W, workload was increased by 5W every 30s until volitional exhaustion.  *Tests were performed at sea-level and altitude (2200m), however only responses to exercise at sea-level are reported here. Data are reported as median (interquartile range) or mean ± SD.* | ↔ V̇O_2peak_ (mL/kg/min)  Without strategy = 28.5 (13,40.1)  With strategy = 26 (11,46.4)  ↓ Peak power (W)  Without strategy = 119 (107, 137)  With strategy = 114 (104, 137) | ↔ SpO_2_ (%)  Without strategy = 98.03 ± 7.03  With strategy = 98.02 ± 4.45  ↔ HR_peak_ (bpm)  Without strategy = 174 (99, 193)  With strategy = 174 (91, 192)  ↔ RPE (Borg 6-20)  Without strategy = 18 (14, 20)  With strategy = 18 (14, 20)  ↔ Lactate (mmol/L)  Without strategy = 6.3 (1.4,12.7)  With strategy = 7.5 (1.4,10.8) |
| Data are reported as Mean ± SD, unless stated otherwise. ↑ = significantly greater relative to control strategy; ↓ = significantly lower relative to control; ↔ = no change or inconclusive. Outcomes without arrows were either calculated by reviewers from data included in the paper or statistical analysis was not reported to determine an effect. Some studies included multiple ergogenic strategies, hence why studies appear more than once. ACE, arm-crank ergometry; comp, motor-complete; F, females; FES, functional electrical stimulation; incomp, motor-incomplete; LOI, level of injury; M, males; NR, not reported; P, paraplegia; PPO, peak power output; SCI, spinal cord injury; SV, stroke volume; T, tetraplegia; VO_2_, oxygen consumption; W, Watts. | | | | | |

**REFERENCES**

1. Kerk JK, Clifford PS, Snyder AC, Prieto TE, O’Hagan KP, Schot PK, et al. Effect of an abdominal binder during wheelchair exercise. Med Sci Sports Exerc. 1995;27: 913–919.

2. West CR, Goosey-Tolfrey VL, Campbell IG, Romer LM. Effect of abdominal binding on respiratory mechanics during exercise in athletes with cervical spinal cord injury. J Appl Physiol. 2014;117: 36–45.

3. Brurok B, Tørhaug T, Leivseth G, Karlsen T, Helgerud J, Hoff J. Effect of leg vascular occlusion on arm cycling peak oxygen uptake in spinal cord-injured individuals. Spinal Cord. 2012;50: 298–302.

4. Hopman MT, Oeseburg B, Binkhorst RA. The effect of an anti-G suit on cardiovascular responses to exercise in persons with paraplegia. Med Sci Sports Exerc. 1992;24: 984–990.

5. Hopman MT, Dueck C, Monroe M, Philips WT, Skinner JS. Limits to maximal performance in individuals with spinal cord injury. Int J Sports Med. 1998;19: 98–103.

6. Houtman S, Thielen JJ, Binkhorst RA, Hopman MT. Effect of a pulsating anti-gravity suit on peak exercise performance in individual with spinal cord injuries. Eur J Appl Physiol Occup Physiol. 1999;79: 202–204.

7. Kaprielian R, Plyley MJ, Klentrou P, Goodman LS, Goodman JM. The effect of lower body positive pressure on the cardiovascular response to exercise in sedentary and endurance-trained persons with paraplegia. Eur J Appl Physiol Occup Physiol. 1998;78: 141–147.

8. Pitetti KH, Barrett PJ, Campbell KD, Malzahn DE. The effect of lower body positive pressure on the exercise capacity of individuals with spinal cord injury. Med Sci Sports Exerc. 1994;26: 463–468.

9. Rimaud D, Calmels P, Roche F, Mongold J-J, Trudeau F, Devillard X. Effects of graduated compression stockings on cardiovascular and metabolic responses to exercise and exercise recovery in persons with spinal cord injury. Arch Phys Med Rehabil. 2007;88: 703–709.

10. Vaile J, Stefanovic B, Askew CD. Effect of lower limb compression on blood flow and performance in elite wheelchair rugby athletes. J Spinal Cord Med. 2016;39: 206–211.

11. Tørhaug T, Brurok B, Hoff J, Helgerud J, Leivseth G. Arm Cycling Combined with Passive Leg Cycling Enhances VO2peak in Persons with Spinal Cord Injury Above the Sixth Thoracic Vertebra. Top Spinal Cord Inj Rehabil. 2018;24: 86–95.

12. West CR, Currie KD, Gee C, Krassioukov AV, Borisoff J. Active-Arm Passive-Leg Exercise Improves Cardiovascular Function in Spinal Cord Injury. Am J Phys Med Rehabil. 2015;94: e102-6.

13. Hooker SP, Greenwood JD, Boyd LA, Hodges MR, McCune LD, McKenna GE. Influence of posture on arm exercise tolerance and physiologic responses in persons with spinal cord injured paraplegia. Eur J Appl Physiol Occup Physiol. 1993;67: 563–566.

14. Burnham R, Wheeler G, Bhambhani Y, Belanger M. Intentional induction of autonomic dysreflexia among quadriplegic athletes for performance enhancement: efficacy, safety, and mechanism of action. Clin J Sport Med. 1994;4: 1–1.

15. Gee CM, Lacroix MA, West CR. Effect of Unintentional Boosting on Exercise Performance in a Tetraplegic Athlete. Med Sci Sports Exerc. 2018;50: 2398–2400.

16. Nightingale TE, Eginyan G, Balthazaar SJT, Williams AMM, Lam T, Krassioukov AV. Accidental boosting in an individual with tetraplegia - considerations for the interpretation of cardiopulmonary exercise testing. J Spinal Cord Med. 2022; 1–6.

17. Schmid A, Schmidt-Trucksäss A, Huonker M, König D, Eisenbarth I, Sauerwein H, et al. Catecholamines response of high performance wheelchair athletes at rest and during exercise with autonomic dysreflexia. Int J Sports Med. 2001;22: 2–7.

18. Bakkum AJT, de Groot S, Onderwater MQ, de Jong J, Janssen TWJ. Metabolic rate and cardiorespiratory response during hybrid cycling versus handcycling at equal subjective exercise intensity levels in people with spinal cord injury. J Spinal Cord Med. 2014;37: 758–764.

19. Brurok B, Tørhaug T, Karlsen T, Leivseth G, Helgerud J, Hoff J. Effect of lower extremity functional electrical stimulation pulsed isometric contractions on arm cycling peak oxygen uptake in spinal cord injured individuals. J Rehabil Med. 2013;45: 254–259.

20. Hasnan N, Ektas N, Tanhoffer AIP, Tanhoffer R, Fornusek C, Middleton JW, et al. Exercise responses during functional electrical stimulation cycling in individuals with spinal cord injury. Med Sci Sports Exerc. 2013;45: 1131–1138.

21. Hooker SP, Figoni SF, Rodgers MM, Glaser RM, Mathews T, Suryaprasad AG, et al. Metabolic and hemodynamic responses to concurrent voluntary arm crank and electrical stimulation leg cycle exercise in quadriplegics. J Rehabil Res Dev. 1992;29: 1–11.

22. Laskin JJ, Ashley EA, Olenik LM, Burnham R, Cumming DC, Steadward RD, et al. Electrical stimulation-assisted rowing exercise in spinal cord injured people. A pilot study. Paraplegia. 1993;31: 534–541.

23. Phillips W, Burkett LN. Arm crank exercise with static leg FNS in persons with spinal cord injury. Med Sci Sports Exerc. 1995;27: 530–535.

24. Raymond J, Davis GM, Fahey A, Climstein M, Sutton JR. Oxygen uptake and heart rate responses during arm vs combined arm/electrically stimulated leg exercise in people with paraplegia. Spinal Cord. 1997;35: 680–685.

25. Raymond J, Davis GM, Climstein M, Sutton JR. Cardiorespiratory responses to arm cranking and electrical stimulation leg cycling in people with paraplegia. Med Sci Sports Exerc. 1999;31: 822–828.

26. Shaffer RF, Picard G, Taylor JA. Relationship of Spinal Cord Injury Level and Duration to Peak Aerobic Capacity With Arms-Only and Hybrid Functional Electrical Stimulation Rowing. Am J Phys Med Rehabil. 2018;97: 488–491.

27. Hodgkiss DD, Williams AMM, Shackleton CS, Samejima S, Balthazaar SJT, Lam T, et al. Ergogenic effects of spinal cord stimulation on exercise performance following spinal cord injury. Front Neurosci. 2024;18: 1435716.

28. Nightingale TE, Walter M, Williams AMM, Lam T, Krassioukov AV. Ergogenic effects of an epidural neuroprosthesis in one individual with spinal cord injury. Neurology. 2019;92: 338–340.

29. Flueck JL, Gallo A, Moelijker N, Bogdanov N, Bogdanova A, Perret C. Influence of Equimolar Doses of Beetroot Juice and Sodium Nitrate on Time Trial Performance in Handcycling. Nutrients. 2019;11. doi:10.3390/nu11071642

30. Klimešová I, Machová I, Jakubec A, Corkle J. Effect of caffeine on maximal oxygen uptake in wheelchair rugby players: A randomized, placebo-controlled, double-blind study. Acta Gymnica. 2017;47: 16–23.

31. Nieshoff EC, Birk TJ, Birk CA, Hinderer SR, Yavuzer G. Double-blinded, placebo-controlled trial of midodrine for exercise performance enhancement in tetraplegia: a pilot study. J Spinal Cord Med. 2004;27: 219–225.

32. Perret C, Van Biesen D, Strupler M, Pit-Grosheide P, Vanlandewijck Y. Effect of Sildenafil Citrate on Exercise Capacity in Athletes With Spinal Cord Injury. Int J Sports Physiol Perform. 2020;15: 971–975.
